# Optimizing the white light emission in the solid state isatin and thiazole based molecular hybrids by introduction of variety of substituents on isatin and thiazole ring systems†

**DOI:** 10.1039/d4ra09010a

**Published:** 2025-03-14

**Authors:** Sultana Shaik, Rama Mohana Reddy Sirigireddy, Sai Teja Talari, Haranath Divi, Naveen Mulakayala, Venkatramu Vemula, Chinna Gangi Reddy Nallagondu

**Affiliations:** a Green & Sustainable Synthetic Organic Chemistry and Optoelectronics Laboratory, Department of Chemistry, Yogi Vemana University Kadapa-516005 Andhra Pradesh India ncgreddy@yogivemanauniversity.ac.in ncgreddy@yvu.edu.in; b Department of Physics, Yogi Vemana University Kadapa-516 005 Andhra Pradesh India vvramuphd@gmail.com; c Department of Physics, National Institute of Technology Warangal-506004 Telangana India; d SVAK Lifesciences ALEAP Industrial Area, Pragathi Nagar Hyderabad 500090 India

## Abstract

An efficient and practical 3-component reaction strategy has been developed for the synthesis of a series of multi-colour emissive isatin–thiazole based fluorophores, thiazolylhydrazonoindolin-2-ones (4) from readily available isatins (1), thiosemicarbazide (2) and α-bromoketones (3) in the presence of biodegradable citric acid (0.1 N) in ethanol at reflux temperature for 40–60 min. The reaction proceeds *via* condensation (C=N) and subsequent heterocyclization (C–S & C–N) in one-pot. Nature-friendly reaction profile, easy to perform, wide substrate scope, use of non-hazardous solvents/catalysts, good functional group tolerance, excellent yields (91–98%) in short reaction times, scalability and products do not require column chromatography purification are the attractive features of the present MCR strategy. The photophysical properties of the titled compounds (4) in both solid and solution states have been evaluated. The study reveals that the prepared isatin–thiazole based molecular hybrids exhibited tunable photophysical properties by varying the substituents on both isatin and thiazole motifs. To our delight, the titled compounds, 4k, 4l, 4m, 4u and 4y displayed white light emission with mega Stokes shifts in the solid state.

## Abstract 2

Citric acid catalyzed 3-component reaction strategy has been developed for the synthesis of multi-colour emissive isatin–thiazole based fluorophores (4) from isatins (1), thiosemicarbazide (2) and α-bromoketones (3) in ethanol under reflux conditions.

## Graphical abstract

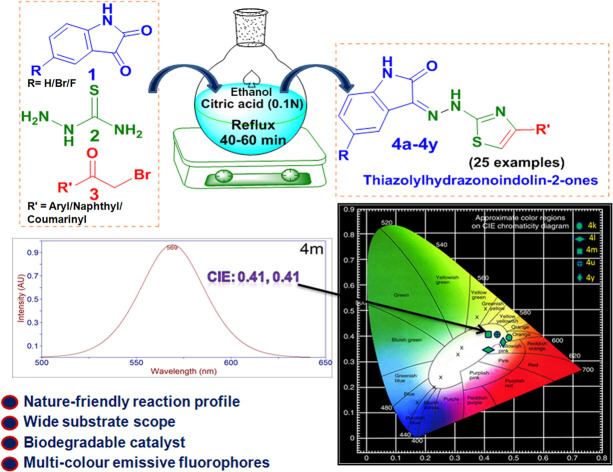

## Introduction

Small organic luminophores emitting multicolour fluorescence in the solid state have a wide range of applications in full-color display panels and light sources.^1–9^ On the other hand, development of solid state light emissive fluorophores is really exigent assignment due to the aggregation-caused quenching (ACQ) in organic fluorophores. In this perspective, the intramolecular charge transfer (ICT) in organic luminophores is one of the challenging tasks for optoelectronic materials owing to the possibility for fine tuning of photophysical properties in the solid state.^10–12^ The photophysical properties of solid state emissive organic fluorophores which includes absorption, emission, Stokes shifts, Commission Internationale d'Eclairage (CIE) coordinates and correlated colour temperature (CCT) have played a pivotal role in the fabrication of various optoelectronic devices like organic light-emitting diodes (OLEDs), organic solid state lasers and many others. Particularly, solid state white-light emitting small organic fluorophores (SWESOFs) received substantial societal attentions due to their unique merits like high efficiency, light-weight, longevity, high color quality, low-voltage operation, super-thin thickness, excellent compatibility, mercury-free device fabrication *etc.*, which signifying great affirm for solid state lighting (SSL) applications.^13–21^ Usually, white light emission is achieved mainly by integrating the stoichiometric ratios of primary colours of red, green and blue or blue and yellow. The CIE coordinates (*x*, *y*) for ideal white light is (0.33, 0.33), which is good for human eyesight. Hence, great efforts are devoted in both industry and academia for the development of solid state white light emitting small organic molecules with high fluorescence efficiency, low cost, low power consumption, high color purity, flexibility, ultra-thin thickness, stability, *etc.* Recently, we reported a few SWESOFs for optoelectronic applications.^22,23^ On the basis of our earlier studies on SWESOFs, herein, thiazole and isatin based small organic fluorophores have been designed and developed for white light emission in the solid state.

Heterocycles containing nitrogen and sulphur atoms are amongst the most studied groups of small organic molecule emitters for example carbazoles, diazoles, triazoles, thiazoles, thiophenes *etc.*,^24^ which have been utilized as efficient fluorescent organic materials for making optoelectronic devices, photovoltaics, *etc.*,^25–28^ because of their promising charge transfer and light-absorption properties. Further, two or more heterocyclic motifs containing small organic molecules are considered as another kind of compounds which display interesting optical properties.^29^ In this context, thiazoles and isatins have received great attention due to their promising applications in both organic electronics and biological sciences. Thiazole compounds display promising optoelectronic and biological applications because these heterocyclic cores employed as fluorescent materials and key starting materials in the synthesis of lead molecules, new drug candidates, medicinally active compounds *etc*.^22,23,30–35^ In addition, this nucleus is found in several pharmaceutical drug molecules.^36–39^ Isatin group of compounds exhibit a wide spectrum of biological and optoelectronic properties.^40–46^ This core moiety has been found in many drugs like sunitinib, toceranib and nintedanib.^47–49^ Further, thiazole and isatin containing molecular hybrids also act as potent α-glucosidase inhibitors, anti-proliferative, antiviral, anti-mycobacterial agents, *etc*.^50–52^ These molecular hybrids also act as hole transporting and ambipolar materials.^53^ This motivated us to investigate the photophysical properties of thiazole and isatin based dyes. Up to now, a very limited protocols have been reported on both multi-component and multi-step reaction strategies for the synthesis of thiazolylhydrazonoindolin-2-ones from isatins, thiosemicarbazide and α-bromoketones.^52,54–56^ Though the reported methodologies are efficient for providing thiazolylhydrazonoindolin-2-ones, but suffer from some drawbacks such as limited substrate scope, long reaction times, use of expensive and toxic catalysts *etc.* In view of the above, identification of an efficient, practical, green and biodegradable catalytic system which fulfills the above limitations is highly desirable and challenging task for the synthesis of titled compounds.

Citric acid is an organic acid which has great societal attentions because of its green merits which includes environmental-friendly nature, biodegradability, non-toxicity, excellent compatibility, inexpensiveness, *etc.* In spite of its nature-friendliness, a very few citric acid catalyzed organic transformations have been reported so far.^57–59^ Hence, there is a wide scope to investigate the catalytic efficiency of citric acid in a variety of organic conversions.

**Scheme 1:**
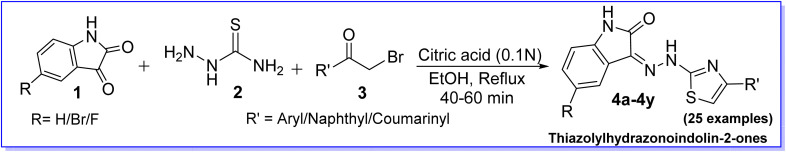
Citric acid (0.1 N) catalyzed 3-component reaction for the synthesis of thiazolylhydrazonoindolin-2-ones (4).

Herein, citric acid (0.1 N) identified as environment friendly and biodegradable catalyst for the multi-component synthesis of thiazolylhydrazonoindolin-2-ones (4) *via* condensation and heterocyclization from readily available isatins (1), thiosemicarbazide (2) and α-bromoketones (3) in ethanol at reflux temperature in a single step operation (Scheme 1). Further, photophysical properties of the synthesized isatin–thiazole based fluorophores (4) have been investigated in both solid and solution states.

## Results and discussion

Initially, a control experiment was performed to optimize the reaction conditions for the synthesis of thiazolylhydrazonoindolin-2-ones (4). For this purpose, isatin (1a), thiosemicarbazide (2) and ω-bromoacetophenone (3a) were chosen as ideal model substrates for the synthesis of 3-(2-(4-phenylthiazol-2-yl)hydrazineylidene)indolin-2-one (4a). At first, the model reaction was carried out in the absence of catalyst in H_2_O at room temperature (Table 1, entry 1), it was found that the product has not been formed. Thereafter, the same reaction was repeated by screening various parameters like catalysts, solvents, temperature, and time to get the desired product (4a). Later, the same reaction was conducted in the presence of 0.1 N acetic acid in different solvents *i.e*., acetone/isopropanol/ethanol (Table 1, entry 2). From this study, it was noticed that the reaction proceeded with a moderate yield (55%) of desired product (4a) in the presence of 0.1 N acetic acid (3.0 mL) in ethanol (3.0 mL) at reflux temperature in 90 min. Further, we aimed to develop the above 3-component reaction under green reaction conditions with environmental concerns in mind. To achieve the objective, various biodegradable α-hydroxy acid catalysts (malic acid, tartaric acid and citric acid) and different green solvents (acetone, isopropanol, and ethanol) were employed. The obtained results are summarized in Table 1. After the examination of various catalysts and solvents for the above-said reaction, it was found that the 3.0 mL of 0.1 N citric acid in EtOH (3.0 mL) was the most suitable catalytic medium to obtain high yield (80%) of the desired product (4a) at reflux temperature in 65 min (Table 1, entry 5). The other catalysts and solvents provided low to moderate yields of 4a (Table 1, entries 3 and 4). Further, the amount of 0.1 N citric acid was varied (3.5, 4.0, 4.5, 5.0, 5.5, and 6.0 mL) to improve the yield of 4a (Table 2). The study reveals that the 5.0 mL of 0.1 N citric acid in EtOH (3.0 mL) at reflux temperature afforded the 97% yield of 4a within 45 min (Table 2, entry 5).

**Table 1:** Optimization of reaction conditions for the synthesis of 3-(2-(4-phenylthiazol-2-yl)hydrazineylidene)indolin-2-one (4a)^a^

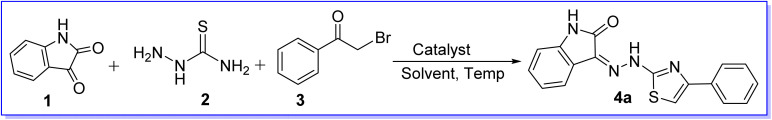						
Entry	Catalyst (3 mL)	Solvent (3 mL)	Temp. (°C)	Time (min)	Product	Yield^b^ (%)
1^c^	—	H_2_O	RT	180	4a	—
2	Acetic acid (0.1 N)	Acetone	RT	90	4a	—
		Isopropanol	Reflux	90		45
		EtOH	Reflux	90		55
3	Malic acid (0.1 N)	Acetone	RT	90	4a	—
		Isopropanol	Reflux	90		25
		EtOH	Reflux	90		35
4	Tartaric acid (0.1 N)	Acetone	RT	90	4a	—
		Isopropanol	Reflux	90		45
		EtOH	Reflux	90		60
5	**Citric acid (0.1 N)**	Acetone	RT	90	4a	—
		Isopropanol	Reflux	90		55
		**EtOH**	**Reflux**	**65**		**80**

a Reagents and conditions: isatin (1a) (5.0 mmol), thiosemicarbazide (2) (5.0 mmol), phenacyl bromide (3a) (5.0 mmol), catalyst (3 mL) & solvent (3 mL) at reflux/RT temperature.

b Isolated yield.

c Reaction performed in the absence of catalyst.

**Table 2:** Screening of catalyst loading in one-pot synthesis of 3-(2-(4-phenylthiazol-2-yl)hydrazineylidene)indolin-2-one (4a)^a^

Entry	Citric acid (0.1 N) (mL)	Time (min)	Product	Yield^b^ (%)
1	3.0	65	4a	80
2	3.5	60	4a	84
3	4.0	50	4a	89
4	4.5	45	4a	93
**5**	**5.0**	**45**	4a	**97**
6	5.5	45	4a	97
7	6.0	45	4a	97

a Reagents and conditions: isatin (1a) (5.0 mmol), thiosemicarbazide (2) (5.0 mmol), phenacyl bromide (3a) (5.0 mmol), 0.1 N citric acid (mL), EtOH (3 mL) at reflux temperature.

b Isolated yield.

The yields of the product did not change when increasing the volume of 0.1 N citric acid (Table 2, entries 6 and 7). From this study, it was concluded that the said 3-component reaction proceeded smoothly in the presence of 0.1 N citric acid in EtOH and afforded the maximum yield of target product 4a.

Under the established reaction conditions in hand, the substrate scope with respect to substituted isatins (1a, 1b & 1c) and α-bromoketones (3a–j) was studied to investigate the generality of this MCR strategy. The obtained results are summarized in Table 3. The reaction of isatin (1a) thiosemicarbazide (2) and phenacyl bromide (3a) afforded the corresponding product (4a) in excellent yield (97%). Similarly, phenacyl bromides substituted by activating groups (4-Me (3b), 4-OMe (3c)), deactivating groups (4-Cl (3e), 4-Br (3f), and 3-Br (3g)) and strongly deactivating groups (4-F (3d), 4-CN (3h)) provided the desired products (4b–h) in excellent yields (93–98%). Isatin (1a) reacted well with thiosemicarbazide (2) and 2-bromo-1-(naphthalen-2-yl)ethan-1-one (3i) also gave the corresponding product (4i) in good isolated yield (96%). Interestingly, isatin (1a) showed good reactivity with thiosemicarbazide (2) and 3-(2-bromoacetyl)-2*H*-chromen-2-one (3j) afforded the desired product (4j) in good isolated yield (95%).

**Table 3:** Synthesis of a library of thiazolylhydrazonoindolin-2-ones (4)^a^

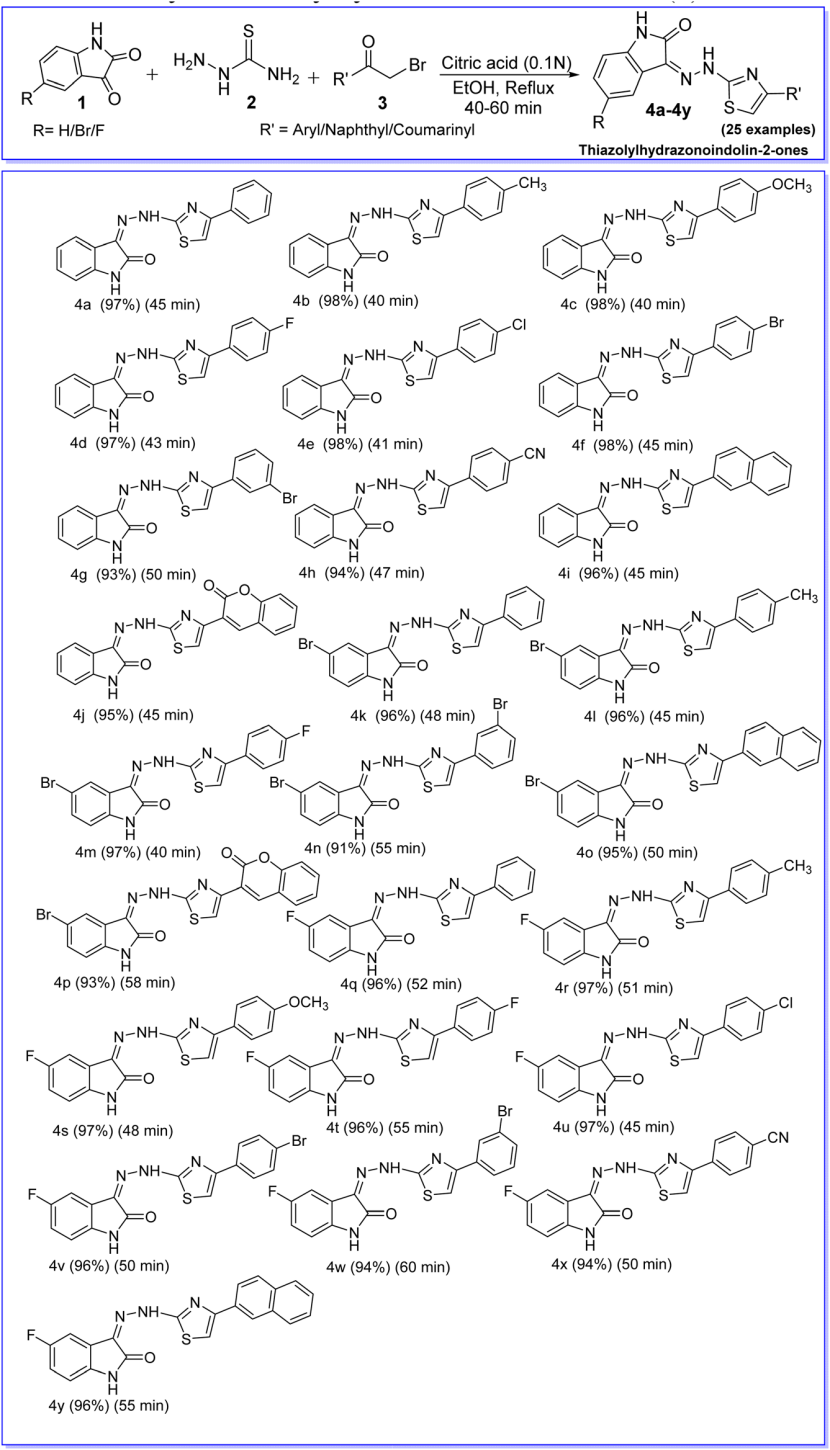

a Reagents and conditions: isatins (1) (5.0 mmol), thiosemicarbazide (2) (5.0 mmol), α-bromoketones (3) (5.0 mmol), 0.1 N citric acid (5.0 mL), EtOH (3.0 mL) at reflux temperature.

Further, 5-bromo isatin (1b) was also reacted well with thiosemicarbazide (2) and various α-bromoketones (3a, 3b, 3d, 3g, 3i and 3j) and produced the corresponding products (4k–4p) in good to excellent yields (91–97%), respectively. 5-Fluoro isatin (1c) was also showed good reactivity with thiosemicarbazide (3) and variety of α-bromoketones (3a–3i) provided the desired products (4q–4y) in excellent yields ranging from 94–97%. Hence, the present MCR strategy well tolerates various substituents on the aromatic ring of α-bromoketones and isatins.

### Scalability

**Fig. 1:**
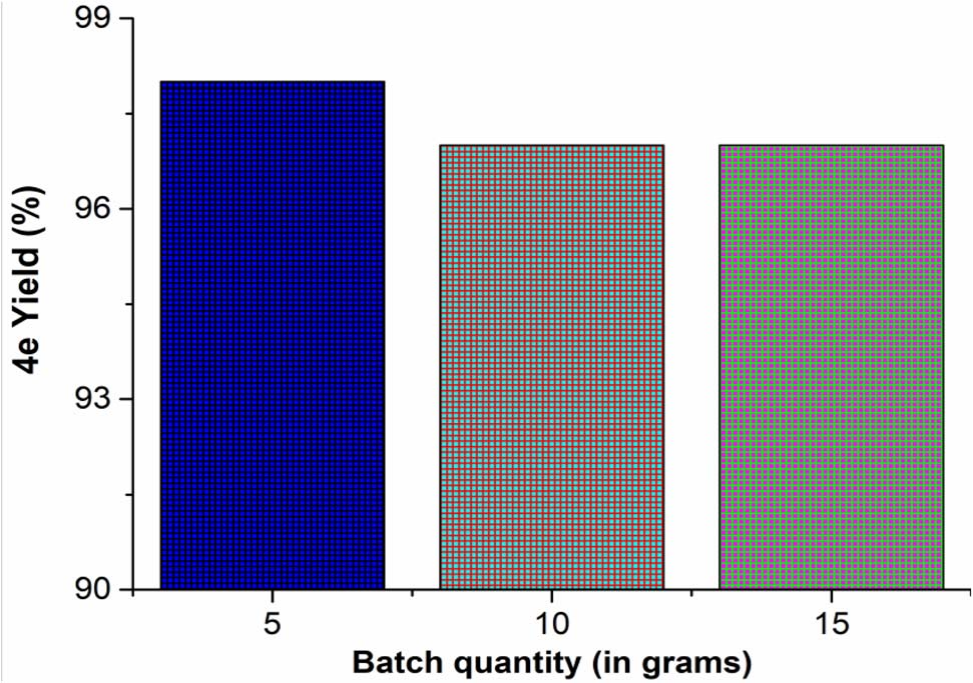
Gram-scale synthesis of 4e.

The scalability of the established procedure was then investigated by the reaction of isatin (1a), thiosemicarbazide (2) and 2,4′-dibromoacetophenone (3f) catalyzed by citric acid (0.1 N) in different gram-scale reactions (*i.e*., 5.0 10.0 and 15.0 g). The obtained yields of the product 4e were 98, 97, and 97%, respectively (Fig. 1). Hence, the developed 3-component reaction strategy is most suitable for the gram-scale production of thiazolylhydrazonoindolin-2-ones (4).

### Plausible mechanism

**Scheme 2:**
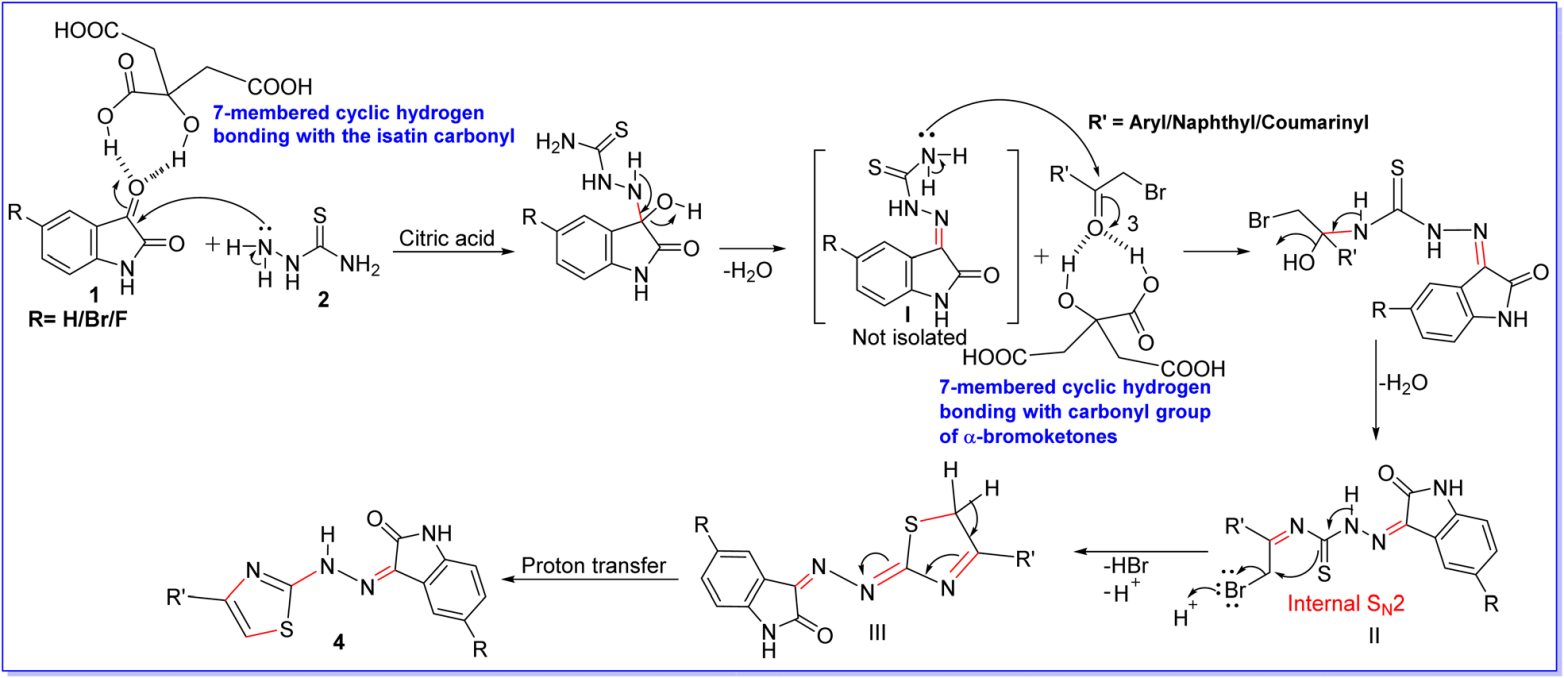
A plausible mechanism for the formation of thiazolylhydrazonoindolin-2-ones (4) in the presence of citric acid (0.1 N).

A plausible mechanism for the formation of thiazolylhydrazonoindolin-2-ones (4) in the presence of citric acid (0.1 N) is depicted in Scheme 2. Firstly, the NH_2_ group of thiosemicarbazide (2) reacts with citric acid (0.1 N) activated carbonyl group of isatins (1) to obtain thiosemicarbazone (I) *via* a simple condensation reaction and then there is a loss of water by the condensation reaction of thiosemicarbazone (I) and α-bromo ketones (3) to form diimine (II). Further, the intermediate (II) undergoes internal S_N_2 displacement of bromide ion in acid medium to form dihydrothiazolylhydrazonoindolin-2-ones (III) followed by a proton shift to obtain thiazolylhydrazonoindolin-2-ones (4). The high catalytic efficacy of citric acid in the present MCR might be due to the formation of 7-membered cyclic hydrogen bonding with both the carbonyl groups of isatins and α-bromo ketones.^60^

### Photophysical properties of thiazolylhydrazonoindolin-2-ones (4)

The photophysical properties of a library of synthesized titled compounds (4) were studied in both solid and solution states from their absorption spectra and luminescence spectra and are shown in Table 4. The solubility of the synthesized thiazolylhydrazonoindolin-2-ones (4) was examined in various polar solvents like isopropanol, acetone, acetonitrile, dichloromethane, ethyl acetate, dimethylformamide (DMF) and dimethyl sulfoxide (DMSO). The study reveals that the titled compounds (4) are freely soluble in polar aprotic DMSO at RT as compared to other solvents. Therefore, the photophysical properties of 4 in the solution state were investigated in DMSO. The Stokes shifts, CIE coordinates, CCT values and quantum yields were determined and discussed hereunder.

**Table 4:** Photophysical properties of thiazolylhydrazonoindolin-2-ones (4)

Entry	Comp.	*λ*_exi_ a (nm)	*λ*_abs_ b (nm)	*λ*_emi_ c (nm)		Stokes shift^d^ (cm^−1^ nm^−1^)		CIE coordinates				CCT^e^ (K)
								Solid		DMSO		
		Solid	DMSO	Solid	DMSO	Solid	DMSO	*x*	*y*	*x*	*y*	Solid
1	4a	368	352	553	432	9091/185	5261/80	0.41	0.57	0.19	0.21	—
2	4b	368	350	578	430	9873/210	5315/80	0.50	0.44	0.20	0.24	—
3	4c	370	369	580	534	9786/210	8373/165	0.51	0.44	0.26	0.54	—
4	4d	368	354	558	430	9253/190	4993/76	0.43	0.55	0.19	0.21	—
5	4e	368	352	559	433	9285/191	5314/81	0.43	0.55	0.17	0.16	—
6	4f	368	352	565	430	9475/197	5153/78	0.47	0.52	0.20	0.23	—
7	4g	370	352	542	428	8577/172	5044/76	0.36	0.60	0.18	0.18	—
8	4h	368	357	559	436	9285/191	5075/79	0.43	0.55	0.17	0.15	—
9	4i	370	362	557	451	9074/187	5451/89	0.41	0.50	0.18	0.21	—
10	4j	371	361	536	448	8297/165	5379/87	0.34	0.62	0.16	0.15	—
11	4k	348	346	586	420	11 671/238	5092/74	0.48	0.40	0.16	0.10	2225
12	4l	370	362	584	435	9904/214	4636/73	0.41	0.34	0.17	0.12	2913
13	4m	370	359	569	433	9452/199	4760/74	0.41	0.41	0.17	0.12	3554
14	4n	503	352	565	392	2182/62	2899/40	0.42	0.49	0.15	0.08	—
15	4o	372	397	589	518	9904/217	5884/121	0.50	0.41	0.23	0.62	—
16	4p	370	362	550	438	8845/180	4793/76	0.38	0.59	0.23	0.62	—
17	4q	370	362	582	440	9845/212	4897/78	0.5	0.47	0.16	0.13	—
18	4r	368	361	576	437	9813/208	4817/76	0.49	0.45	0.17	0.14	—
19	4s	370	362	581	441	9815/211	4948/79	0.47	0.44	0.16	0.14	—
20	4t	368	354	574	434	9752/206	5207/80	0.45	0.47	0.18	0.15	—
21	4u	367	360	572	436	9765/205	4842/76	0.44	0.41	0.16	0.12	3001
22	4v	368	360	580	436	9933/212	4842/76	0.46	0.46	0.17	0.12	—
23	4w	338	342	578	414	12 285/240	5085/72	0.49	0.50	0.16	0.11	—
24	4x	368	362	560	440	9317/192	4897/78	0.35	0.48	0.16	0.12	—
25	4y	370	362	593	438	10 164/223	4793/76	0.46	0.36	0.19	0.17	2232

a Excitation measured in the solid state.

b Absorption measured in the solution state.

c Emission measured in both solid and solution states.

d Stokes shift; Δ*ν* = *ν*_abs.max_ − *ν*_emis.max_, Δ*λ* = *λ*_emis.max_ − *λ*_abs.max_.

e CCT = −449*n*^3^ + 3525*n*^2^ − 6823*n* + 5520.33; here *n* = (*x* − *x*_e_)/(*y* − *y*_e_), *x*_e_ = 0.3320 and *y*_e_ = 0.1858.

Organic fluorescent molecules with large (mega) Stokes shifts (≥80 nm) can avoid the cross-relaxation results in efficient emission spectra. This indicates that there is no self-quenching by reabsorption and it makes possible to record the fluorescence spectra even with low cost spectrometer. Further, the organic fluorescent material with mega Stokes shift is the desired characteristic property to eliminate self-absorption or the inner filter effect. Hence, the development of fluorophores with mega Stokes shifts and high brightness is highly essential for optoelectronic applications.^61,62^

**Fig. 2:**
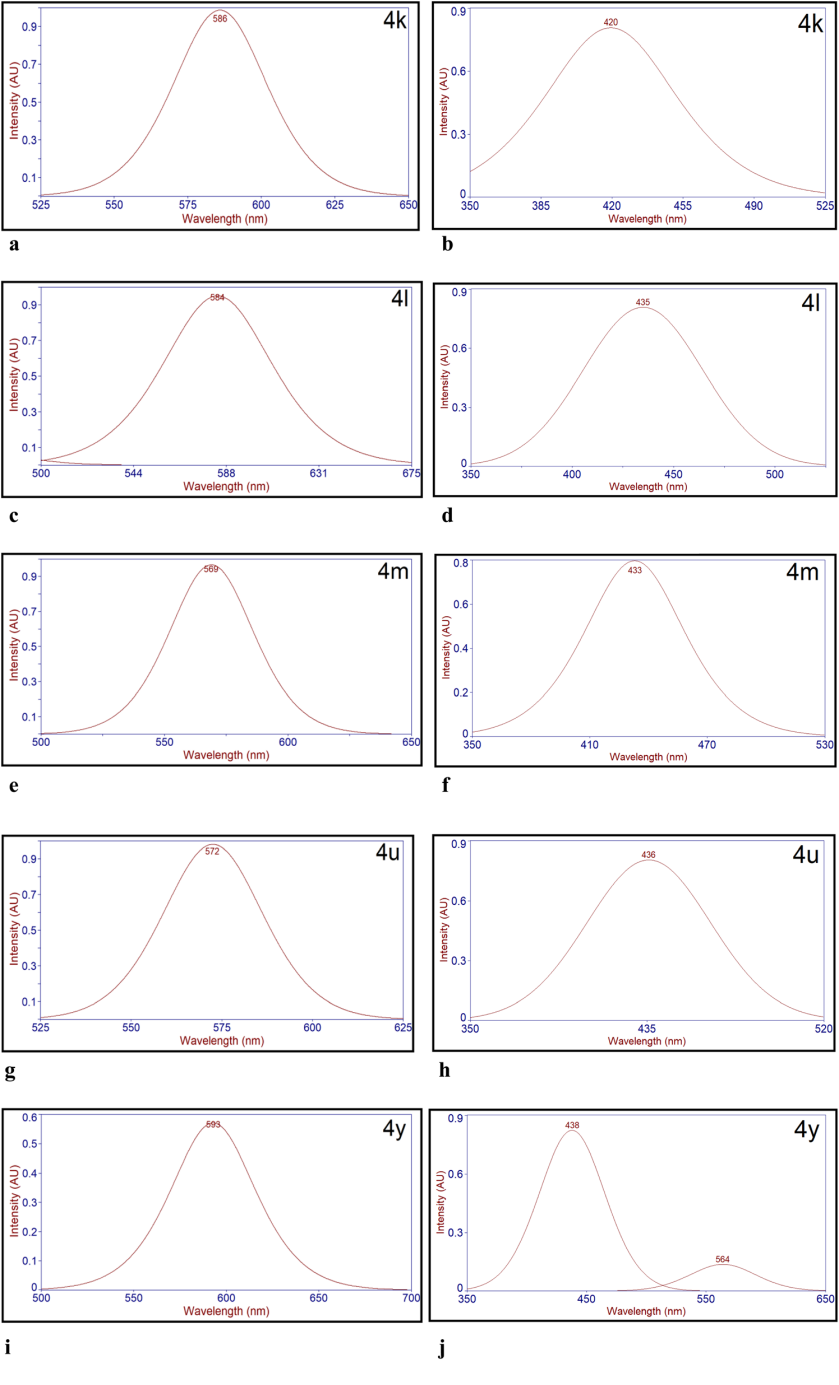
(a) Deconvoluted solid state emission spectrum of 4k. (b) Deconvoluted emission spectrum of 4k in DMSO (1.0 × 10^−5^ M). (c) Deconvoluted solid state emission spectrum of 4l. (d) Deconvoluted emission spectrum of 4l in DMSO (1.0 × 10^−5^ M). (e) Deconvoluted solid state emission spectrum of 4m. (f) Deconvoluted emission spectrum of 4m in DMSO (1.0 × 10^−5^ M). (g) Deconvoluted solid state emission spectrum of 4u. (h) Deconvoluted emission spectrum of 4u in DMSO (1.0 × 10^−5^ M). (i) Deconvoluted solid state emission spectrum of 4y. (j) Deconvoluted emission spectrum of 4y in DMSO (1.0 × 10^−5^ M).

**Fig. 3:**
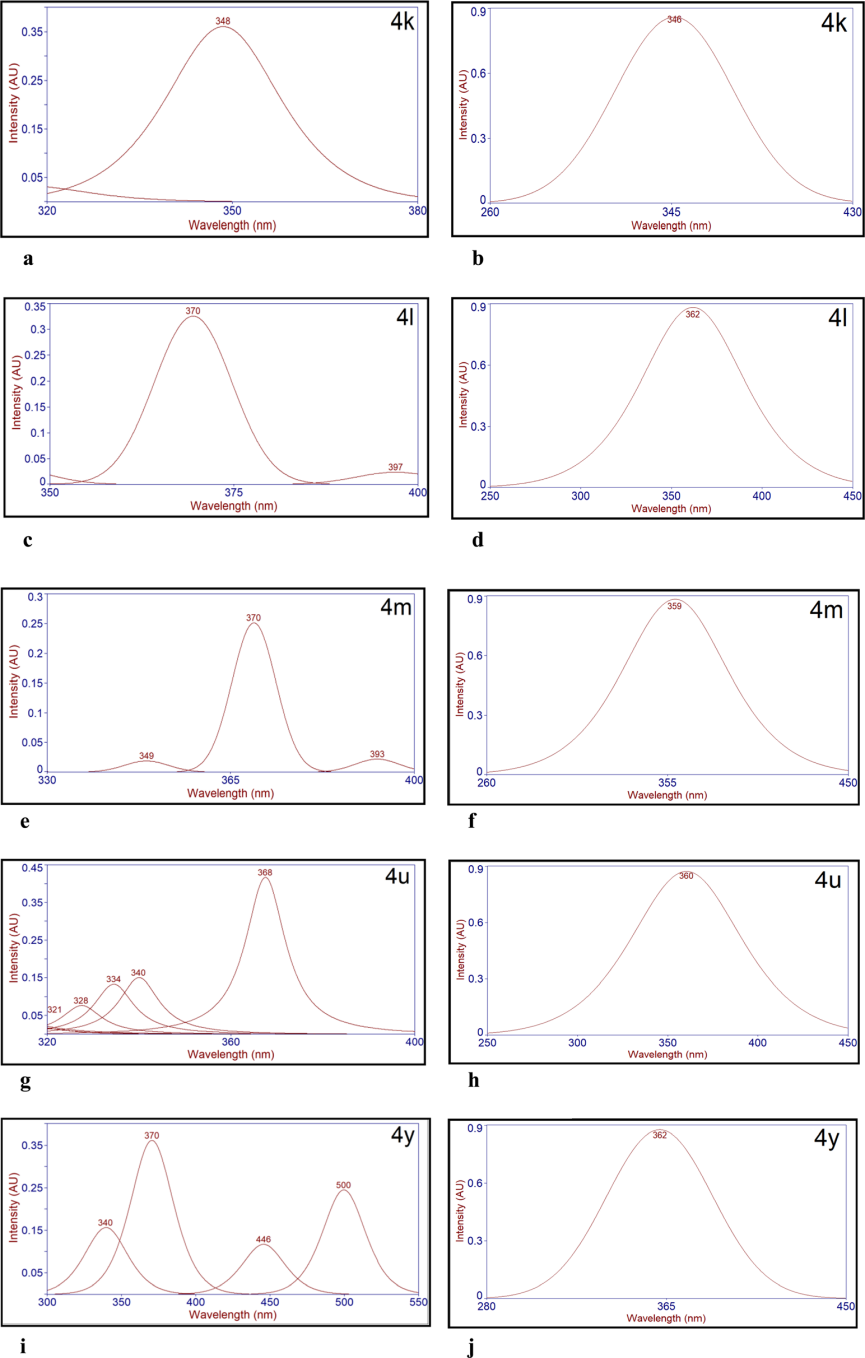
(a) Deconvoluted solid state excitation spectrum of 4k. (b) Deconvoluted absorption spectrum of 4k in DMSO (1.0 × 10^−5^ M). (c) Deconvoluted solid state excitation spectrum of 4l. (d) Deconvoluted absorption spectrum of 4l in DMSO (1.0 × 10^−5^ M). (e) Deconvoluted solid state excitation spectrum of 4m. (f) Deconvoluted absorption spectrum of 4m in DMSO (1.0 × 10^−5^ M). (g) Deconvoluted solid state excitation spectrum of 4u. (h) Deconvoluted absorption spectrum of 4u in DMSO (1.0 × 10^−5^ M). (i) Deconvoluted solid state excitation spectrum of 4y. (j) Deconvoluted absorption spectrum of 4y in DMSO (1.0 × 10^−5^ M).

The Stokes shifts of thiazolylhydrazonoindolin-2-ones (4) have been calculated from the emission and absorption spectra (Fig. 2a–j, 3a–j, S68, S69, S70 and S71†). The results obtained are presented in Table 4. The study reveals that the Stokes shifts are influenced by the substituents on isatin and thiazole rings of the titled compounds (4). Interestingly, all the titled compounds displayed a very large Stokes shifts in the solid state when compared to the solution state. Initially, Stokes shifts of compounds, 4a–4j are influenced by only the substituents at 4^th^ position of thiazole ring. For instance, the electron donating *p*-methoxyphenyl (*p*-OMePh) and *p*-methylphenyl (*p*-MePh) groups at the 4^th^ position of thiazole ring of 4c and 4b were bathochromically shifted with a large Stokes shift as compared to the electron withdrawing *p*-cyanophenyl (*p*-CNPh) group at the same position of thiazole ring of 4h in both solid and solution states (Table 4, entries 2, 3 and 8). The *p*-bromophenyl (*p*-BrPh) group at the 4^th^ position of thiazole ring of 4f was red shifted with a large Stokes shift as compared to the *p*-fluorophenyl (*p*-FPh), *p*-chlorophenyl (*p*-ClPh) and *m*-bromophenyl (*m*-BrPh) groups at the 4^th^ position of thiazole ring of 4d, 4e and 4g, respectively in the solid-state whereas, 4e was red shifted with a large Stokes shift as compared to 4d, 4f and 4g in the solution state (Table 4, entries 4–7).

The phenyl (Ph) group at 4^th^ position of thiazole ring of 4a and β-naphthyl group at 4^th^ position of thiazole ring of 4i were bathochromically shifted with a large Stokes shift as compared to coumarinyl group at the same position of thiazole ring of 4j in the solid-state but, 4a in solution state was blue shifted than 4i and 4j (Table 4, entries 1, 9 and 10). Next, the effect of bromo substituent at 5^th^ position of the isatin and various substituents at 4^th^ position of thiazole rings on Stokes shifts was studied. For example, electron donating *p*-methylphenyl (*p*-MePh) at 4^th^ position of thiazole ring of 4l in the solid-state was red shifted with a large Stokes shift and the same was red shifted with a small Stokes shift in solution state when compared to the *p*-fluorophenyl (*p*-FPh) and *m*-bromophenyl (*m*-BrPh) groups at the 4^th^ position of thiazole ring of 4m and 4n, respectively (Table 4, entries 12–14). Interestingly, compound 4n showed a small Stokes shift as compared with other derivatives in both solid and solution states. This might be due to the presence of bromo substituent on both isatin and phenyl ring of thiazole. The phenyl (Ph) group at 4^th^ position of thiazole ring of 4k was blue shifted with a large Stokes shift and was also bathochromically shifted with a mega Stokes shift as compared to the β-naphthyl and coumarinyl group at the same position of thiazole ring of 4o and 4p, respectively in the solid-state whereas 4k was blue shifted as compared to 4o and 4p with a small and large Stokes shifts, respectively (Table 4, entries 11, 15 and 16). Further, the effect of fluoro substituent at 5^th^ position of the isatin and a variety of substituents at 4^th^ position of thiazole rings on Stokes shifts was also investigated. For instance, the electron donating *p*-methylphenyl (*p*-MePh) and *p*-methoxyphenyl (*p*-OMePh) groups at 4^th^ position of thiazole ring of 4r and 4s were bathochromically shifted with a large Stokes shift as compared to the electron withdrawing *p*-cyanophenyl (*p*-CNPh) group at the same position of thiazole ring of 4x in the solid-state but 4r was blue shifted with a small Stokes shift and 4s was red shifted with a large Stokes shift than 4x, respectively in the solution state (Table 4, entries 18, 19 and 24). The *m*-bromophenyl (*m*-BrPh) group at the 4^th^ position of thiazole ring of 4w was slightly red shifted with a mega Stokes shift as compared to the *p*-fluorophenyl (*p*-FPh) and *p*-chlorophenyl (*p*-ClPh) groups at the 4^th^ position of thiazole ring of 4t & 4u and was slightly blue shifted with a mega Stokes shift when compared to the *p*-bromophenyl (*p*-BrPh) group at the same position of thiazole ring of 4v in the solid-state. Whereas in the solution state, 4t was red shifted with a large Stokes shift as compared to 4w and was slightly blue-shifted with a large Stokes shift than 4u and 4v, respectively (Table 4, entries 20–23). The β-naphthyl group at 4^th^ position of thiazole ring of 4y was bathochromically shifted with a large Stokes shift as compared to the phenyl (Ph) group at the same position of thiazole ring of 4q in the solid state but 4y was blue shifted with a small Stokes shift as compared to 4q in the solution state (Table 4, entries 17 and 25). To our delight, the compounds, 4k, 4l, 4m, 4u and 4y in the solid state exhibited mega Stokes shifts 11 671 cm^−1^ (238 nm), 9904 cm^−1^ (214 nm), 9452 cm^−1^ (199 nm), 9765 cm^−1^ (205 nm) and 10 164 cm^−1^ (223 nm), respectively. From this study, it was clearly stated that there is no self-quenching by reabsorption.

### Optimization of white light emission: CIE colour coordinates and CCT values

To optimize the white light emission in the isatin–thiazole based fluorophores (4), a variety of groups/substituents have been introduced on 4^th^ position of thiazole and 5^th^ position of isatin ring systems.

**Fig. 4:**
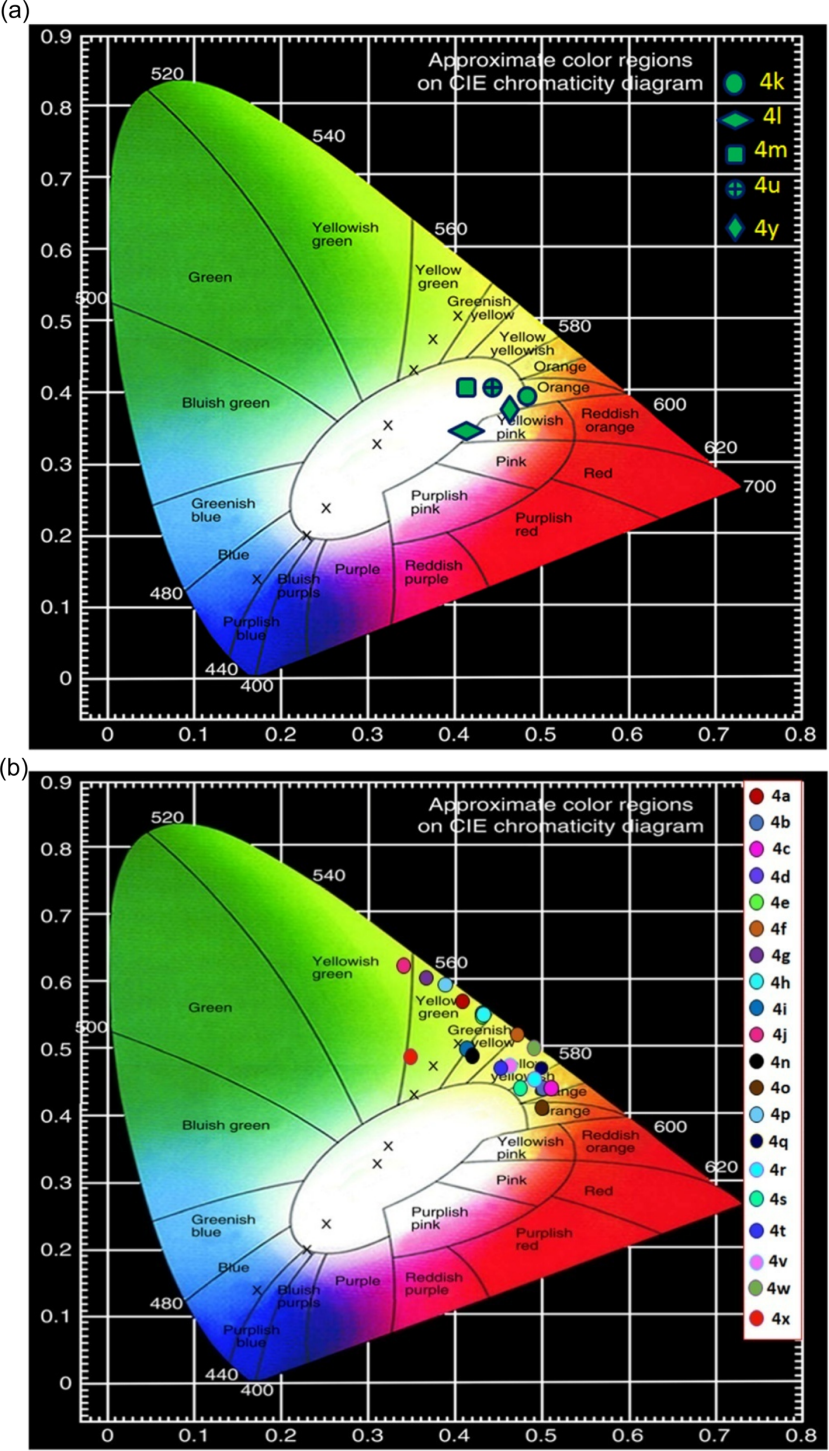
(a) CIE chromaticity diagram of solid state white light emissive 4k, 4l, 4m, 4u and 4y. (b) CIE chromaticity diagram of other thiazolylhydrazonoindolin-2-ones (4a–4j, 4n–4t and 4v–4x) in the solid state.

The CIE colour coordinates (*x*, *y*) of thiazolylhydrazonoindolin-2-ones (4) have been evaluated from the emission spectra (Fig. 2a–j, S68 and S70†) at different excitation wavelengths by adopting the reported procedure.^63^ The obtained results are summarized in Table 4. The colour coordinates of multi-colour emissive fluorophores (4) are shown in Fig. 4a, b and S72.† From CIE colour coordinates (*x*, *y*), it was observed that the colour tunability of 4 mainly depends on both the substituents present on isatin and the substituents at the 4^th^ position of thiazole ring of 4. At first, CIE colour coordinates have been calculated for the compounds, 4a–4j which were prepared from simple isatin (1a), thiosemicarbazide (2) and various α-bromoketones (3a–3j). From this study, it was found that the 4a (Ph), 4d (*p*-FPh), 4e (*p*-ClPh) and 4h (*p*-CNPh) emitted yellowish green light emission excited at 368 nm in the solid state (Table 4, entries 1, 4, 5 and 8). The compounds, 4b (*p*-MePh; *λ*_exi_ = 368 nm) and 4c (*p*-OMePh; *λ*_exi_ = 370 nm) showed yellowish orange light emission (Table 4, entries 2 and 3). The fluorophores, 4f (*p*-BrPh; *λ*_exi_ = 368 nm) and 4i (β-naphthyl; *λ*_exi_ = 370 nm) exhibited greenish yellow light emission (Table 4, entries 6 and 9). The compounds, 4g (*m*-BrPh; *λ*_exi_ = 370 nm) and 4j (coumarinyl; *λ*_exi_ = 371 nm) displayed yellowish green light emission (Table 4, entries 7 and 10). Whereas in the solution state, 4a (Ph; *λ*_exi_ = 352 nm), 4d (*p*-FPh; *λ*_exi_ = 354 nm), 4e (*p*-ClPh; *λ*_exi_ = 352 nm), 4g (*m*-BrPh; *λ*_exi_ = 352 nm), 4h (*p*-CNPh; *λ*_exi_ = 357 nm), 4i (β-naphthyl; *λ*_exi_ = 362 nm) and 4j (coumarinyl; *λ*_exi_ = 361 nm) exhibited blue light emission (Table 4, entries 1, 4, 5, 7–10). The compounds, 4b (*p*-MePh; *λ*_exi_ = 350 nm) and 4f (*p*-BrPh; *λ*_exi_ = 352 nm) showed greenish blue light emission in the solution state (Table 4, entries 2 and 6). The compound 4c (*p*-OMePh; *λ*_exi_ = 369 nm) displayed yellowish green light emission in the solution state (Table 4, entry 3). Further, CIE colour coordinates have been calculated for the compounds (4k–4p) which were synthesized from 5-bromoisatin (1b), thiosemicarbazide (2) and various α-bromoketones (3a, 3b, 3d, 3g, 3i and 3j). The study revealed that the compounds 4k (Ph; *λ*_exi_ = 348 nm), 4l (*p*-MePh; *λ*_exi_ = 370 nm) and 4m (*p*-FPh; *λ*_exi_ = 370 nm) exhibited warm white light emission (Table 4, entries 11–13) whereas 4n (*m*-BrPh) displayed greenish yellow light emission at *λ*_exi_ = 503 nm (Table 4, entry 14), 4o (β-naphthyl) emitted orange light emission at *λ*_exi_ = 372 nm (Table 4, entry 15) and 4p (coumarinyl) exhibited yellow green light emission at *λ*_exi_ = 370 nm (Table 4, entry 16) in the solid state. On the other hand, the compounds 4k (Ph; *λ*_exi_ = 346 nm), 4l (*p*-MePh; *λ*_exi_ = 362 nm), 4m (*p*-FPh; *λ*_exi_ = 359 nm) and 4n (*m*-BrPh; *λ*_exi_ = 352 nm) showed purplish blue light emission in the solution state (Table 4, entries 11–14). The compounds 4o (β-naphthyl; *λ*_exi_ = 397 nm) and 4p (4-coumarinyl; *λ*_exi_ = 362 nm) exhibited yellowish green light emission in the solution state (Table 4, entries 15 and 16). Next, CIE colour coordinates have been calculated for the compounds (4q–4y) which were synthesized from 5-fluoroisatin (1c), thiosemicarbazide (2) and various α-bromoketones (3a–3i). From this investigation, it was observed that the compounds 4q (Ph; *λ*_exi_ = 370 nm), 4s (*p*-OMePh; *λ*_exi_ = 370 nm), 4t (*p*-FPh; *λ*_exi_ = 368 nm), 4v (*p*-BrPh; *λ*_exi_ = 368 nm), 4w (*m*-BrPh; *λ*_exi_ = 338 nm), 4x (*p*-CNPh; *λ*_exi_ = 368 nm) displayed yellow light emission (Table 4, entries 17, 19, 20, 22, 23 and 24) whereas 4r (*p*-MePh) showed orange light emission at *λ*_exi_ = 368 nm (Table 4, entry 18). Interestingly, the compounds, 4u (*p*-ClPh; *λ*_exi_ = 368 nm) and 4y (β-naphthyl; *λ*_exi_ = 370 nm) emitted warm white light (Table 4, entries 21 and 25) in the solid state. Whereas in the solution state, the compounds 4q (Ph; *λ*_exi_ = 362 nm), 4r (*p*-MePh; *λ*_exi_ = 361 nm), 4s (*p*-OMePh; *λ*_exi_ = 362 nm), 4t (*p*-FPh; *λ*_exi_ = 354 nm), 4u (*p*-ClPh; *λ*_exi_ = 360 nm), 4x (*p*-CNPh; *λ*_exi_ = 362 nm) and 4y (β-naphthyl; *λ*_exi_ = 362 nm) emitted blue light emission (Table 4, entries 17–21, 24 and 25). The compounds 4v (*p*-BrPh; *λ*_exi_ = 360 nm) and 4w (*m*-BrPh; *λ*_exi_ = 342 nm) were showed purplish blue light emission in the solution state (Table 4, entries 22 and 23). Interestingly, the compounds, 4k, 4l, 4m, 4u and 4y exhibited warm white light emission in the solid state and this results were supported by the correlated colour temperature (CCT) analysis.

The CCT values are calculated by using the McCamy empirical formula [ref. 64, eqn (1)] to characterize the colour emission and its temperature.

1 CCT = −449*n*^3^ + 3525*n*^2^ − 6823*n* + 5520.33

where *n* = (*x* − *x*_e_)/(*y* − *y*_e_) and the chromaticity epicenter is at *x*_e_ = 0.3320 and *y*_e_ = 0.1858.

The CCT values of titled compounds (4) were calculated that ranging from 2225 K to 3554 K (Table 4). To our delight, the titled compounds, 4k (2225 K) (orange warm white light), 4l (2913 K) (warm white light), 4m (3554 K) (warm white light), 4u (3001 K) (warm white light) & 4y (2232 K) (warm white light) were emitted warm white light. It is worthy to mention that the electrochemical behaviour of the synthesized fluorophores has already been reported as ambipolar and hole-transporting materials (HTMs) for optoelectronic applications.^57^ From the photophysical and electrochemical properties, it could be concluded that the white light emissive compounds 4k, 4l, 4m, 4u and 4y can act as both hole-transport and ambipolar materials, hence, the cost and size of WOLED devices can be reduced.

The quantum yields (QYs) of white light emissive titled compounds, 4k, 4l, 4m, 4u and 4y in the solid state have been determined by using CREE-make YAG:Ce^3+^ as a standard reference. The QYs of the reference and the abovementioned compounds (4k, 4l, 4m, 4u and 4y) were measured as dispersed powders on quartz slides to simulate their usage in white LED applications. Identical conditions were maintained to ensure consistency. The QYs (%) of white light emissive compounds, 4k, 4l, 4m, 4u and 4y were found to be 0.56, 0.21, 0.32, 0.31 and 0.21, respectively, in the solid state.

## Experimental section

### General experimental information

All chemicals and solvents were purchased from commercial sources (Sigma-Aldrich, Acros Organics Ltd., Avra, and Merck) and were used as received. α-Bromoketones (3) were prepared as per the reported procedure in the literature.^65^ The progress of the reactions was monitored by TLC using 0.25 mm Merck silica gel plates and the spots were visualized under UV light. The ^1^H-NMR spectra were recorded on a Bruker NMR spectrometer (400 MHz). Mass spectra were recorded on Shimadzu-LCMS-2010A mass spectrometer. The absorbance spectra were taken using a Shimadzu model UV-3100 UV-visible spectrophotometer. Emission and excitation spectra were recorded on Hitachi fluorescence spectrophotometer (F-2710). Deconvolution analysis of absorption and emission spectral data were carried out by using PeakFit 4.12 version.

### Typical experimental procedure for synthesis of thiazolylhydrazonoindolin-2-ones (4)

A mixture of isatins (1) (5.0 mmol), thiosemicarbazide (2) (5.0 mmol), α-bromoketones (3) (5.0 mmol), 0.1 N citric acid (5.0 mL) and ethanol (3.0 mL) was stirred at reflux conditions for 40–60 min. The reaction progress was monitored by TLC. Upon completion of the reaction, the mixture was cooled to room temperature and the obtained crude product (4) was extracted twice with ethyl acetate (2 × 5 mL). The resulting organic layer was dried over anhydrous MgSO_4_, volatiles were removed and the obtained product (4) was recrystallized from ethanol.

### Scale-up procedure for the synthesis of 3-(2-(4-(4-bromophenyl)thiazol-2-yl)hydrazono)indolin-2-one (4f)

A mixture of isatin (1) (0.0362 mol), thiosemicarbazide (2) (0.0362 mol), 2,4′-dibromoacetophenone (3f) (0.0362 mol), 0.1 N citric acid (30.0 mL) and ethanol (20.0 mL) was stirred at reflux conditions for 60 min. The reaction progress was monitored by TLC. Upon completion of the reaction, the mixture was cooled to room temperature and the obtained crude product (4) was extracted twice with ethyl acetate (2 × 25 mL). The resulting organic layer was dried over anhydrous MgSO_4_, volatiles were removed and the obtained product (4f) was recrystallized from ethanol.

### Procedure adapted for quantum yield measurements

The standard reference, cerium-doped yttrium aluminum garnet (YAG:Ce^3+^) and the white light emissive samples (4k, 4l, 4m, 4u and 4y) were finely ground and uniformly coated on quartz slides. The layer thickness was optimized to ensure low optical density (*A* < 0.1) at the excitation wavelength. An excitation wavelength (*λ*_exi_) of 460 nm was chosen which was corresponding to the blue LED emission commonly used in white LED fabrication. The emission spectra of the reference and the samples were recorded under identical conditions using the integrating sphere. The integration time, slit widths, and detector gain were kept constant. The absorbance spectra at *λ*_exi_ = 450 nm was measured for both the reference and the samples (4k, 4l, 4m, 4u and 4y). The fraction of light absorbed (*F*_abs,ref_ & *F*_abs,samples_) and integrated emission intensities (*I*_ref_ and *I*_samples_) obtained from the area under the respective emission spectrum of both reference and the samples were calculated to obtain QYs (*Φ*_samples_) using the following formula:
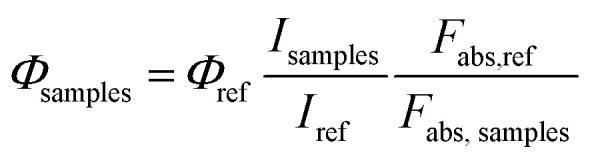
where, *Φ*_ref_ = 0.76 (QY of the YAG:Ce^3+^ reference).

*I*_samples_ and *I*_ref_: integrated emission intensities of the samples (4k, 4l, 4m, 4u and 4y) and reference, respectively.

*F*_abs,samples_ and *F*_abs,ref_: fractions of light absorbed by the samples (4k, 4l, 4m, 4u and 4y) and reference, respectively.

Since the measurements were performed in the solid state configuration, the refractive index (*η*) was assumed to be approximately equal for both the samples, simplifying the calculation by eliminating the refractive index correction term.

## Conclusion

Biodegradable citric acid catalyzed 3-component reaction strategy has proved to be a promising synthetic route to develop multi-colour emissive isatin–thiazole based fluorophores (4) from readily available isatins (1), thiosemicarbazide (2) and α-bromoketones (3). This reaction proceeds *via* condensation (C=N) and subsequent thiazole formation (C–S & C–N) in a single step operation. This MCR strategy is highlighted by step economy, cleaner reaction profile, simple to perform, broad substrate scope, use of non-toxic solvents/catalysts, good functional group tolerance, gram scale feasibility, column-free purification, high product yields (91–98%) in a shorter reaction times *etc.* Gratifyingly, the synthesized isatin–thiazole based luminophores (4) displayed substituent-dependent tunable optical properties in both solid and solution states like mega Stoke shifts and multi-colour emissions. Thus, the developed multi-colour emissive isatin–thiazole based molecular hybrids could be employed as promising candidates for the fabrication of organic opto-electronic devices. Further, it is worthy to conclude that the compounds, 4k, 4l, 4m, 4u and 4y emitted white light in the solid state with mega Stokes shifts.

## Data availability

The NMR and mass spectral data supporting this article have been included as part of the ESI.†

## Conflicts of interest

The authors declare that they have no known competing financial interests or personal relationships that could have appeared to influence the work reported in this paper.

## Supplementary Material

RA-015-D4RA09010A-s001
