# Supplementary material for: Optimizing the white light emission in the solid state isatin and thiazole based molecular hybrids by introduction of variety of substituents on isatin and thiazole ring systems
Source: RSC Adv. 2025 Mar 14;15(10):7973–86. doi: 10.1039/d4ra09010a (PMC11908639; doi:10.1039/d4ra09010a)
Supplement: RA-015-D4RA09010A-s001 [file RA-015-D4RA09010A-s001.pdf]

Supplementary Information (SI) for RSC Advances.
This journal is © The Royal Society of Chemistry 2025

Optimizing the white light emission in the solid state isatin and thiazole based molecular hybrids by introduction of variety of substituents on isatin and thiazole ring systems

Sultana Shaik,[1#] Rama Mohana Reddy Sirigireddy,[1#] Sai Teja Talari,[1#] Haranath Divi,[3] Naveen Mulakayala,[4] Venkatramu Vemula,[2]* and Chinna Gangi Reddy Nallagondu[1]*

[1] *Green & Sustainable Synthetic Organic Chemistry and Optoelectronics Laboratory, Department of Chemistry, Yogi Vemana University, Kadapa - 516005, Andhra Pradesh, India.*

[2] *Department of Physics, Yogi Vemana University, Kadapa-516 005, Andhra Pradesh, India.*

[3] *Department of Physics, National Institute of Technology, Warangal- 506004, Telangana, India*

SVAK Lifesciences, ALEAP Industrial Area, Pragathi Nagar, Hyderabad 500090, India.

**Corresponding authors*: ncgreddy@yogivemanauniversity.ac.in;ncgreddy@yvu.edu.in (Chinna Gangi Reddy Nallagondu), vvramuphd@gmail.com (Venkatramu Vemula)

[#] Equal contributors

Table of Contents

	Page No.
Physical and spectral data (^{1}H-NMR and Mass) of thiazolylhydrazonoindolin-2-ones (**4**)	3-8
Copies of ^{1}H NMR and Mass spectra of thiazolylhydrazonoindolin-2-ones (**4**)	9-75
Deconvoluted solid-state emission spectra of thiazolylhydrazonoindolin-2-ones (**4**)	76-77
Deconvoluted solid-state excitation spectra of thiazolylhydrazonoindolin-2-ones (**4**)	78-79
Deconvoluted emission spectra of **4a-4j, 4n-4t** & **4v-4x** in DMSO ($1.0x10^{-5}$ M)	80-81
Deconvoluted absorption spectra of **4a-4j, 4n-4t** & **4v-4x** in DMSO ($1.0x10^{-5}$ M)	82-83
CIE chromaticity diagram of thiazolylhydrazonoindolin-2-ones (**4**) in the solution state.	84

Physical andspectral data(^{1}H-NMR and Mass) of thiazolylhydrazonoindolin-2-ones (4)

**(*Z*)-3-(2-(4-phenylthiazol-2-yl)hydrazono)indolin-2-one (4a):** 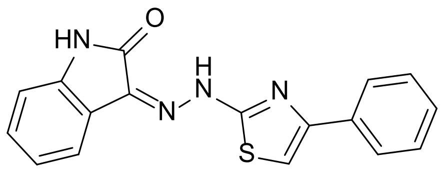	mp: 272-273°C.^{1}H NMR (400MHz, d_6-DMSO): δ 13.36 (s, 1H, -NH), 11.27 (s, 1H, NH of lactam), 7.91 (d, *J*=7.2 Hz, 2H, arom H), 7.64 (s, 1H, Thiazole H), 7.55 (d, *J*=7.2 Hz, 1H, arom H), 7.43 (t, *J*=7.6 Hz, 2H, arom H), 7.37-7.32 (m, 2H, arom H), 7.10 (t, *J*=7.6 Hz, 1H, arom H), 6.97 (d, *J*=8.0 Hz, 1H, arom H). MS (ESI): [M+H]$^+$ 321.37.
**(Z)-3-(2-(4-(p-tolyl)thiazol-2-yl)hydrazono)indolin-2-one (4b)** 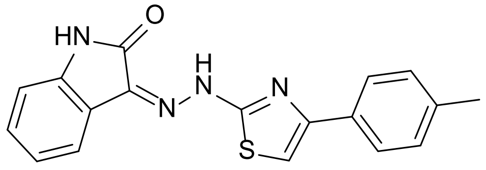	mp: 281-283°C.^{1}H NMR (400MHz, d_6-DMSO): δ 13.34 (s, 1H, -NH), 11.25 (s, 1H, NH of lactam), 7.79 (d, *J*=8.4 Hz, 2H, arom H), 7.55-7.53 (m, 2H, Thiazole **H**and1H, arom H),7.34 (td, *J*=7.6 Hz, *J* =1.2 Hz, 1H,arom H), 7.23 (d, *J*=8.0 Hz, 2H, arom H), 7.09 (td, *J*=7.6 Hz, *J*=0.8 Hz, 1H, arom H), 6.97 (d, *J* =7.6 Hz, 1H,arom H), 2.33 (s, 3H, $-CH_3$).MS (ESI): [M+H]$^+$ 335.33.
**(*Z*)-3-(2-(4-(4-methoxyphenyl)thiazol-2-yl)hydrazono)indolin-2-one (4c):** 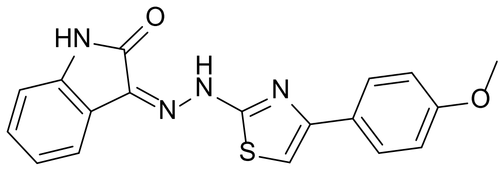	mp: 269-271°C.^{1}H NMR (400MHz, d_6-DMSO): δ 13.34 (s, 1H, -NH), 11.26 (s, 1H, NH of lactam), 7.84 (d, *J*=9.6 Hz, 2H, arom H), 7.54 (d, *J*=7.2 Hz, 1H, arom H), 7.47 (s, 1H, Thiazole **H**), 7.35 (td, *J*=8.0 Hz, *J*=1.2 Hz, 1H, arom H), 7.10 (t, *J* =7.4 Hz, 1H, arom H), 7.00-6.96 (m, 3H, arom H), 3.79 (s, 3H, $-OCH_3$). MS (ESI): [M+H]$^+$ 351.38.
**(*Z*)-3-(2-(4-(4-fluorophenyl)thiazol-2-yl)hydrazono)indolin-2-one (4d)** 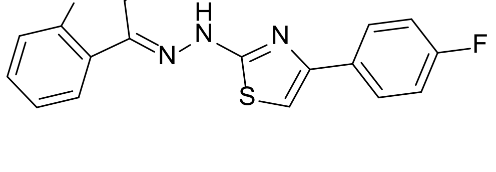	mp: 260-261°C. ^{1}H NMR (400MHz, d_6-DMSO): δ 13.35 (s, 1H, -NH), 11.26 (s, 1H, NH of lactam), 7.97-7.93 (m, 2H, arom H), 7.62 (s, 1H, Thiazole **H**), 7.55 (d, *J*=7.6 Hz, 1H, arom H), 7.35 (td, *J*=7.6 Hz, *J*=1.2 Hz, 1H, arom H), 7.26 (t, *J*=9.0 Hz, 2H, arom H), 7.12-7.07(m, 1H, arom H), 6.97 (d, *J*=7.6 Hz, 1H,arom H). MS (ESI): [M+H]$^+$ 339.30

**(*Z*)-3-(2-(4-(4-chlorophenyl)thiazol-2-yl)hydrazono)indolin-2-one (4e)** 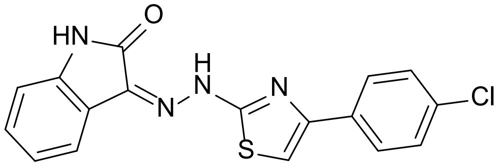	mp: 291-294 °C.^{1}H NMR (400MHz, d_6-DMSO): δ 13.36 (s, 1H, -NH), 11.26 (s, 1H, NH of lactam), 7.93 (d, *J*=8.8 Hz, 2H,arom H), 7.70 (s, 1H, Thiazole **H**), 7.55 (d, *J*=7.6 Hz, 1H, arom H), 7.49 (d, *J*=8.8 Hz, 2H,arom H), 7.35 (td, *J*=7.6 Hz, *J*=1.2 Hz, 1H, arom H),7.01 (td, *J*=7.6 Hz, *J*=0.8 Hz, 1H, arom H),6.97 (d, *J*=7.6 Hz, 1H,arom H). MS (ESI): $[M+H]^+$ 355.25; $[M+H+2]^+$ 357.25.
**(*Z*)-3-(2-(4-(4-bromophenyl)thiazol-2-yl)hydrazono)indolin-2-one (4f):** 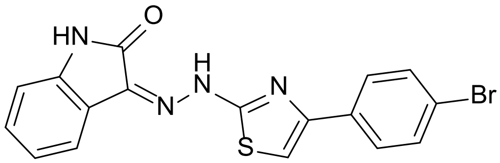	mp: 250-251°C.^{1}H NMR (400MHz, d_6-DMSO): δ 13.36 (s, 1H, -NH), 11.27 (s, 1H, NH of lactam), 7.86 (d, *J*=8.8 Hz, 2H, arom H), 7.72 (s, 1H, Thiazole **H**), 7.63 (d, *J*=8.8 Hz, 2H, arom H), 7.55 (d, *J*=7.2 Hz, 1H, arom H), 7.35 (td, *J*=7.8 Hz, *J*=1.2 Hz, 1H, arom H), 7.10 (t, *J*=7.2 Hz, 1H, arom H), 6.97 (d, *J*=8.0 Hz, 1H, arom H). MS (ESI): $[M+H]^+$399.28; $[M+H+2]^+$ 401.28.
**(*Z*)-3-(2-(4-(3-bromophenyl)thiazol-2-yl)hydrazono)indolin-2-one (4g):** 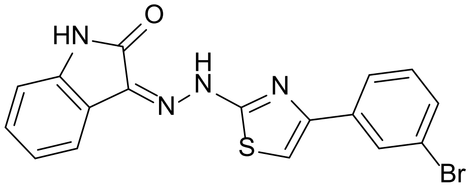	mp: 245-246°C.^{1}H NMR (400MHz, d_6-DMSO): δ 13.35 (s, 1H, -NH), 11.28 (s, 1H, NH of lactam), 8.09 (t, *J*=1.8 Hz, 1H, arom H), 7.92 (dt, *J*=7.6 Hz, *J*=1.2 Hz, 1H, arom H), 7.79 (s, 1H, Thiazole **H**), 7.56-7.52 (m, 2H, arom H), 7.40 (t, *J*=7.8 Hz, 1H, arom H), 7.35 (td, *J*=7.6 Hz, *J*=1.2 Hz, 1H, arom H), 7.10 (td, *J*=7.6 Hz, *J*=0.8 Hz, 1H, arom H), 6.97 (d, *J*=7.6 Hz, 1H, arom H). MS (ESI): $[M+H]^+$399; $[M+H+2]^+$ 401.23.
**(*Z*)-4-(2-(2-(2-oxoindolin-3-ylidene)hydrazinyl)thiazol-4-yl)benzonitrile (4h):**	mp: 268-269°C.^{1}H NMR (400MHz, d_6-DMSO): δ 13.36 (s, 1H, -NH), 11.27 (s, 1H, NH of lactam), 7.86 (d, *J*=9.2 Hz, 2H, arom H), 7.72 (s, 1H, Thiazole **H**), 7.63 (d, *J*=9.2 Hz, 2H, arom H), 7.55 (d, *J*=7.2 Hz, 1H,arom H), 7.35 (td, *J*=7.6 Hz, *J*=1.2 Hz, 1H,arom H), 7.10 (t, *J*=7.2 Hz, 1H, arom H), 6.97 (d,

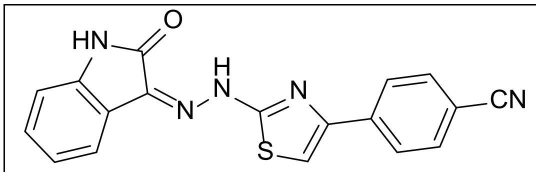	*J*=8.0 Hz, 1H, arom H). MS (ESI):[M+H]$^+$346.36.
**(*Z*)-3-(2-(4-(naphthalen-2-yl)thiazol-2-yl)hydrazono)indolin-2-one (4i)** 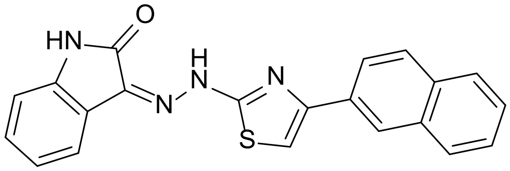	mp: 286-288°C.^{1}H NMR (400MHz, d_6-DMSO): δ 13.42 (s, 1H, -NH), 11.27 (s, 1H, NH of lactam), 8.48 (s, 1H, arom H), 8.06-7.91 (m, 4H arom H), 7.79 (s, 1H, Thiazole **H**), 7.57 (d, *J*=7.2 Hz, 1H,arom H), 7.54-7.51 (m, 2H, arom H), 7.36 (td, *J*=7.6 Hz, *J*=1.2 Hz, 1H, arom H), 7.11 (t, *J*=7.6 Hz, 1H, arom H), 6.98 (d, *J*=7.6 Hz, 1H,arom H).MS (ESI):[M+H]$^+$371.31.
**(*Z*)-5-bromo-3-(2-(4-phenylthiazol-2-yl)hydrazono)indolin-2-one (4k)** 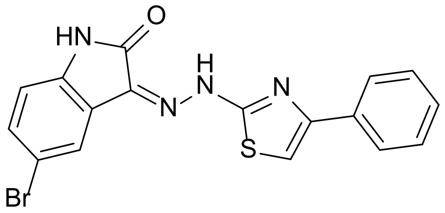	mp: 286-288°C.^{1}H NMR (400MHz, d_6-DMSO): δ 13.33 (s, 1H, -NH), 11.37 (s, 1H, NH of lactam), 7.91 (d, *J*=7.2 Hz, 2H, arom H), 7.68 (s, 1H, Thiazole **H**), 7.65 (d, *J*=2.0 Hz, 1H, arom H), 7.51 (dd, *J*=8.4 Hz, *J*=2.0 Hz, 1H, arom H), 7.43 (t, *J*=7.4 Hz, 2H, arom H), 7.34 (tt, *J*=7.2 Hz, *J*=1.2 Hz, 1H, arom H), 6.94 (d, *J*=8.4 Hz, 1H, arom H).MS (ESI): [M+H]$^+$ 399.16, [M+H+2]$^+$ 401.
**(*Z*)-5-bromo-3-(2-(4-(p-tolyl)thiazol-2-yl)hydrazono)indolin-2-one (4l)** 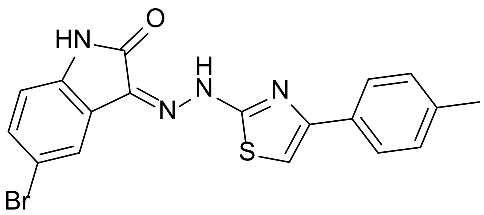	mp: 301-302°C.^{1}H NMR (400MHz, d_6-DMSO): δ 13.32 (s, 1H, -NH), 11.37 (s, 1H, NH of lactam), 7.79 (d, *J*=8.0 Hz, 2H,arom H), 7.65 (d, *J*=1.6 Hz, 1H,arom H), 7.60 (s, 1H, Thiazole **H**), 7.51 (dd, *J*=8.2 Hz, *J*=1.8 Hz, 1H,arom H), 7.24 (d, *J*=8.0 Hz, 2H,arom H), 6.94 (d, *J*=8.4 Hz, 1H,arom H), 2.33 (s, 1H, -CH$_3$). MS (ESI): [M+H]$^+$413, [M+H+2]$^+$ 415.18.
**(*Z*)-5-bromo-3-(2-(4-(4-fluorophenyl)thiazol-2-**	mp: 296-297°C.^{1}H NMR (400 MHz, d_6-DMSO): δ 13.32 (s, 1H, -NH), 11.37 (s, 1H, NH of lactam), 7.97-

**yl)hydrazono)indolin-2-one(4m)** 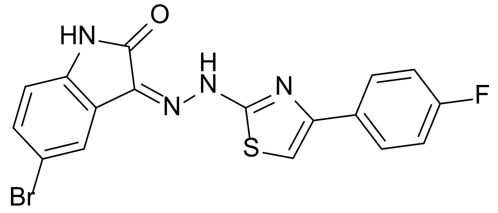	7.93 (m, 2H, arom H), 7.66-7.65 (m, 2H, 1H, Thiazole**H,** 1H,arom Hand **H**), 7.51 (dd, *J*=8.4 Hz, *J*=2.0 Hz 1H, arom H), 7.27 (t, *J*=9.0 Hz,2H, arom H), 6.93 (d, *J*=8.4 Hz,1H, arom H). MS (ESI): $[M+H]^+$417, $[M+H+2]^+$ 419.18.
**5-bromo-3-(2-(4-(naphthalen-2-yl)thiazol-2-yl)hydrazono)indolin-2-one (4o):** 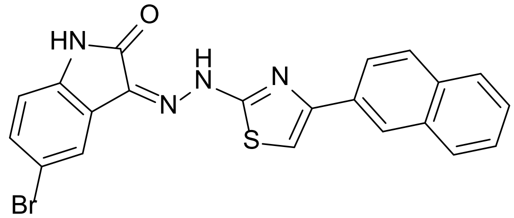	mp: 292-293°C. ^{1}H NMR (400MHz, d_6-DMSO): δ 13.40 (s, 1H, -NH), 11.39 (s, 1H, NH of lactam), 8.48 (s, 1H, arom H), 8.06-7.91(m, 4H, arom H), 7.83 (s, 1H, Thiazole **H**), 7.67 (d, *J*=2.0 Hz, 1H, arom H), 7.56-7.50 (m, 3H, arom H), 6.95 (d, *J*=8.4 Hz, 1H,arom H). MS (ESI): $[M+H]^+$449, $[M+H+2]^+$ 451.23.
**(Z)-5-fluoro-3-(2-(4-phenylthiazol-2-yl)hydrazono)indolin-2-one (4q)** 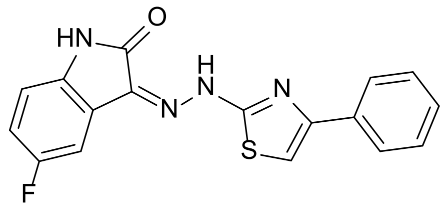	mp: 286-287°C.^{1}H NMR (400MHz, d_6-DMSO): δ 13.40 (s, 1H, -NH), 11.27 (s, 1H, NH of lactam), 7.91 (d, *J*=7.2 Hz, 2H, arom H), 7.67 (s, 1H, Thiazole **H**), 7.44 (t, *J*=7.6 Hz, 2H, arom H), 7.38-7.32 (m, 2H, arom H), 7.21-7.16 (m, 1H, arom H), 6.97 (dd, *J*=8.6 Hz, *J*=4.2 Hz, 1H, arom H). MS (ESI): $[M+H]^+$339.30.
**(*Z*)-5-fluoro-3-(2-(4-(p-tolyl)thiazol-2-yl)hydrazono)indolin-2-one (4r)** 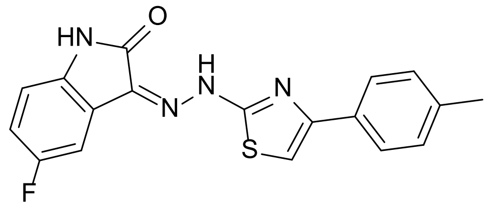	mp: 304-305°C.^{1}H NMR (400MHz, d_6-DMSO): δ 13.38 (s, 1H, -NH), 11.26 (s, 1H, NH of lactam), 7.79 (d, *J*=8.4Hz, 2H,arom H), 7.59 (s, 1H, Thiazole **H**), 7.36 (dd, *J*=8.2 Hz, *J*=2.6 Hz, 1H,arom H), 7.23 (d, *J*=7.6 Hz, 2H,arom H), 7.21-7.15 (m, 1H,arom H), 6.96 (dd, *J*=8.6 Hz, *J*=4.2 Hz, 1H, arom H), 2.33 (s, 3H, -CH_3). MS (ESI): $[M+H]^+$353.34.

**(*Z*)-5-fluoro-3-(2-(4-(4-methoxyphenyl)thiazol-2-yl)hydrazono)indolin-2-one (4s)** 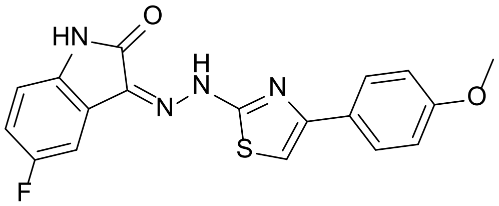	mp: 293-294 °C.^{1}H NMR (400MHz, *d*$_6$-DMSO): δ 13.37 (s, 1H, NH), 11.26 (s, 1H, NH of lactam), 7.83 (d, *J*=8.8 Hz, 2H, arom H), 7.49 (s, 1H, Thiazole **H**), 7.35 (dd, *J*=8.2 Hz, *J*=2.6 Hz, 1H,arom H),7.21-7.15 (m, 1H, arom H), 7.06-6.90 (m, 3H, arom H), 3.79 (s,3H, -OCH_3). MS (ESI): $[M+H]^+$369.27.
**(*Z*)-5-fluoro-3-(2-(4-(4-fluorophenyl) thiazol-2-yl)hydrazono)indolin-2-one (4t)** 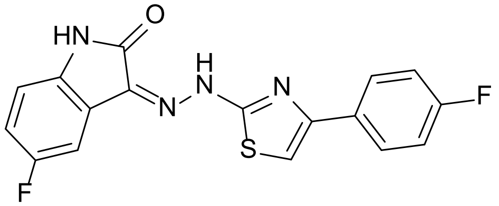	mp: 273-275°C.^{1}H NMR (400 MHz, *d*$_6$-DMSO): δ 13.39 (s, 1H, -NH), 11.27 (s, 1H, NH of lactam), 7.97-7.93 (m, 2H, arom H), 7.65 (s, 1H, Thiazole **H**), 7.36 (dd, *J*=8.0 Hz, *J*=2.4 Hz 1H, arom H), 7.26 (t, *J*=9.0 Hz,2H, arom H), 7.18 (td, *J*=8.8 Hz, *J*=2.6 Hz, 1H, arom H), 6.96 (dd, *J*=8.4 Hz, *J*=4.4 Hz, 1H, arom H). MS (ESI): $[M+H]^+$357.24.
**(*Z*)-3-(2-(4-(4-chlorophenyl)thiazol-2-yl)hydrazono)-5-fluoroindolin-2-one (4u)** 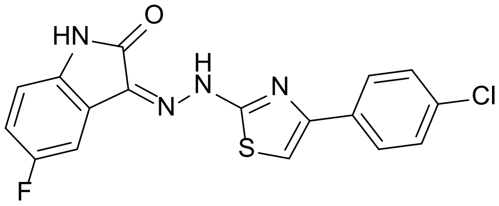	mp: 295-296°C.^{1}H NMR (400 MHz, *d*$_6$-DMSO): δ 13.39 (s, 1H, -NH), 11.27 (s, 1H, NH of lactam), 7.93 (d, *J*=8.4 Hz, 2H, arom H), 7.73 (s, 1H, Thiazole **H**), 7.49 (d, *J*=8.4 Hz,2H, arom H), 7.36 (dd, *J*=8.0 Hz, *J*=2.4 Hz, 1H, arom H), 7.19 (td, *J*=8.4 Hz, *J*=2.8 Hz, 1H, arom H), 6.96 (dd, *J*=8.6 Hz, *J*=4.2 Hz, 1H, arom H). MS (ESI): $[M+H]^+$373.28; $[M+H+2]^+$ 375.27.

**(*Z*)-3-(2-(4-(4-bromophenyl)thiazol-2-yl)hydrazono)-5-fluoroindolin-2-one (4v)**<br>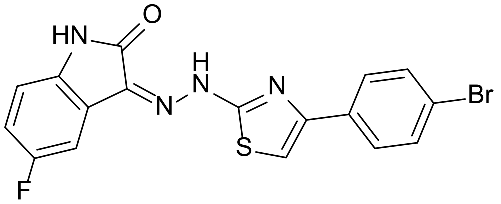	mp: 290-292 °C.^{1}H NMR (400MHz, d_6-DMSO): δ 13.39 (s, 1H, -NH), 11.27 (s, 1H, NH of lactam), 7.86 (d, *J*=8.8 Hz,2H, arom H), 7.74 (s, 1H, Thiazole **H**), 7.62 (d, *J*=8.8, Hz, 2H, arom H), 7.36 (dd, *J*=8.0 Hz, *J*=2.8 Hz, 1H, arom H), 7.18 (td, *J*=8.4 Hz, *J*=2.4 Hz, 1H, arom H), 6.96 (dd, *J*=8.6 Hz, *J*=2.2 Hz, 1H, arom H).
**(*Z*)-4-(2-(2-(5-fluoro-2-oxoindolin-3-ylidene)hydrazinyl)thiazol-4-yl)benzonitrile (4x)**<br>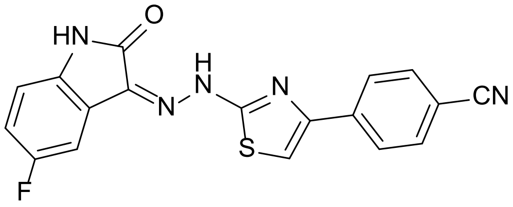	mp: 283-285°C.^{1}H NMR (400MHz, d_6-DMSO): δ 13.40 (s, 1H, -NH), 11.28 (s, 1H, NH of lactam), 8.09 (d, *J*=8.4 Hz, 2H, arom H), 7.96 (s, 1H, Thiazole **H**), 7.89 (d, *J*=8.4 Hz, 2H, arom H), 7.37 (dd, *J*=8.0 Hz, *J*=2.8 Hz, 1H, arom H), 7.19 (td, *J*=9.6 Hz, *J*=2.6 Hz, 1H, arom H), 6.96 (dd, *J*=8.6 Hz, *J*=4.2 Hz, 1H, arom H).MS (ESI): $[M+H]^{+}$364.32.
**(*Z*)-5-fluoro-3-(2-(4-(naphthalen-2-yl)thiazol-2-yl)hydrazono)indolin-2-one (4y)**<br>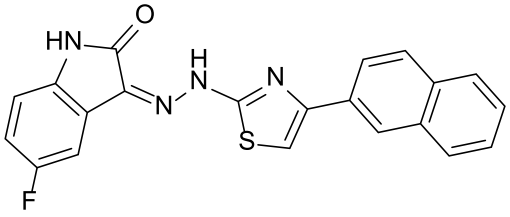	mp: 289-290°C.^{1}H NMR (400MHz, d_6-DMSO): δ 13.46 (s, 1H, -NH), 11.29 (s, 1H, NH of lactam), 8.48 (s, 1H, arom H), 8.06-7.91 (m, 4H, arom H), 7.82 (s, 1H, Thiazole **H**), 7.56-7.50 (m, 2H, arom H), 7.38 (dd, *J*=8.2 Hz, *J*=2.6 Hz,1H, arom H), 7.19 (td, *J*=9.6 Hz, *J*=2.6 Hz, 1H, arom H), 6.98 (dd, *J*=8.6 Hz, *J*=4.2 Hz, 1H, arom H). MS (ESI): $[M+H]^{+}$389.33.

Copies of ^{1}H NMR and Mass spectra of thiazolylhydrazonoindolin-2-ones (4)

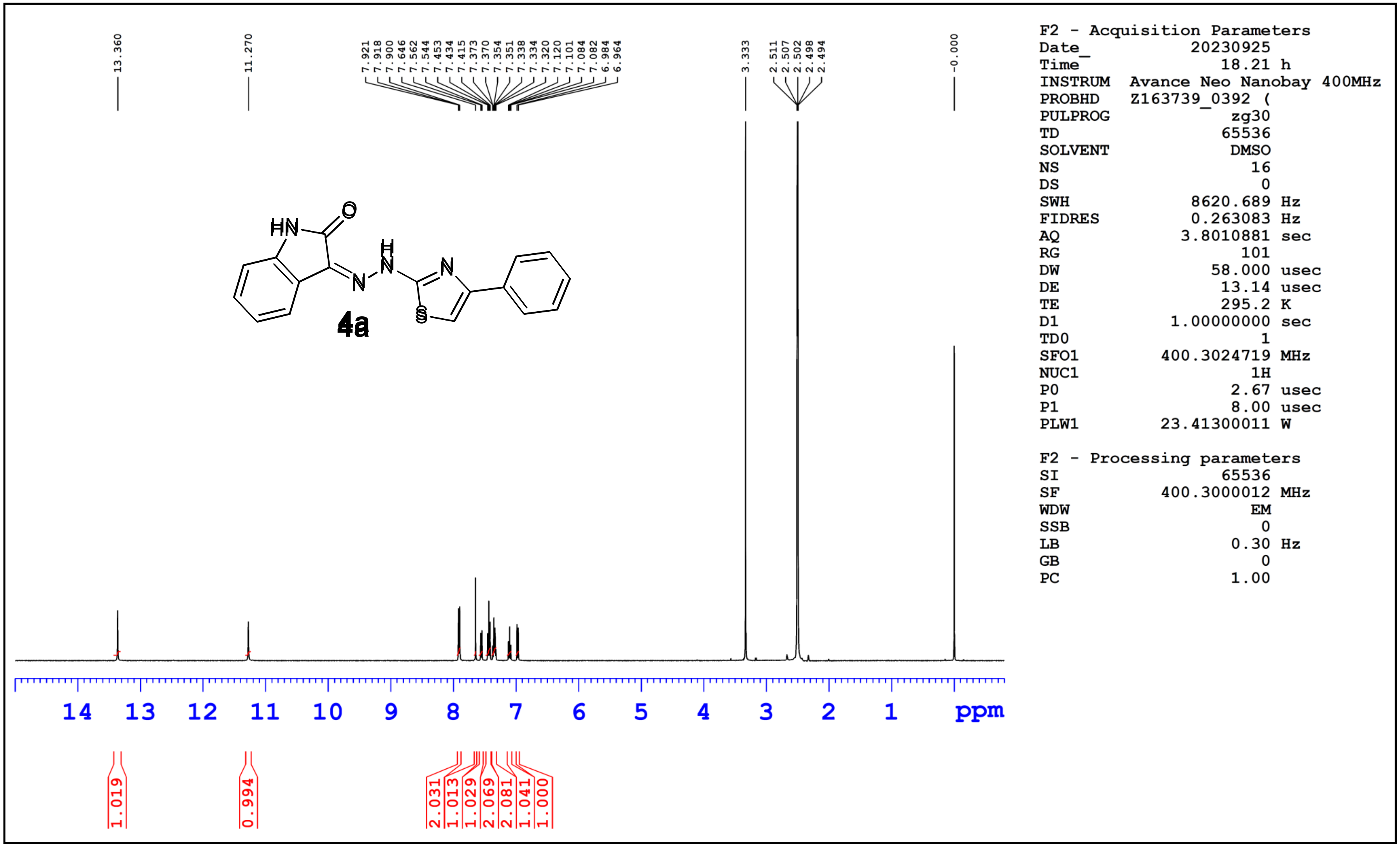

Figure S1 ^{1}H NMR spectrum of 4a.

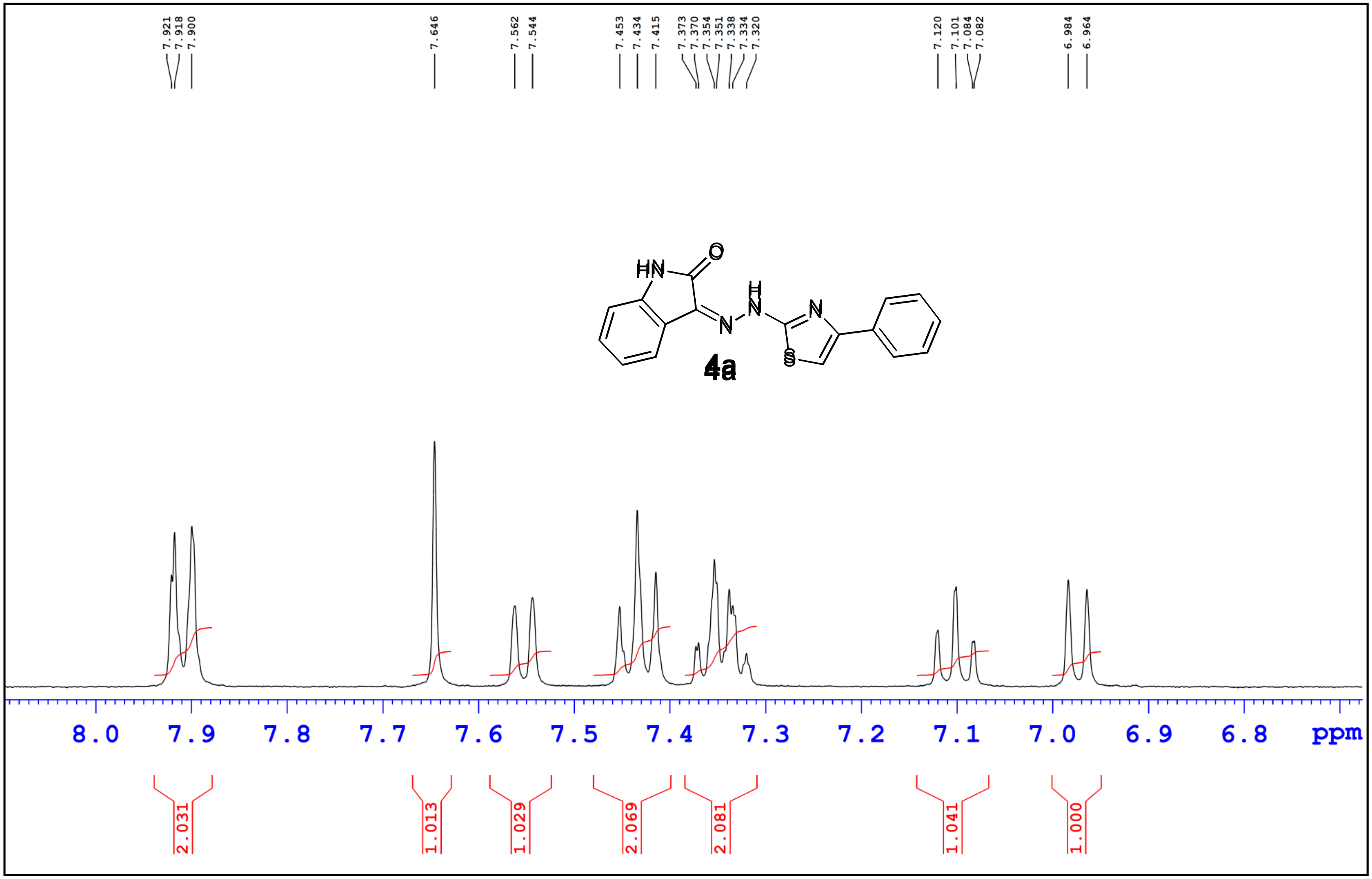

Figure S2 Expanded ^{1}H NMR spectrum of 4a.

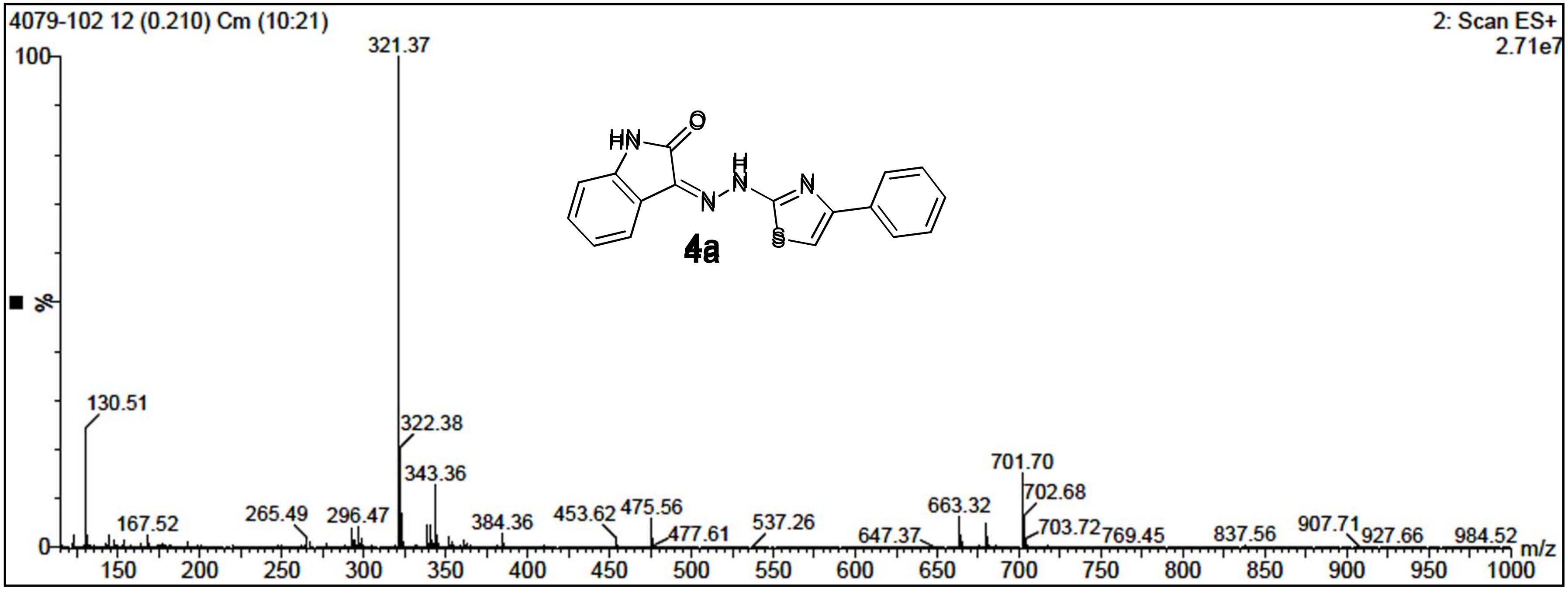

Figure S3Mass spectrum of 4a.

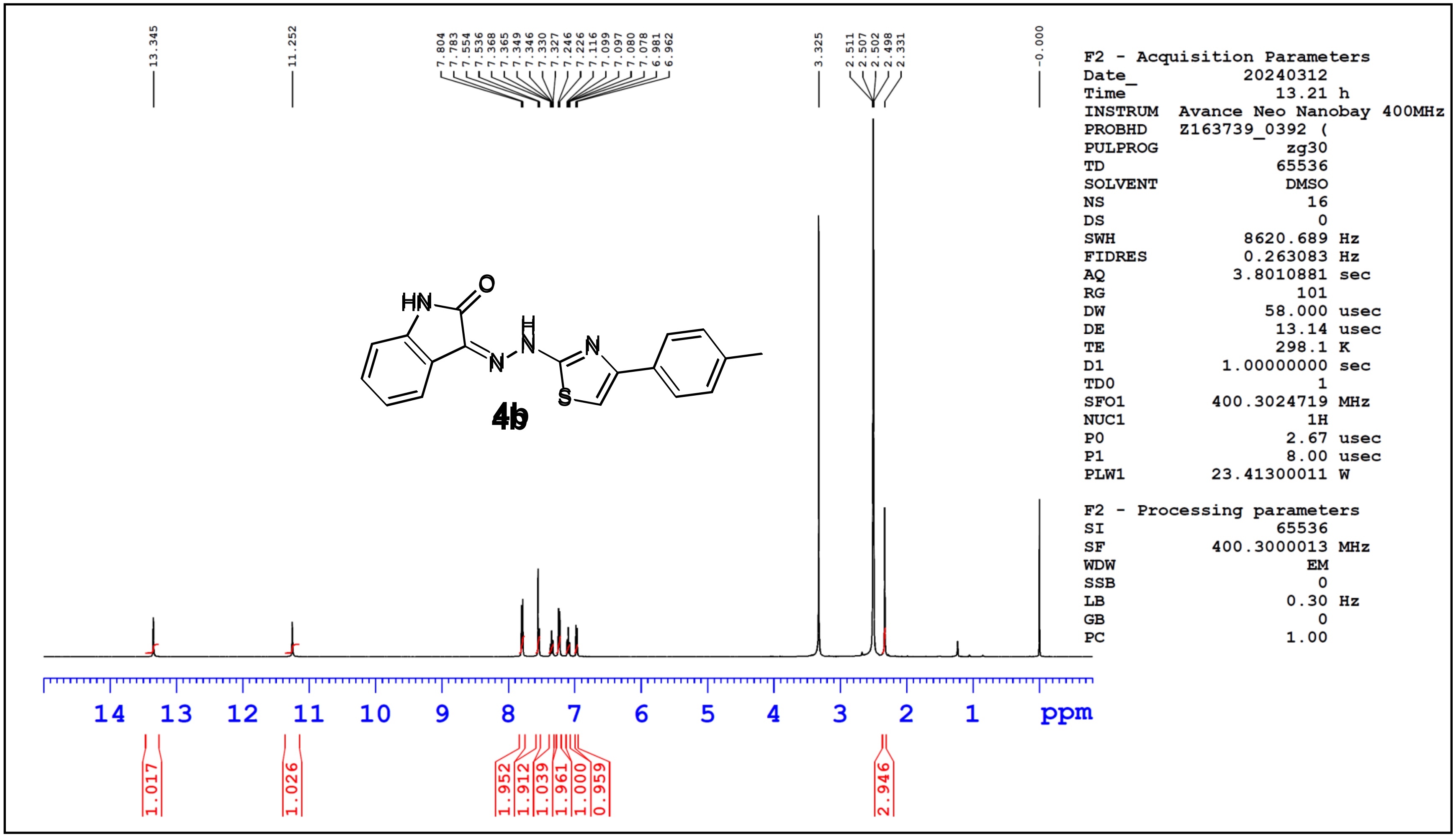

Figure S4^{1}H NMR spectrum of 4b.

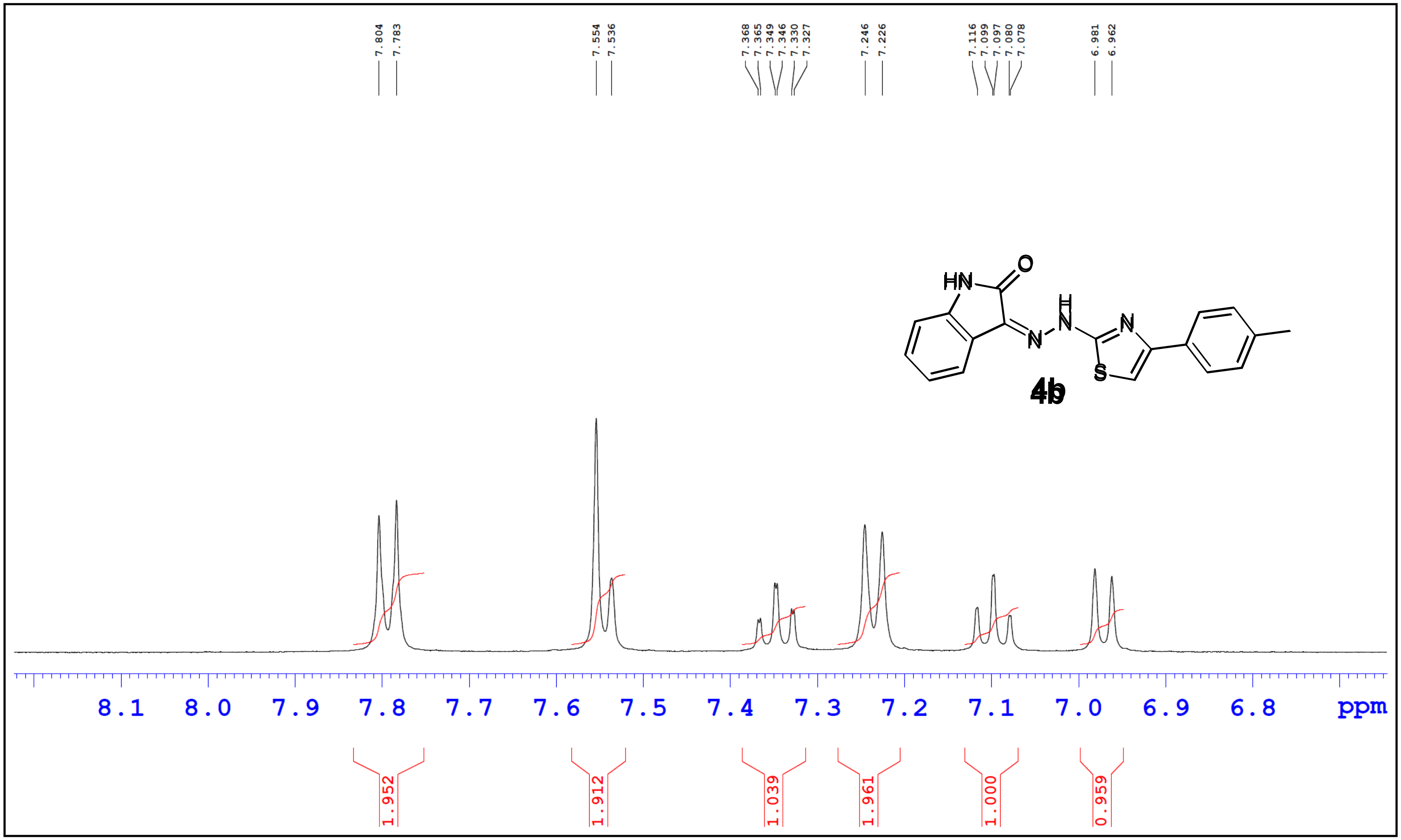

Figure S5 Expanded ^{1}H NMR spectrum of 4b.

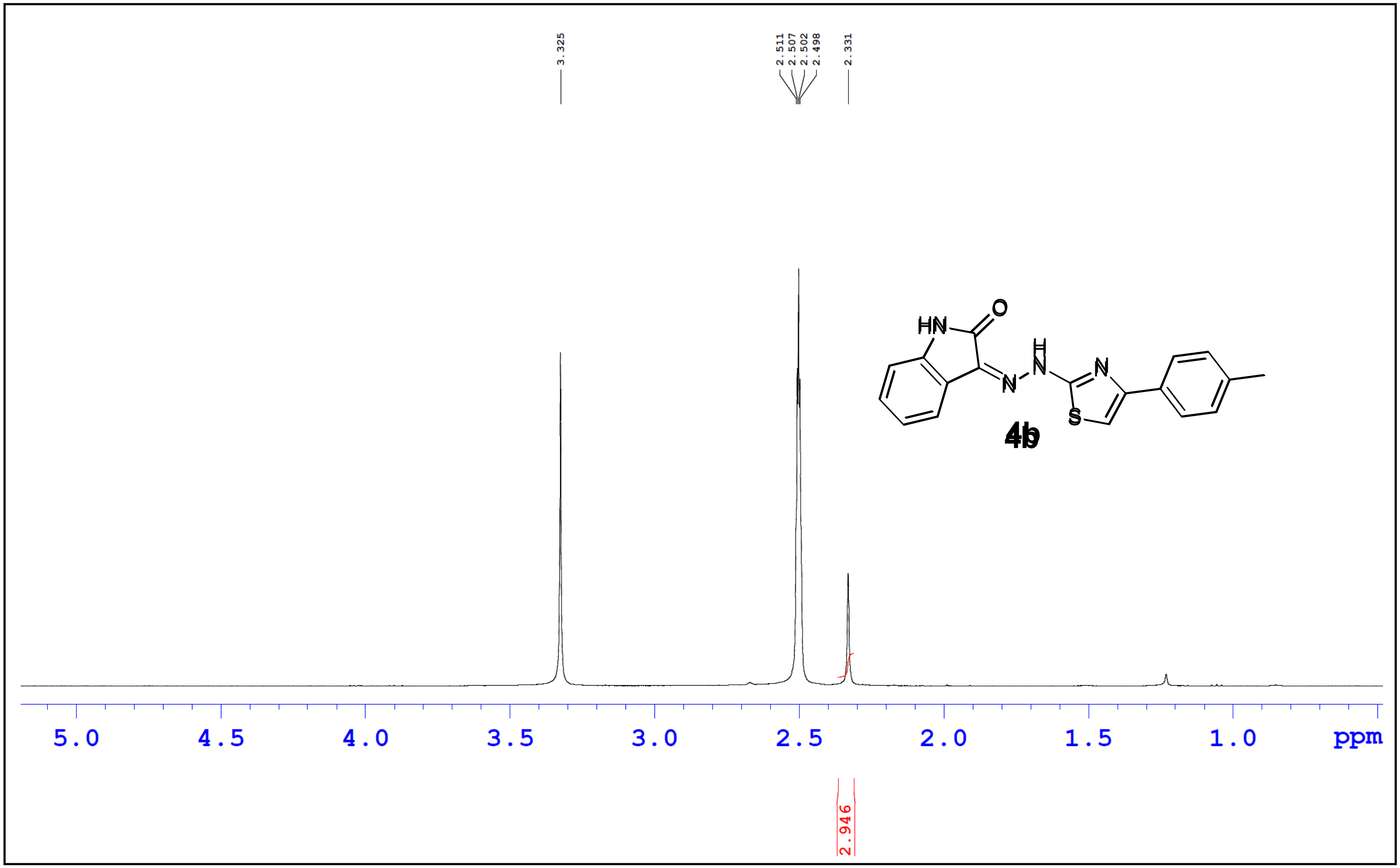

Figure S6 Expanded ^{1}H NMR spectrum of 4b.

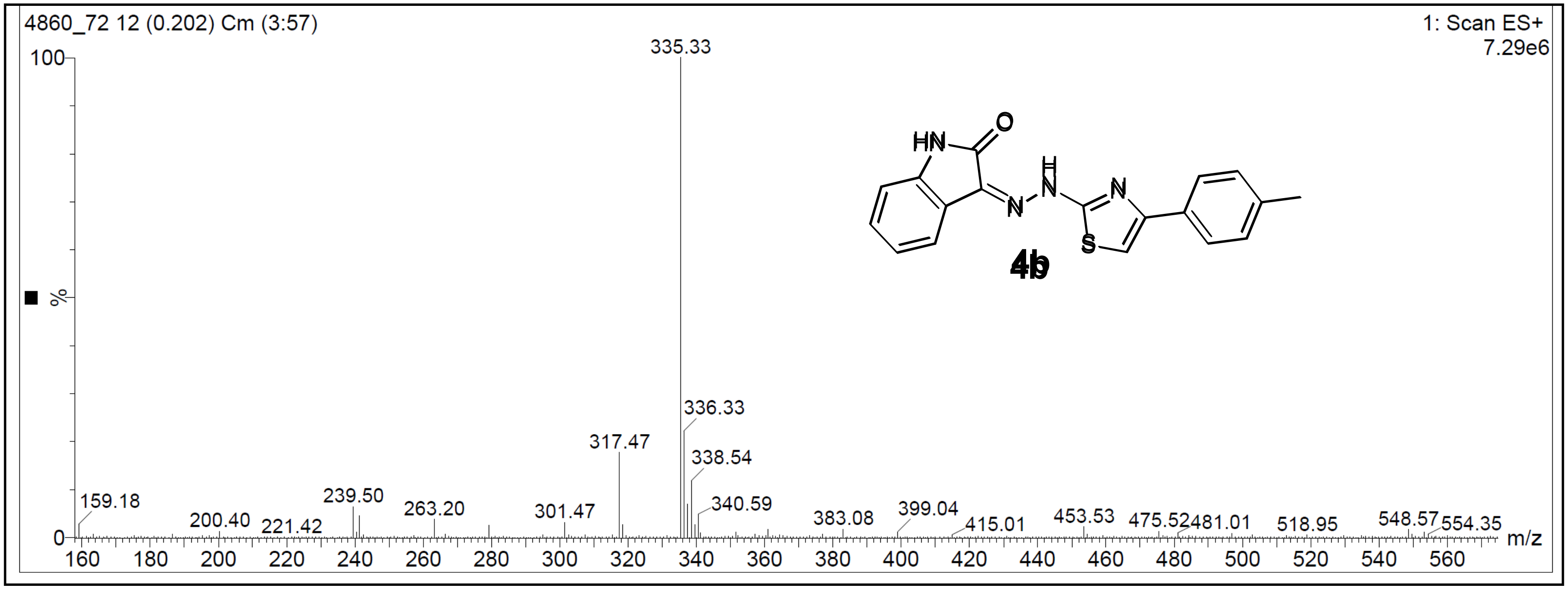

Figure S7Mass spectrum of 4b.

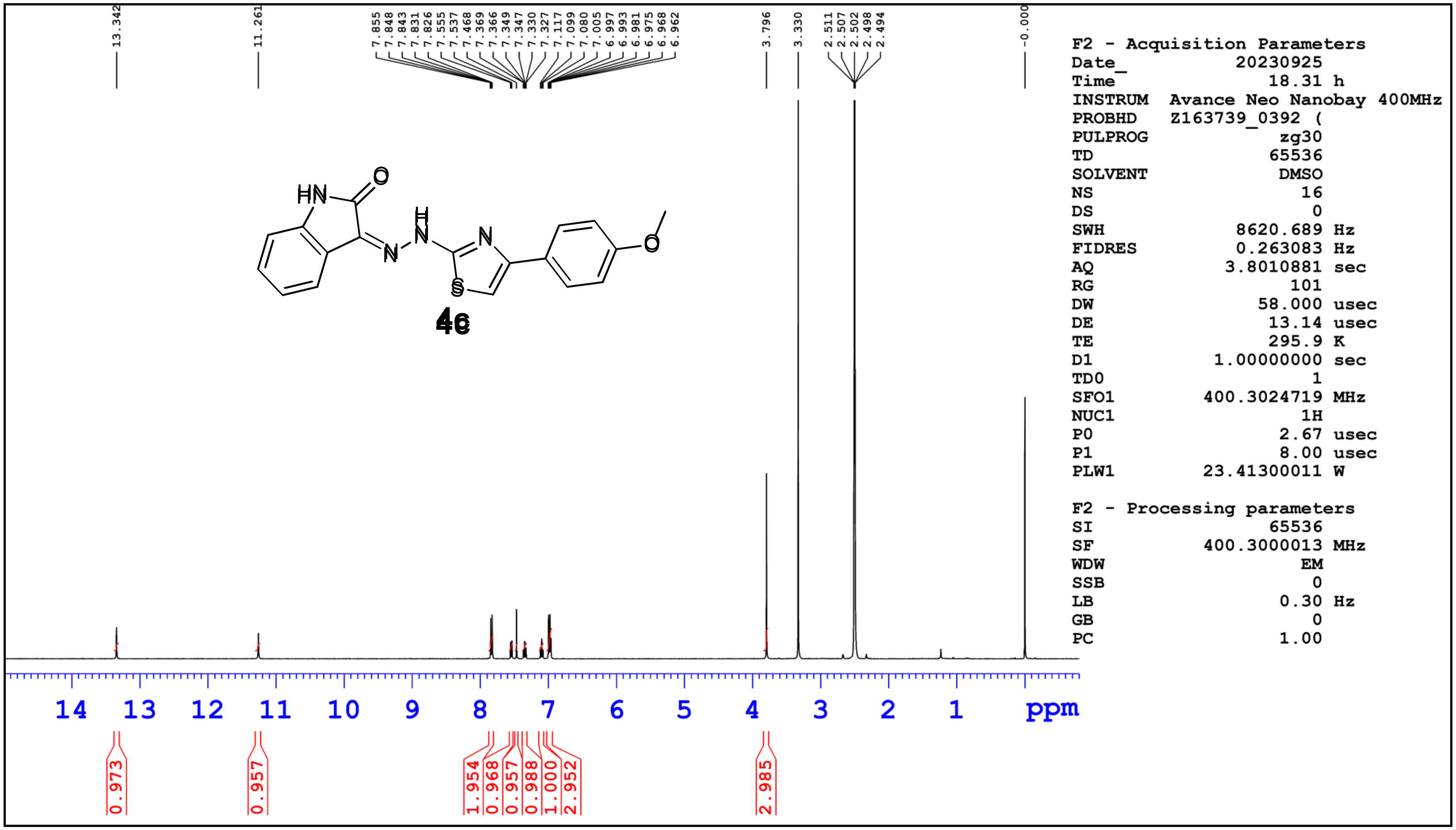

Figure S8^{1}H NMR spectrum of 4c.

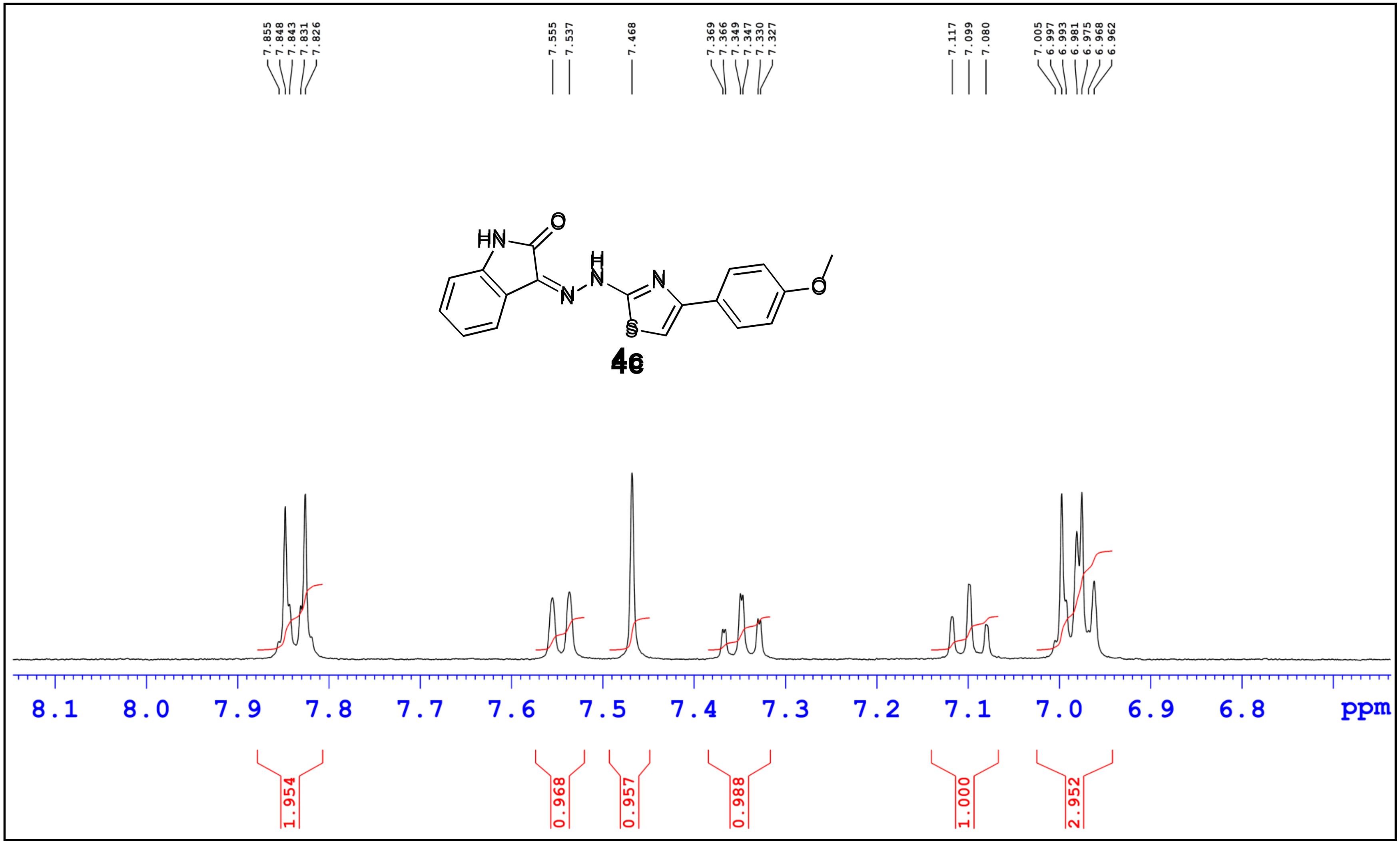

Figure S9 Expanded ^{1}H NMR spectrum of 4c.

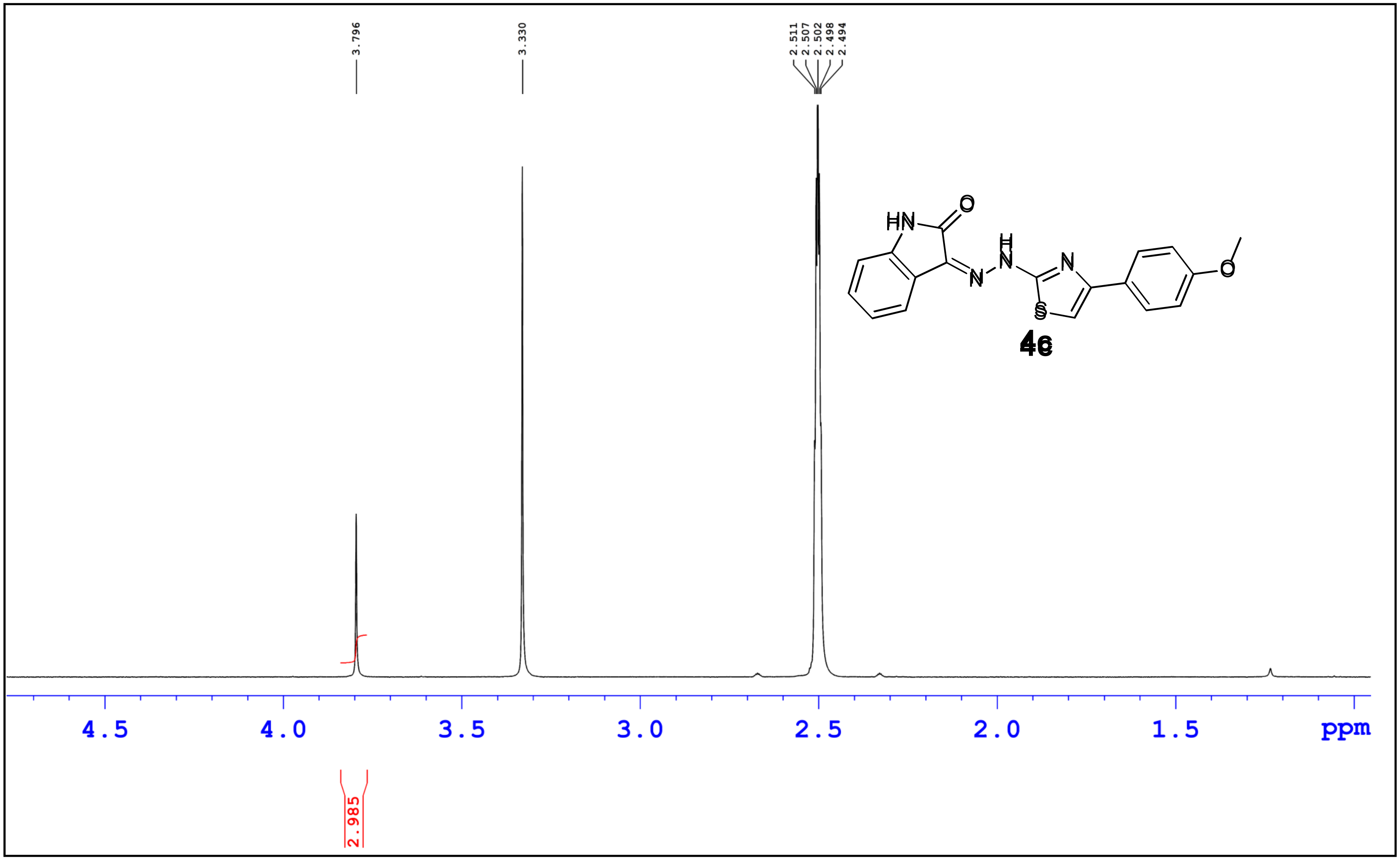

Figure S10 Expanded ^{1}H NMR spectrum of 4c.

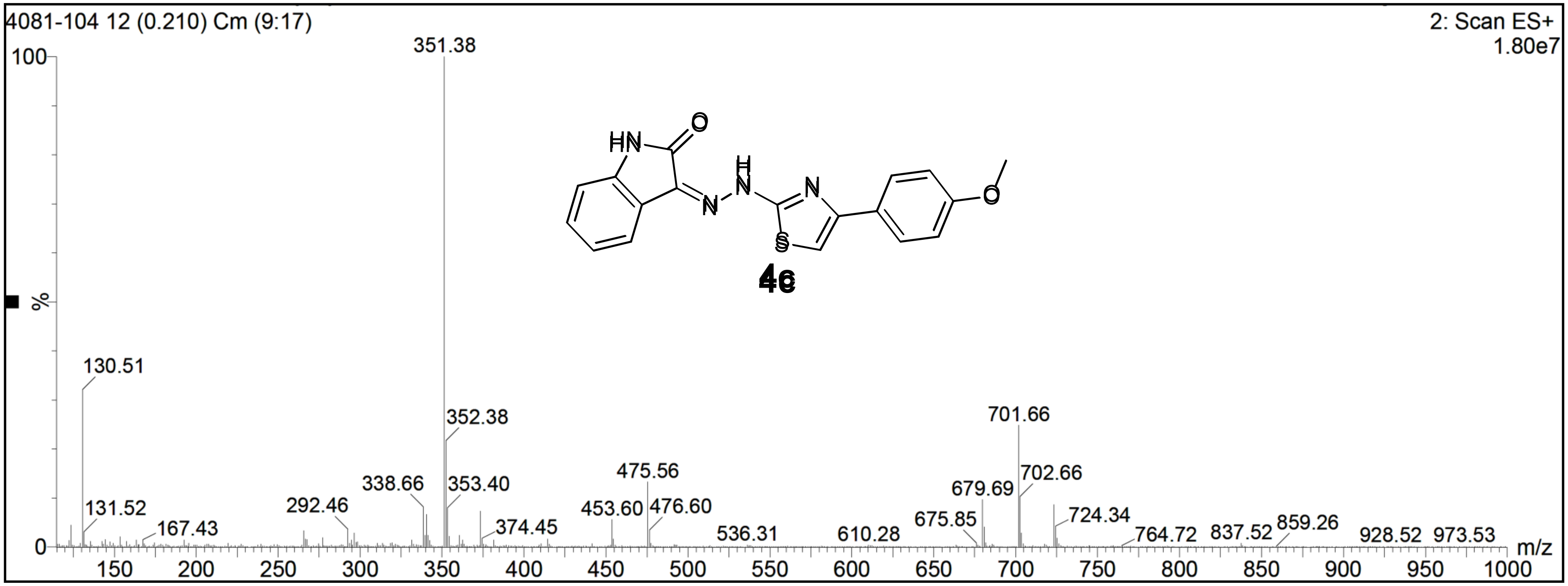

Figure S11 Mass spectrum of 4c.

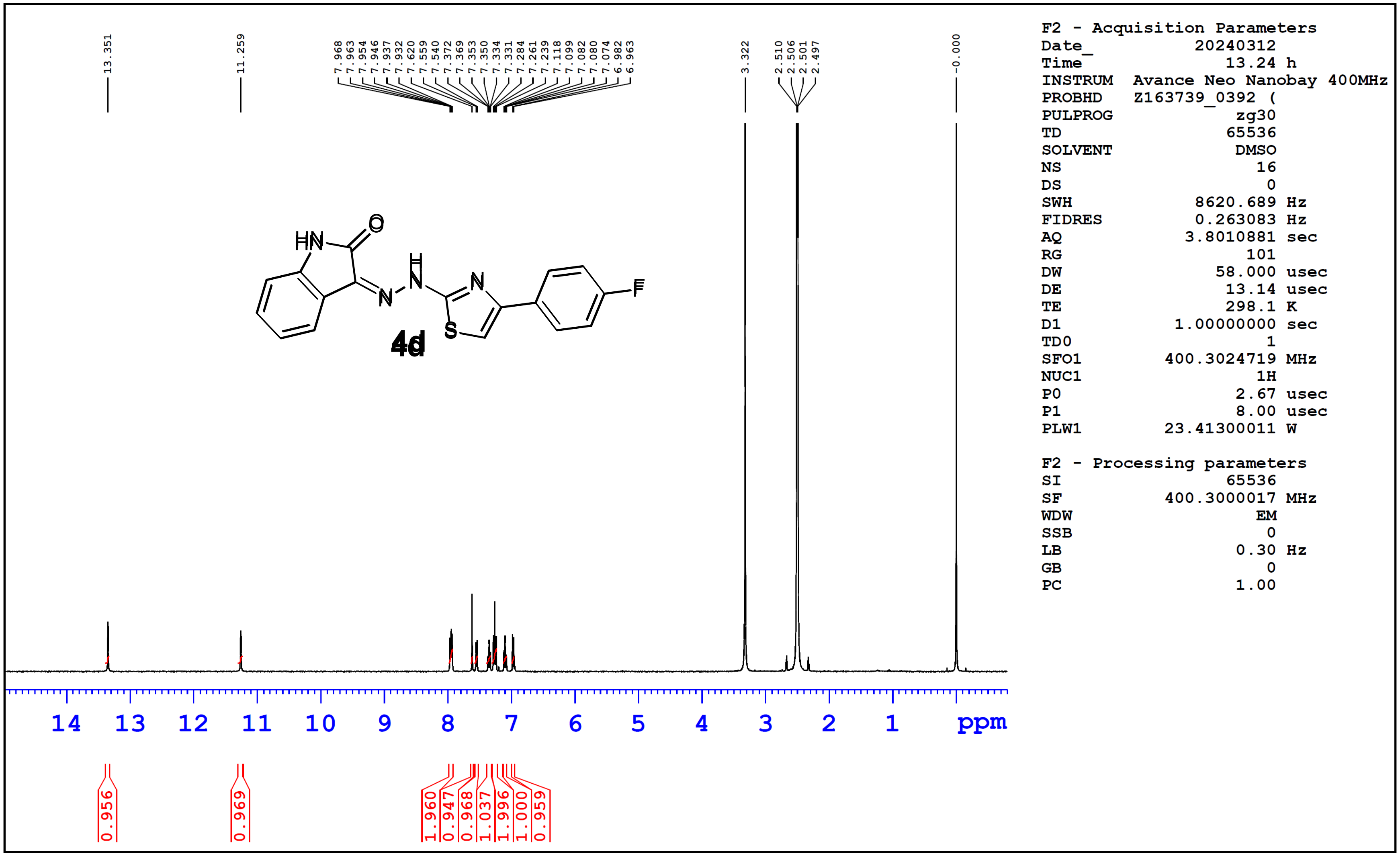

Figure S12^{1}H NMR spectrum of 4d.

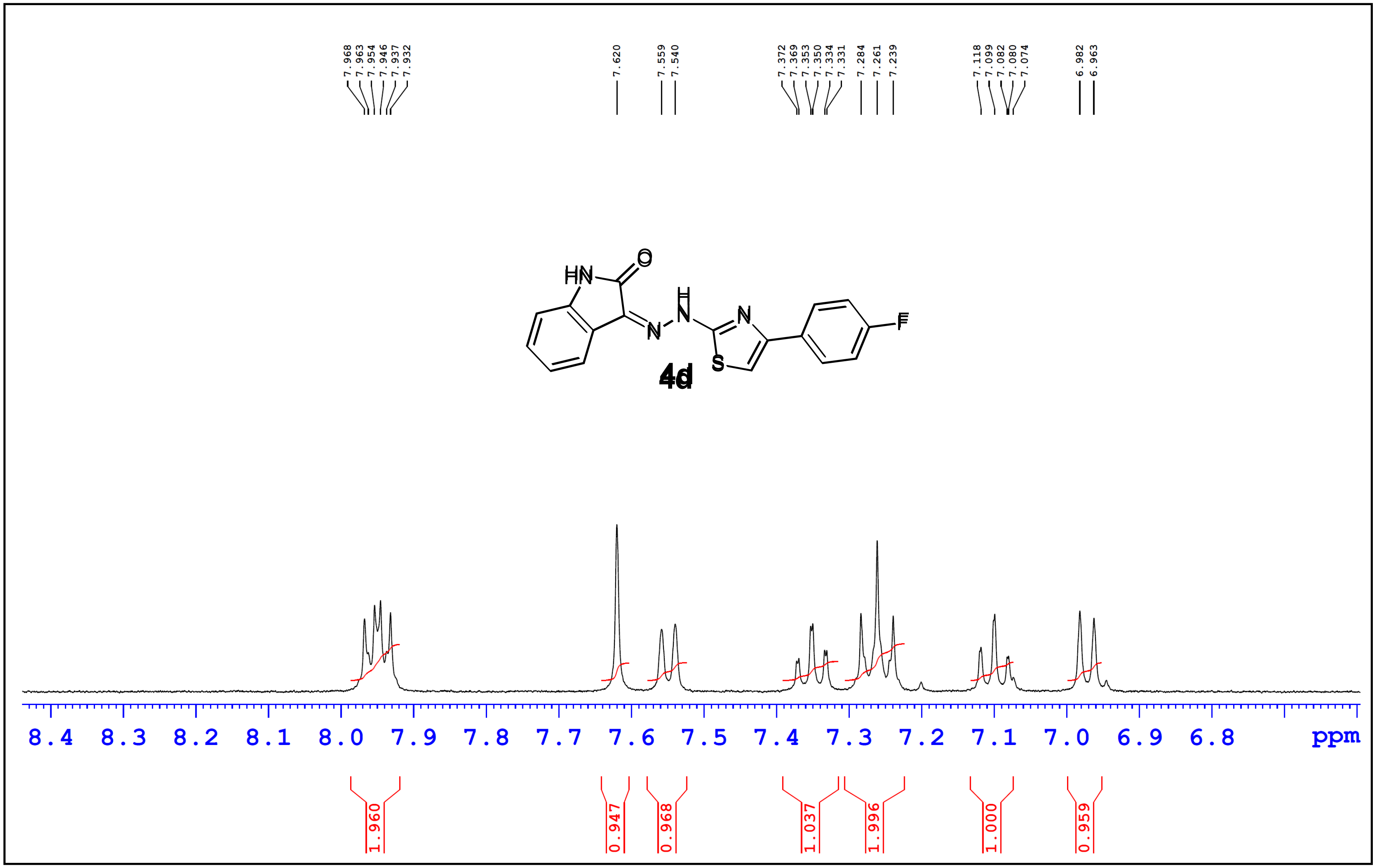

Figure S13 Expanded ^{1}H NMR spectrum of 4d.

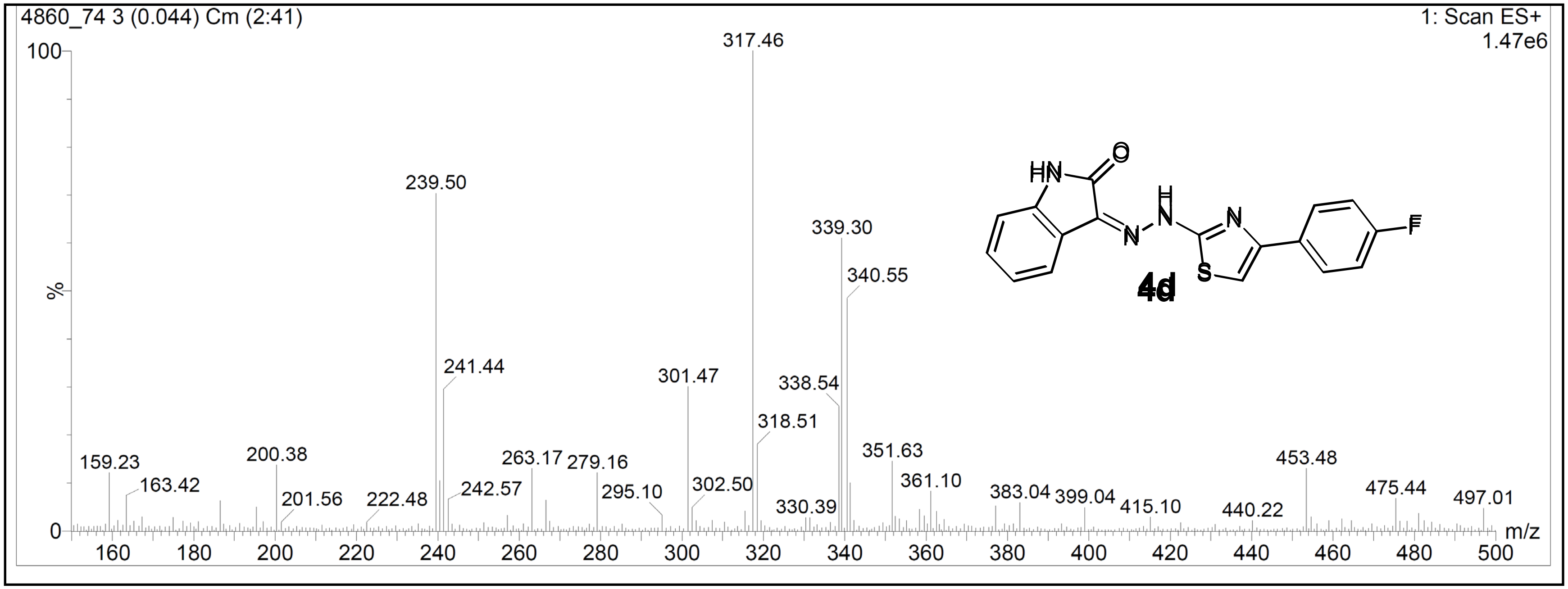

Figure S14 Mass spectrum of 4d.

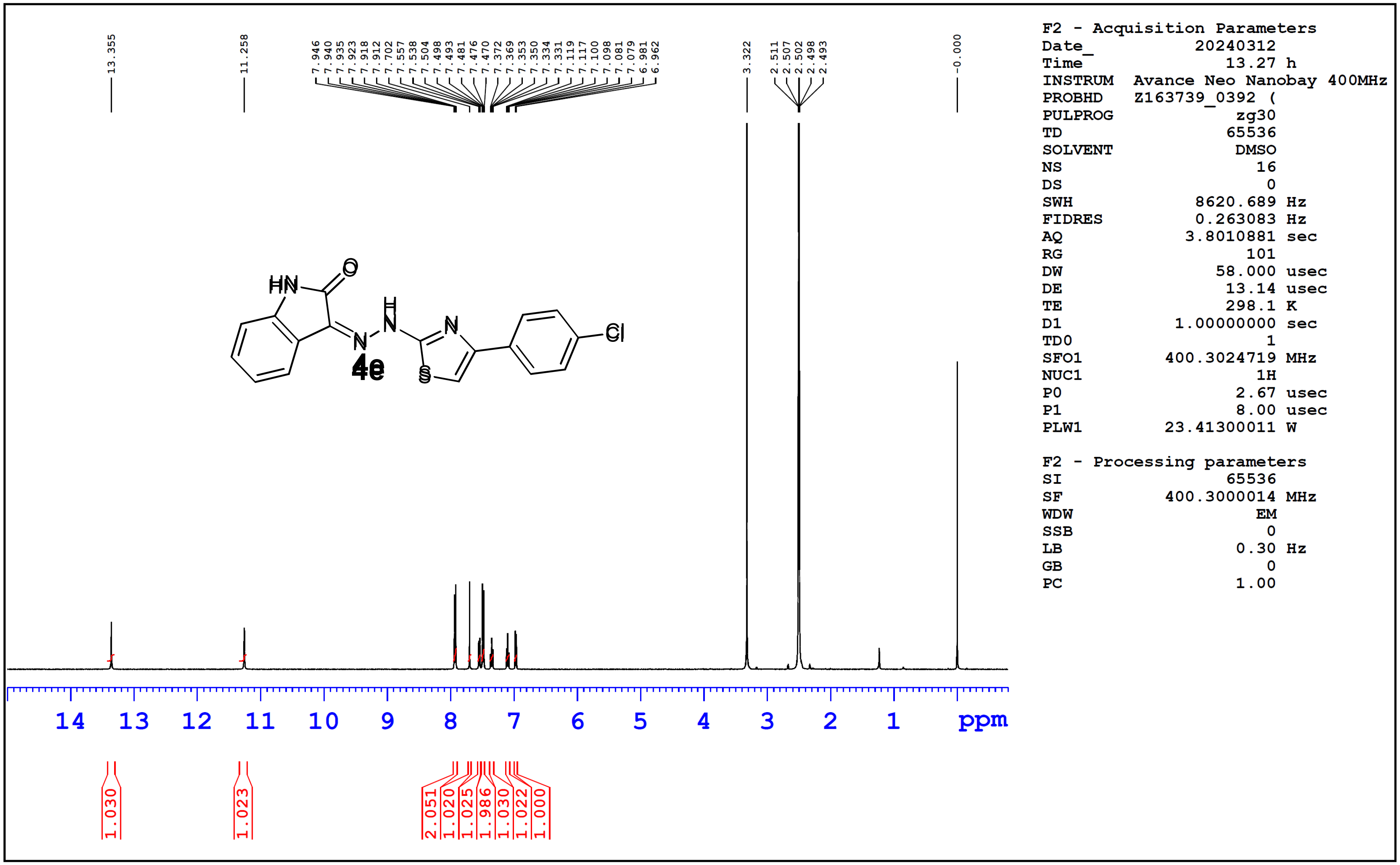

Figure S15^{1}H NMR spectrum of 4e.

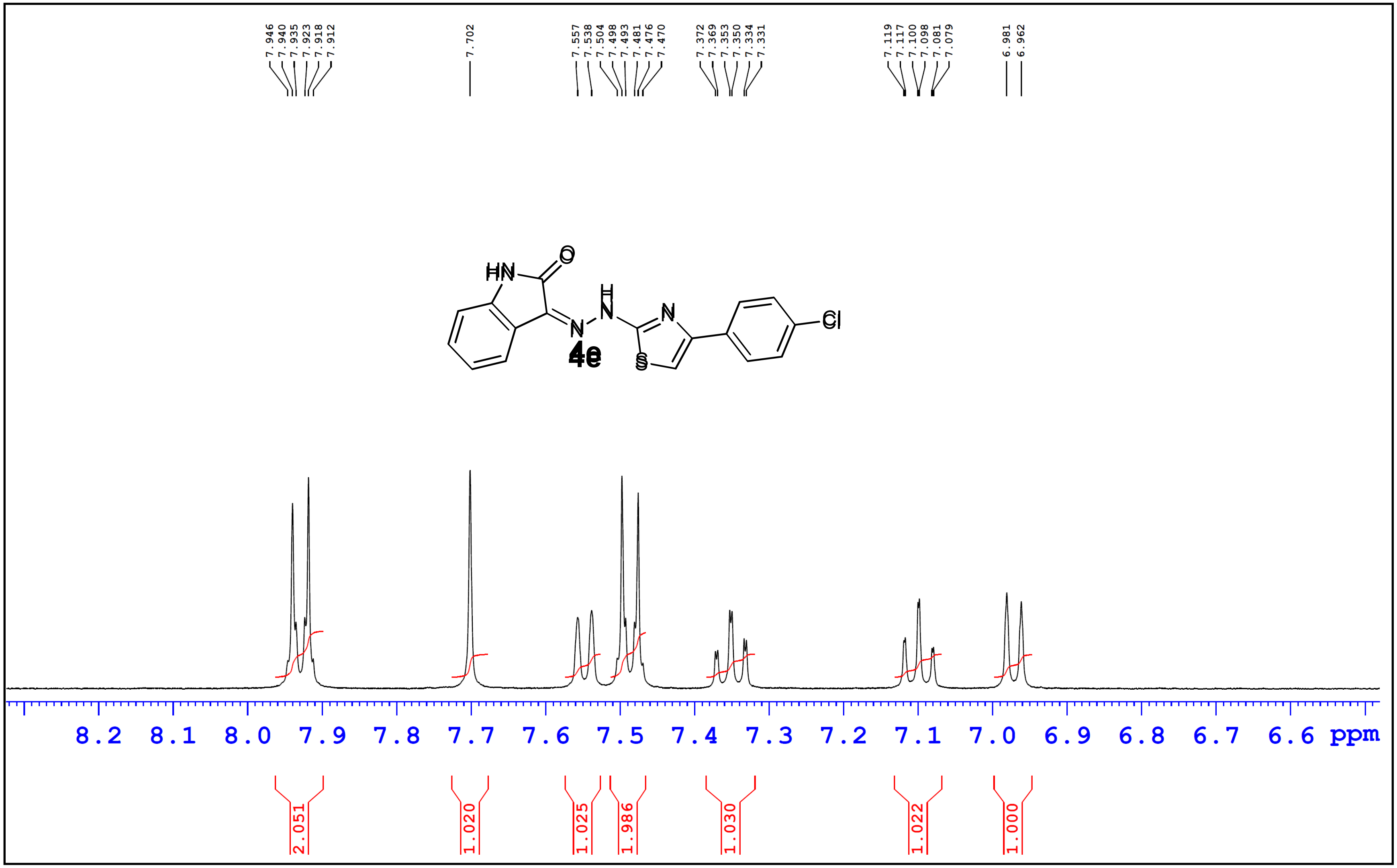

Figure S16 Expanded ^{1}H NMR spectrum of 4e.

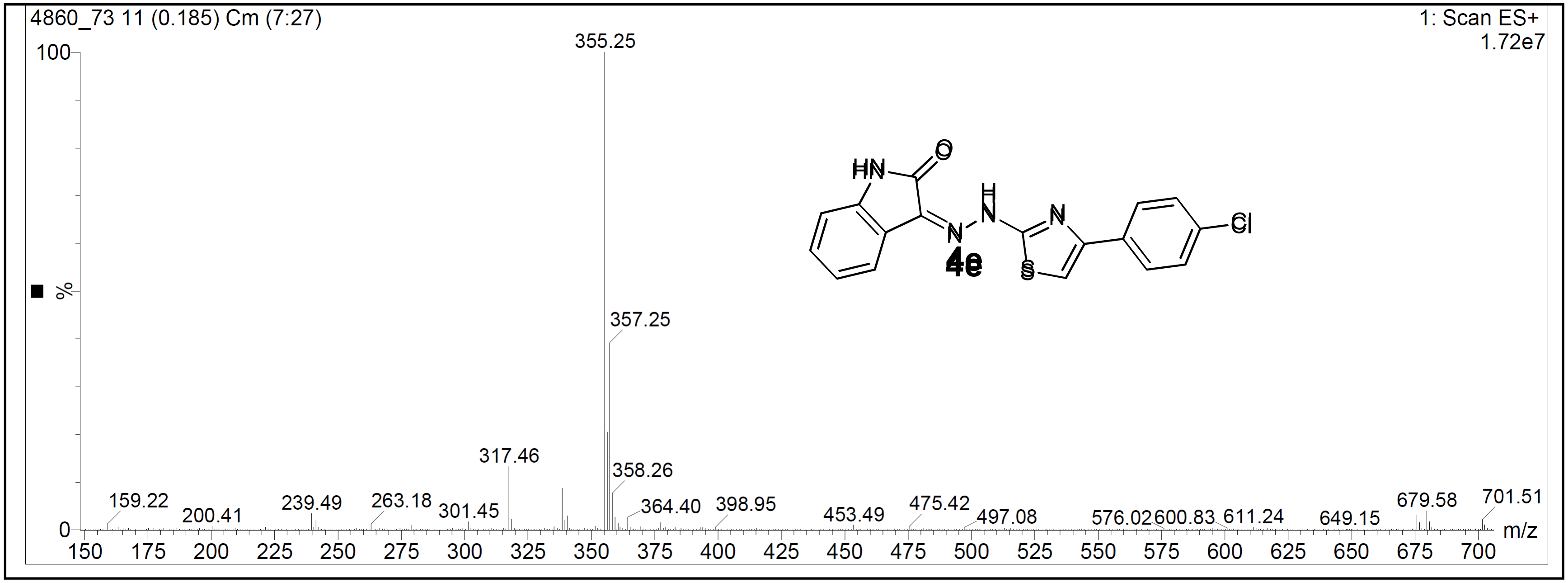

Figure S17 Mass spectrum of 4e.

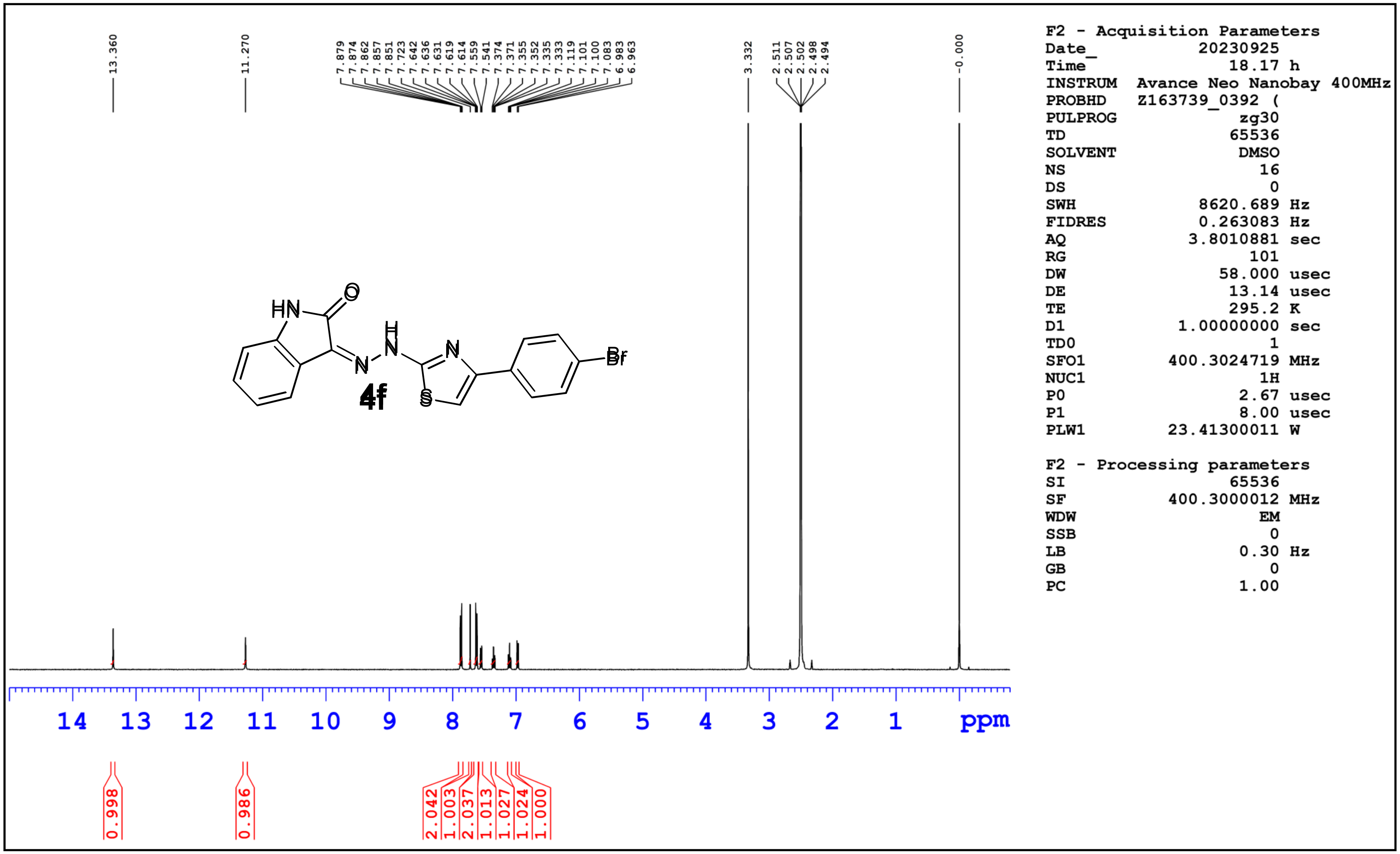

Figure S18^{1}H NMR spectrum of 4f.

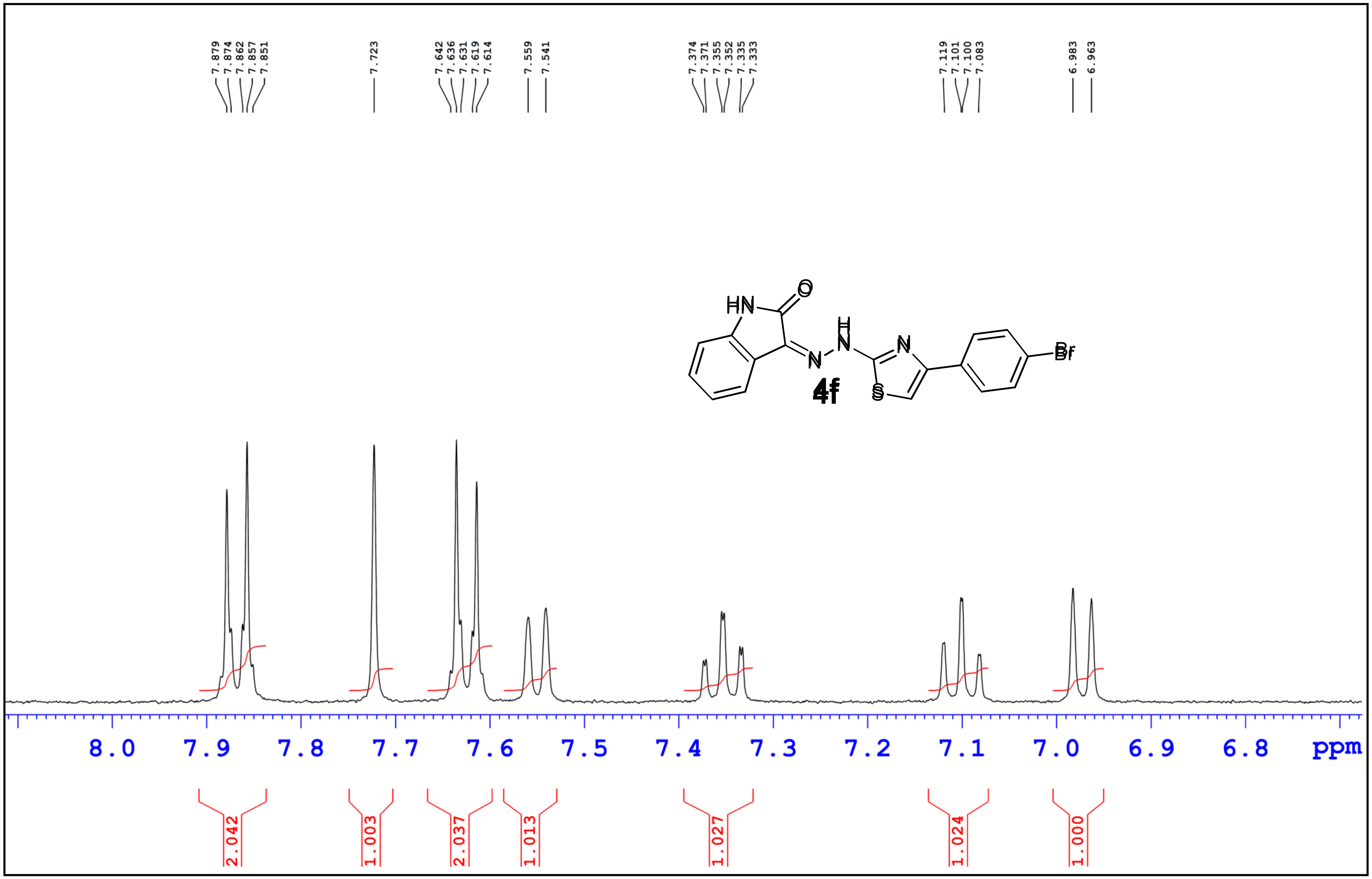

Figure S19 Expanded ^{1}H NMR spectrum of 4f.

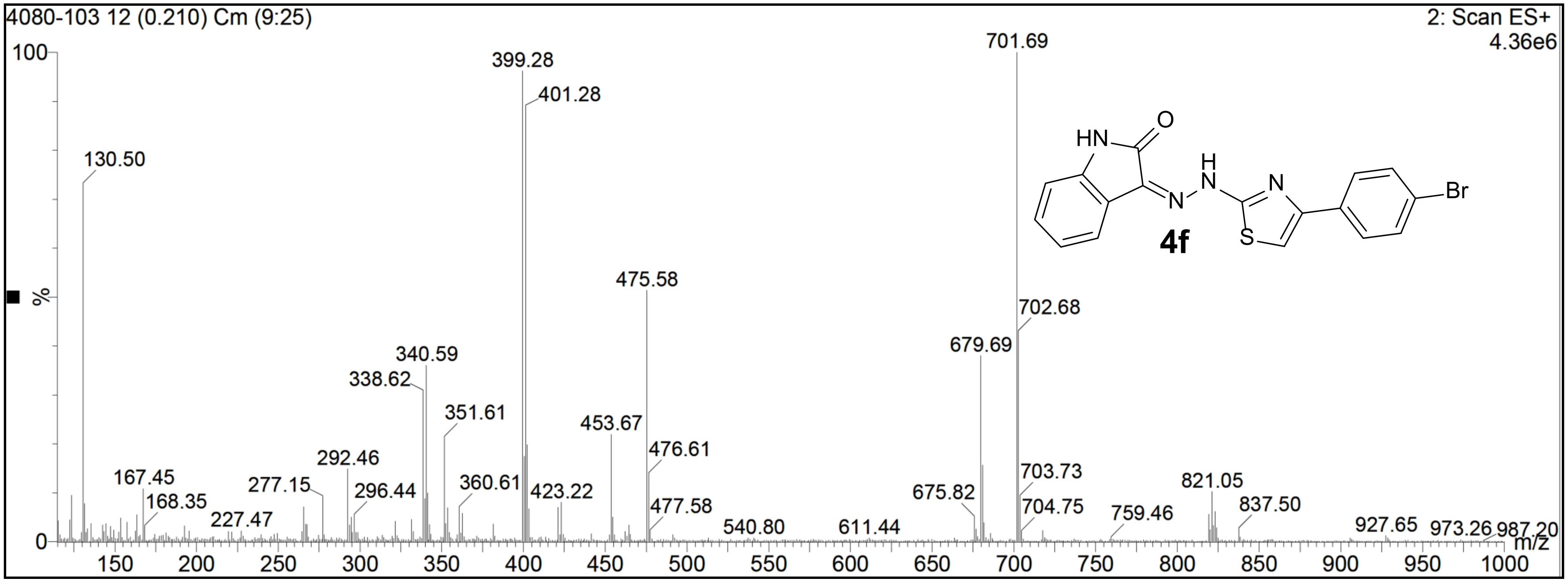

Figure S20Mass spectrum of 4f.

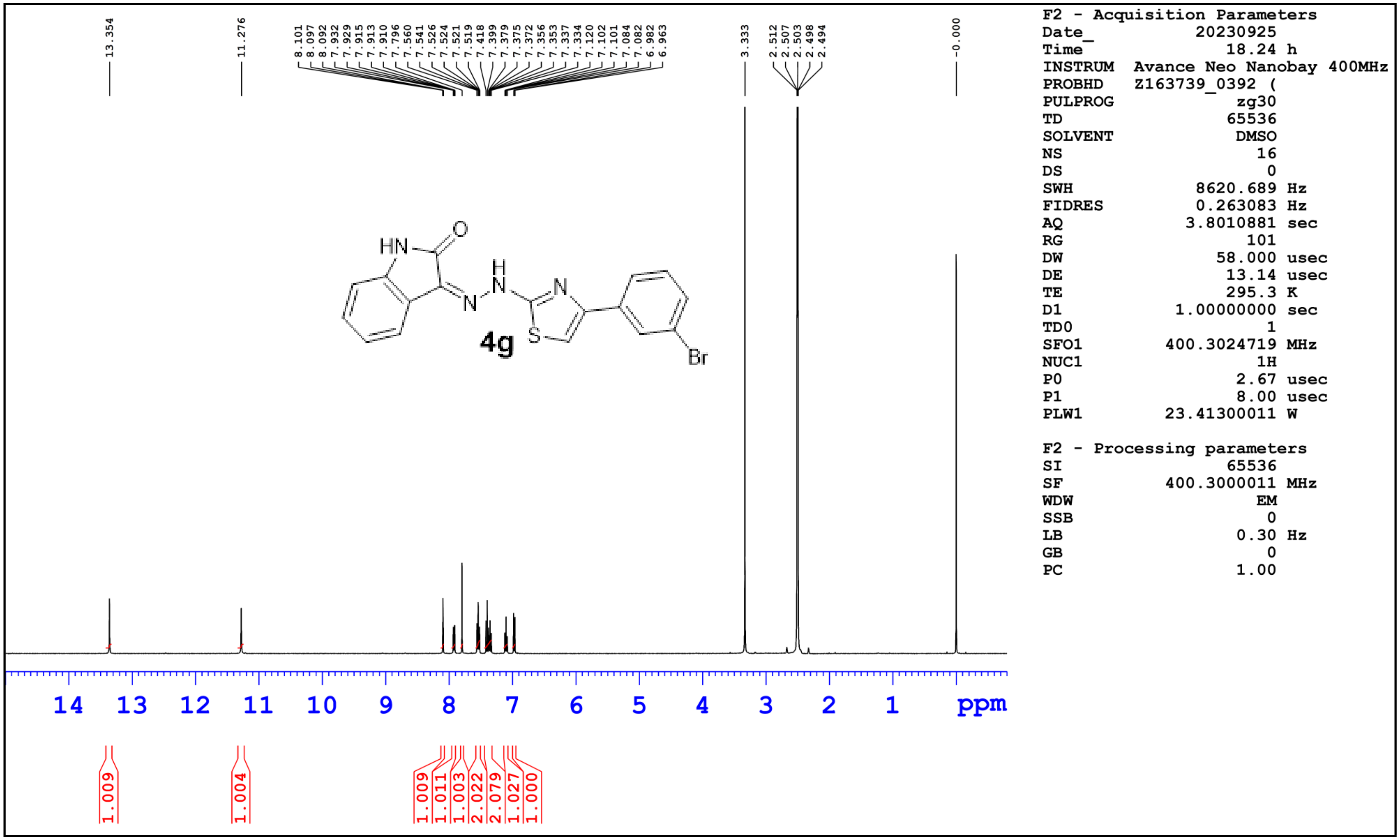

Figure S21^{1}H NMR spectrum of 4g.

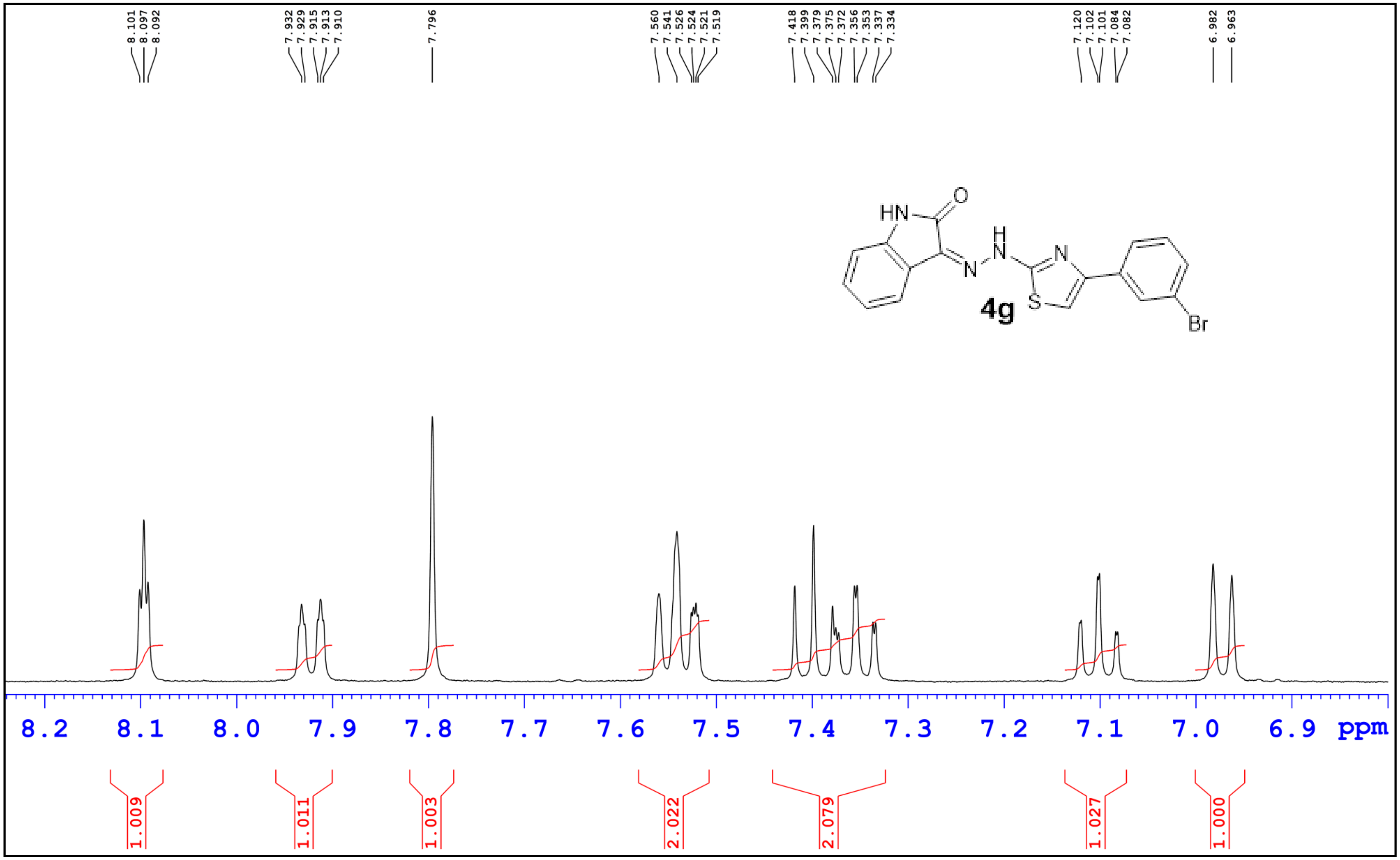

Figure S22 Expanded ^{1}H NMR spectrum of 4g.

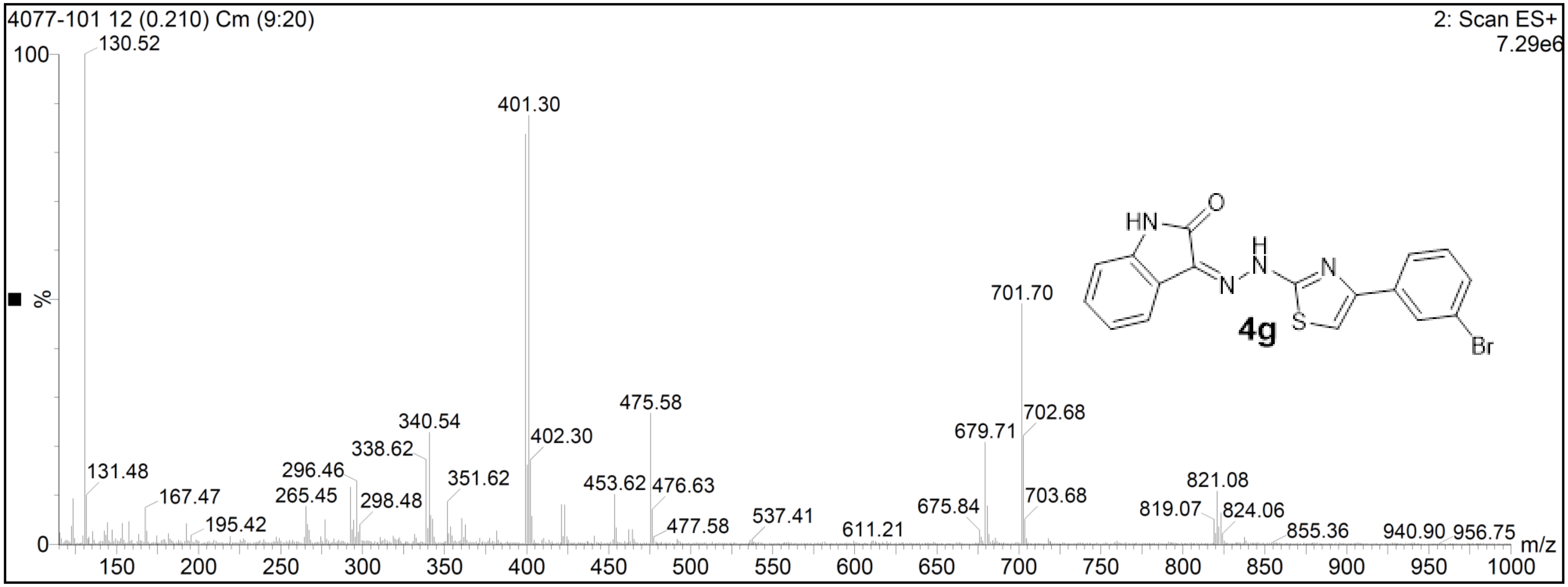

Figure S23Mass spectrum of 4g.

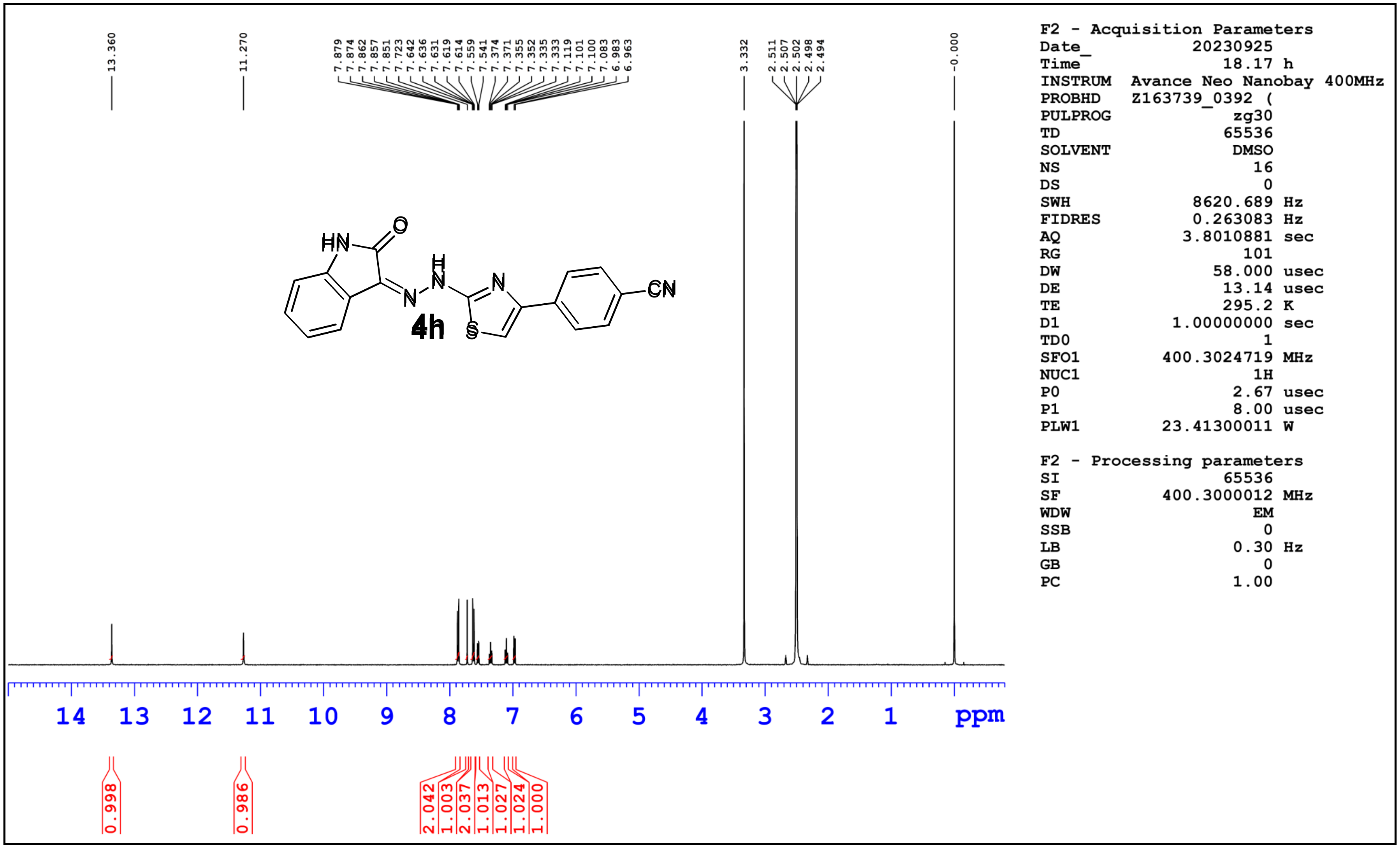

Figure S24 ^{1}H NMR spectrum of 4h.

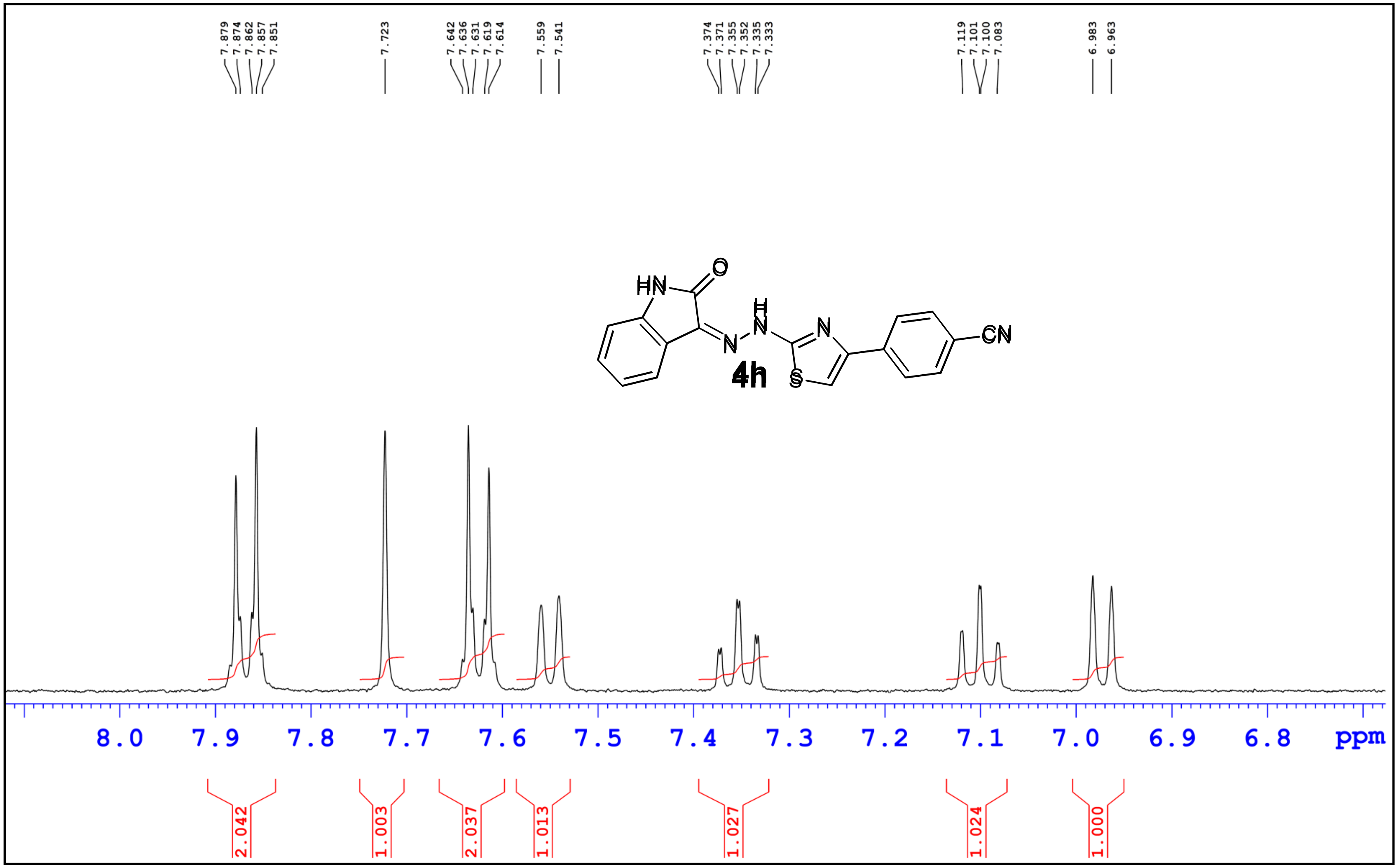

Figure S25 Expanded ^{1}H NMR spectrum of 4h.

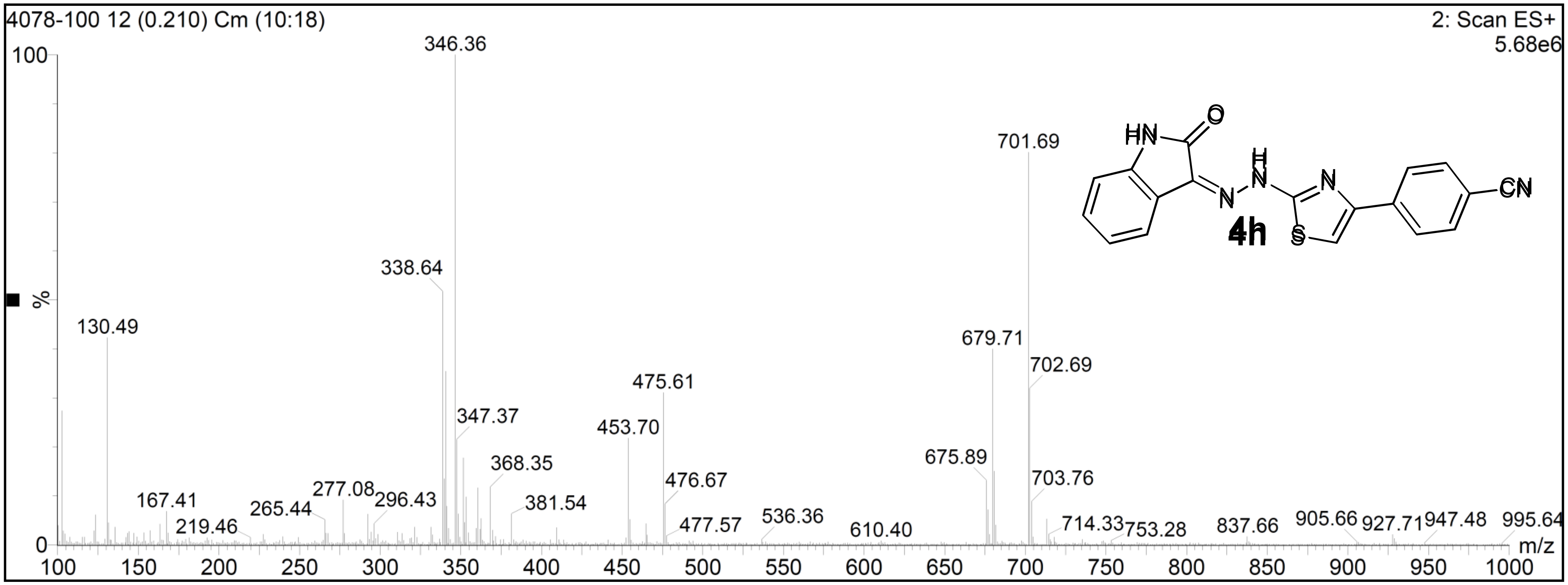

Figure S26 Mass spectrum of 4h.

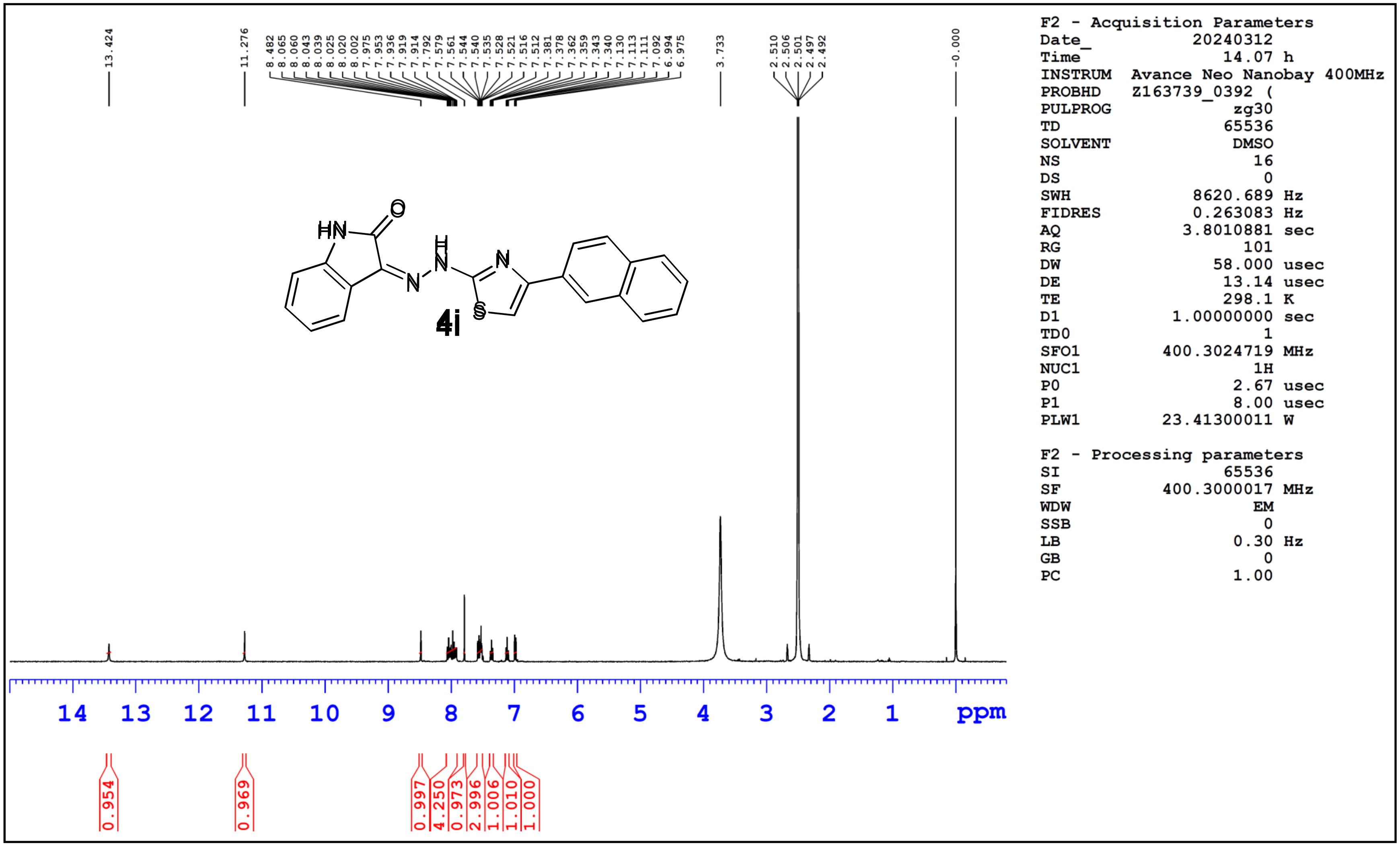

Figure S 27^{1}H NMR spectrum of 4i.

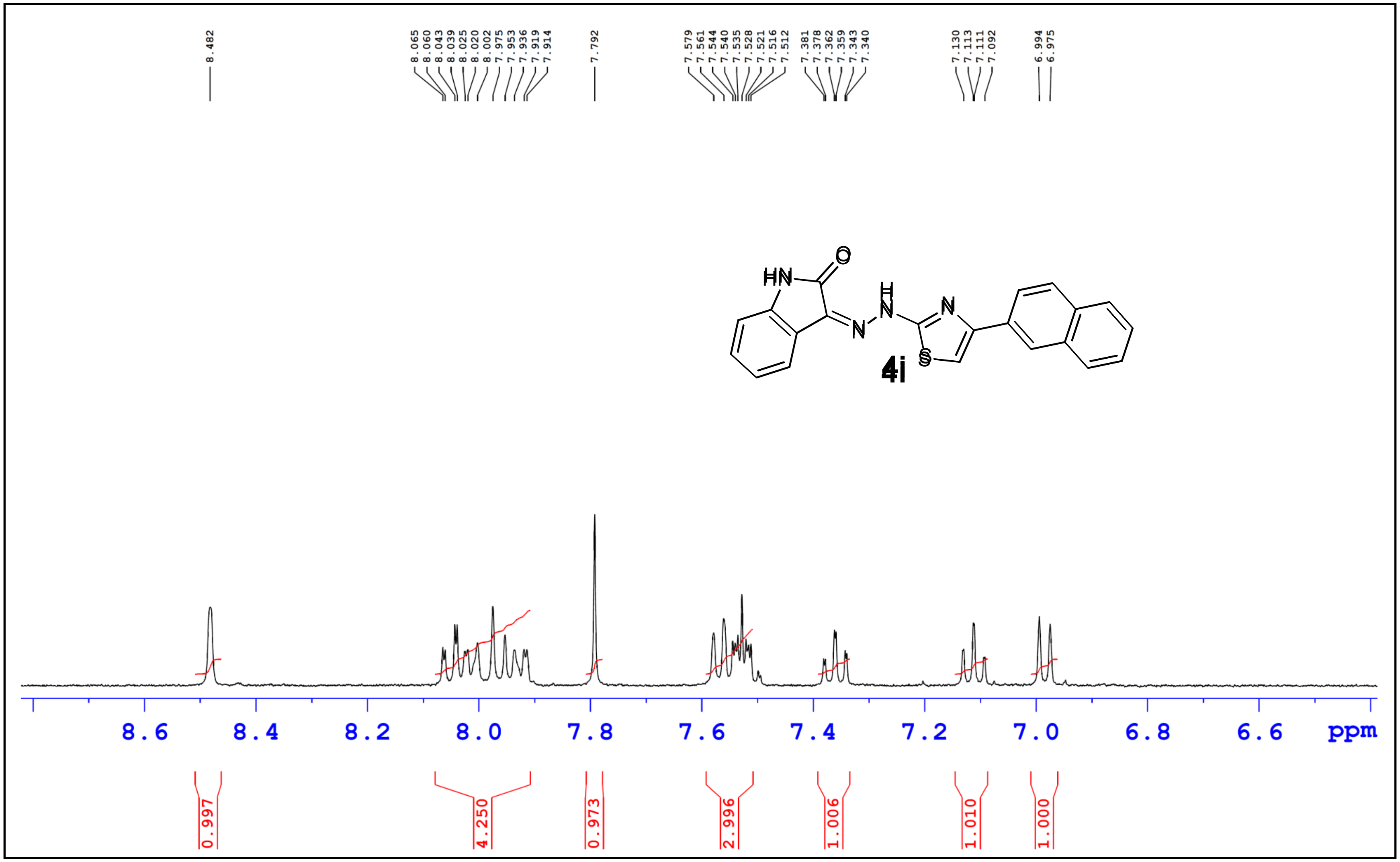

Figure S28 Expanded ^{1}H NMR spectrum of 4i.

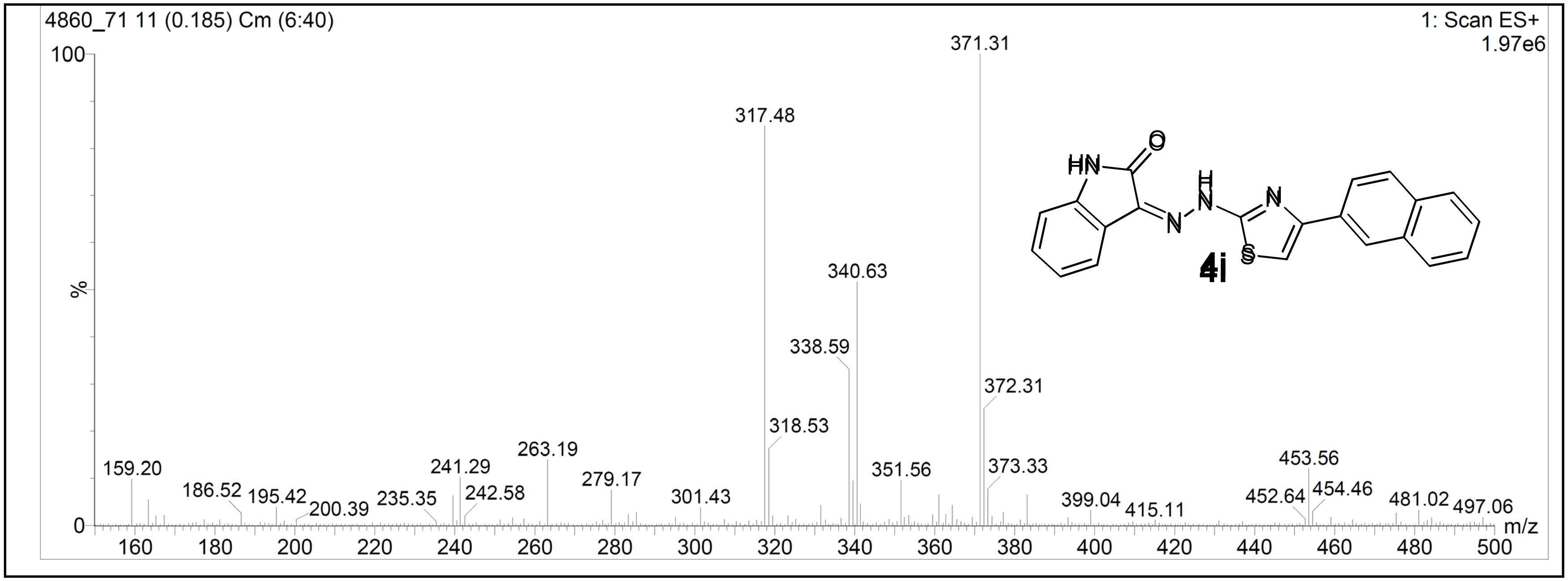

Figure S29 Mass spectrum of 4i.

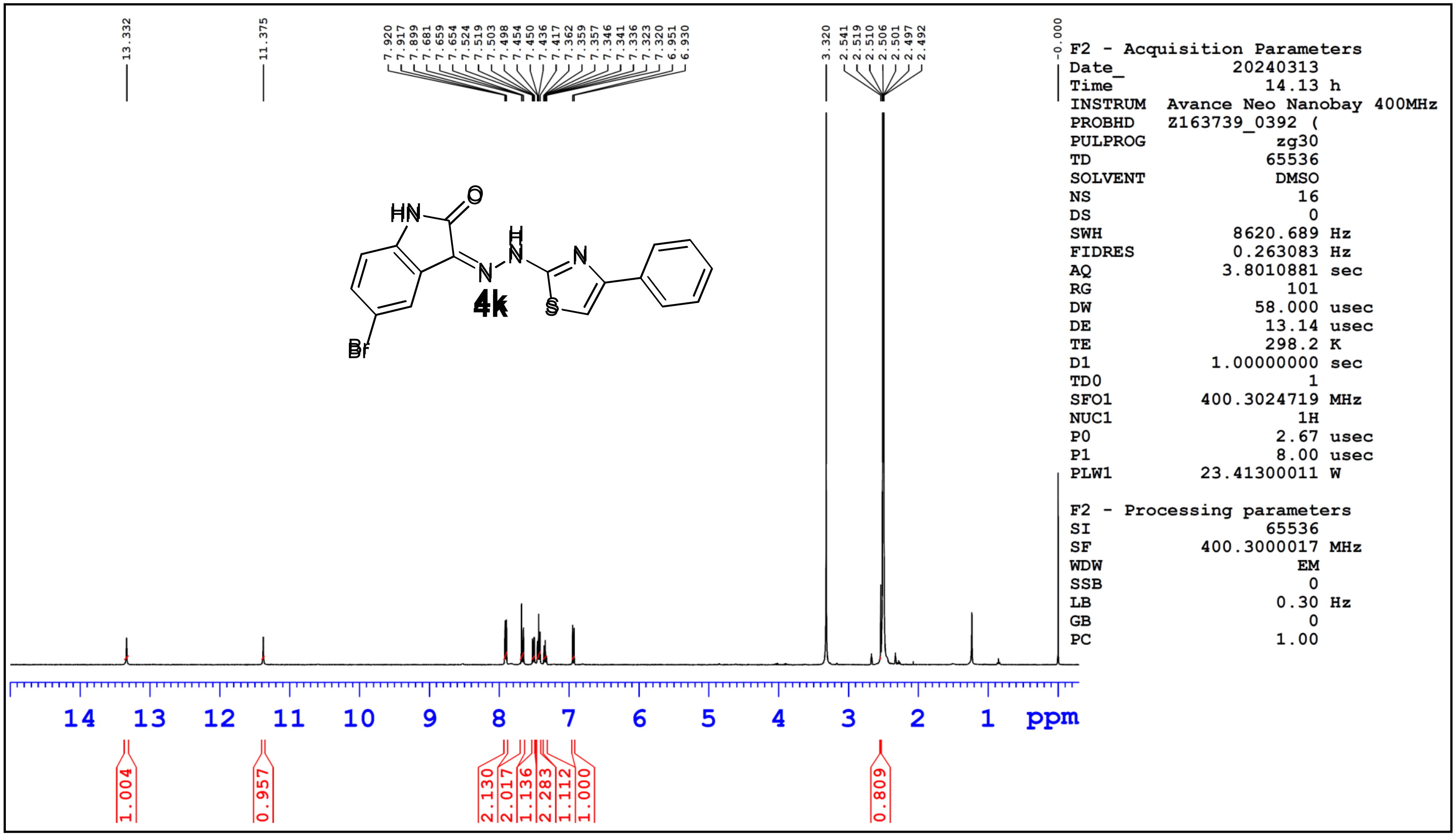

Figure S30^{1}H NMR spectrum of 4k.

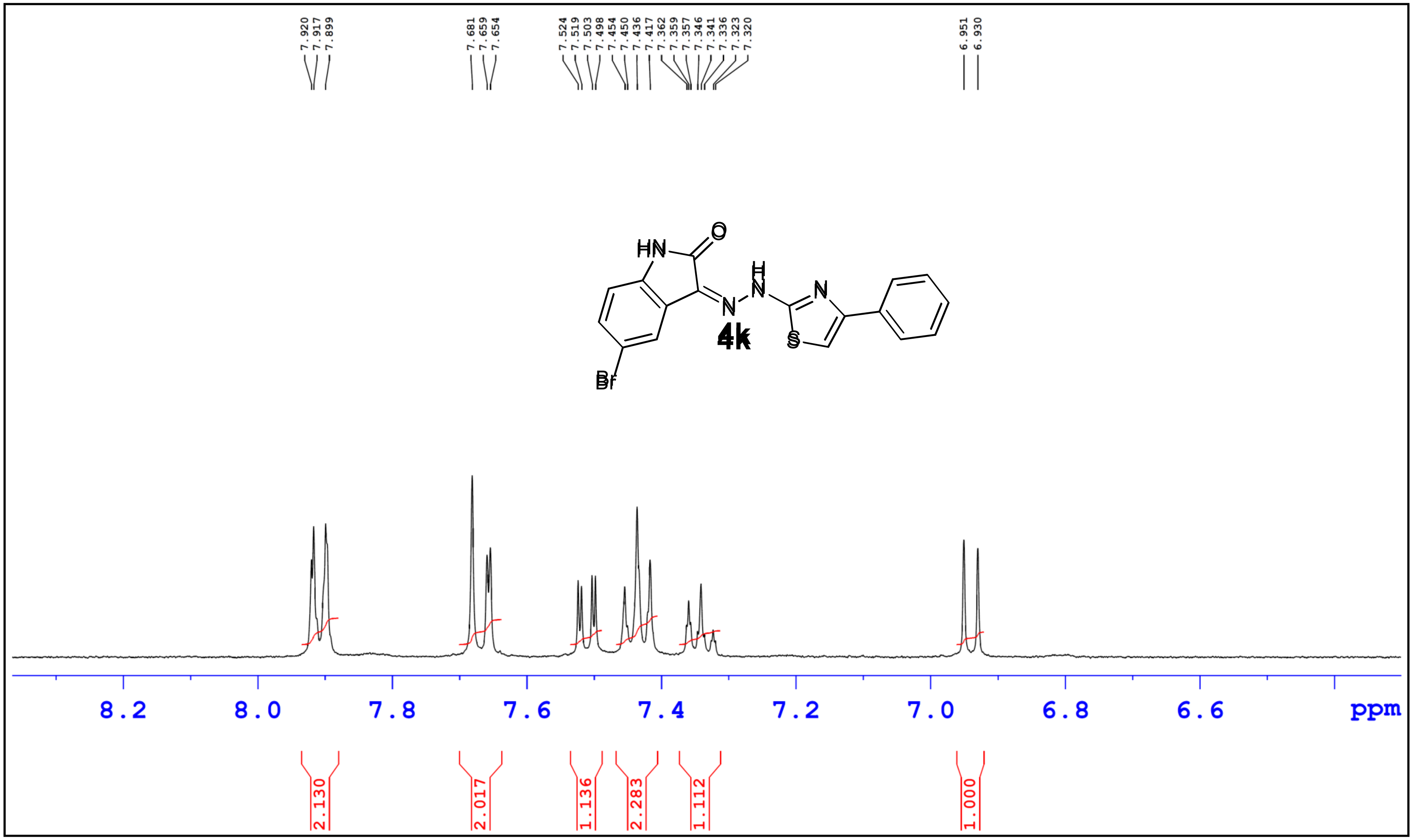

Figure S31 Expanded ^{1}H NMR spectrum of 4k.

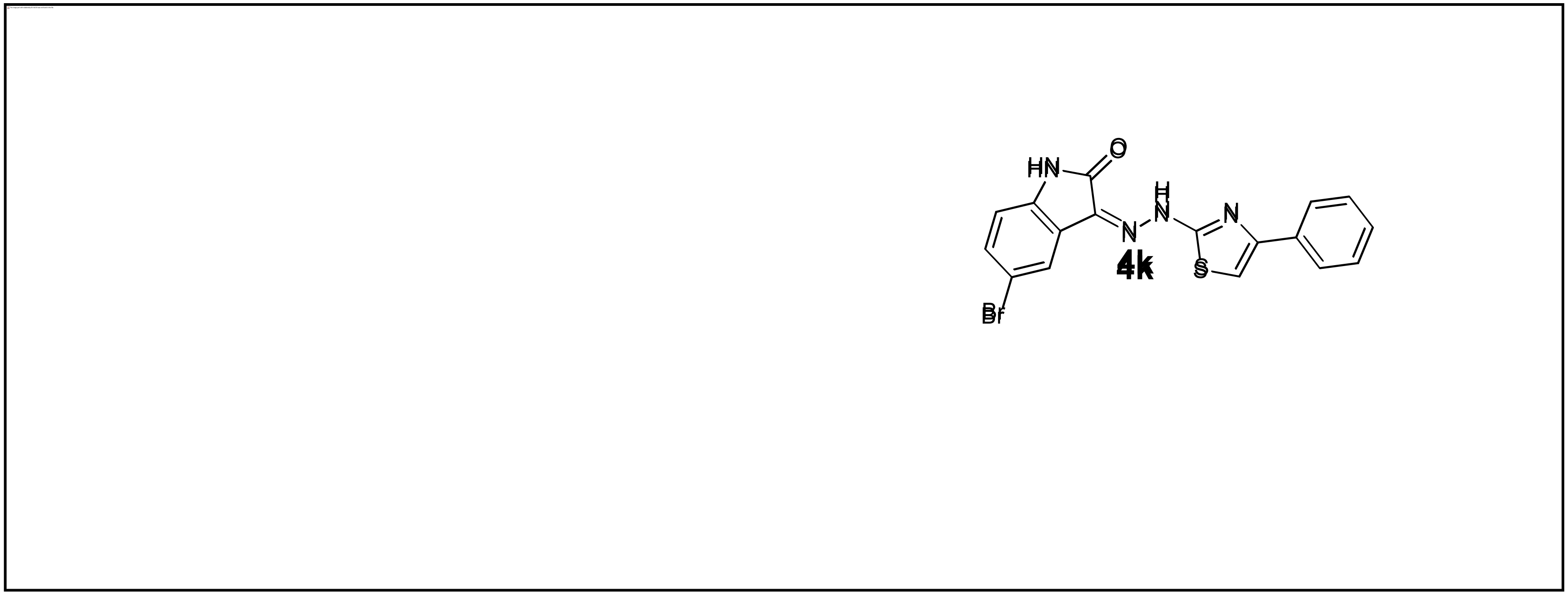

Figure S32 Mass spectrum of 4k.

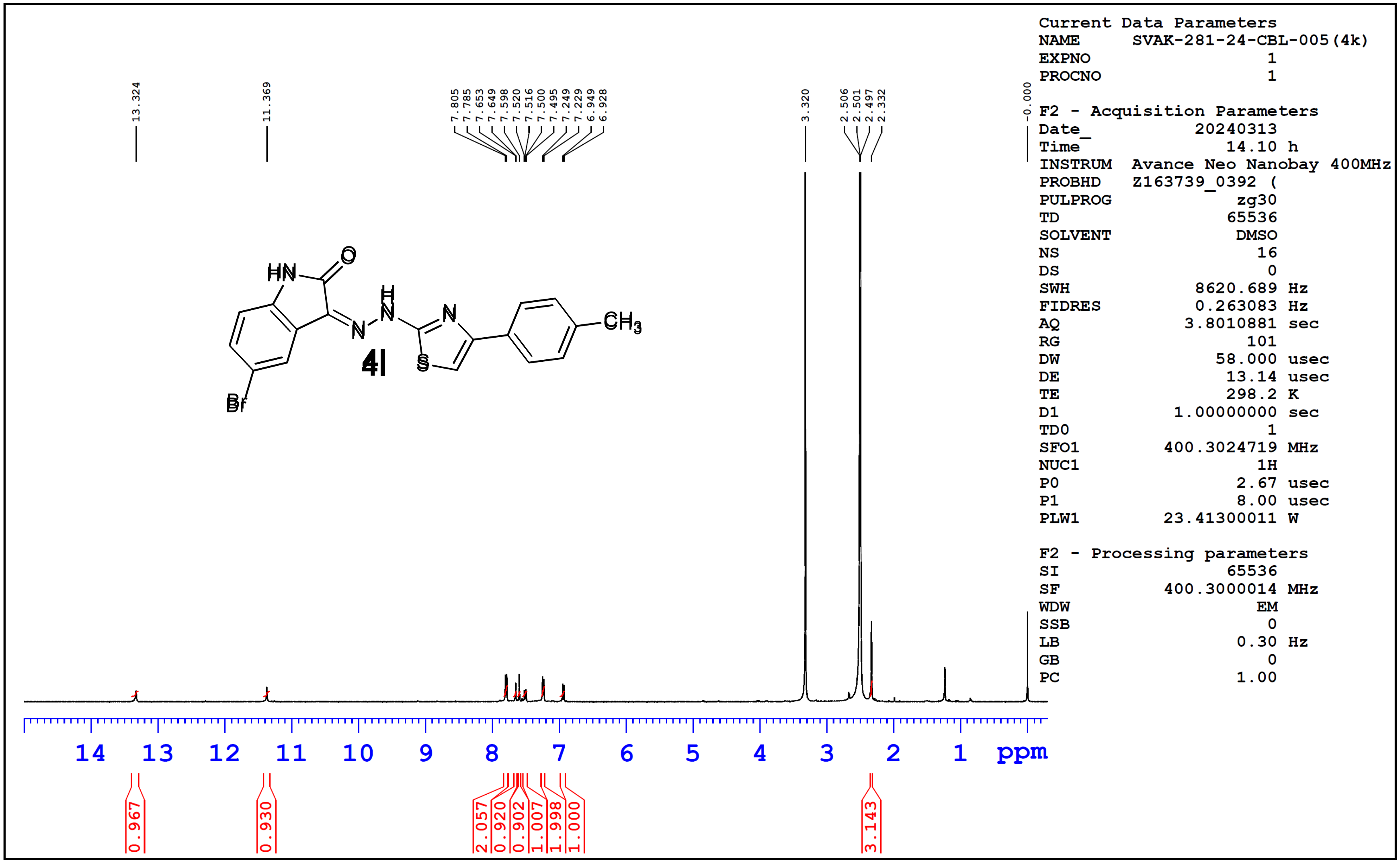

Figure S33^{1}H NMR spectrum of 4l.

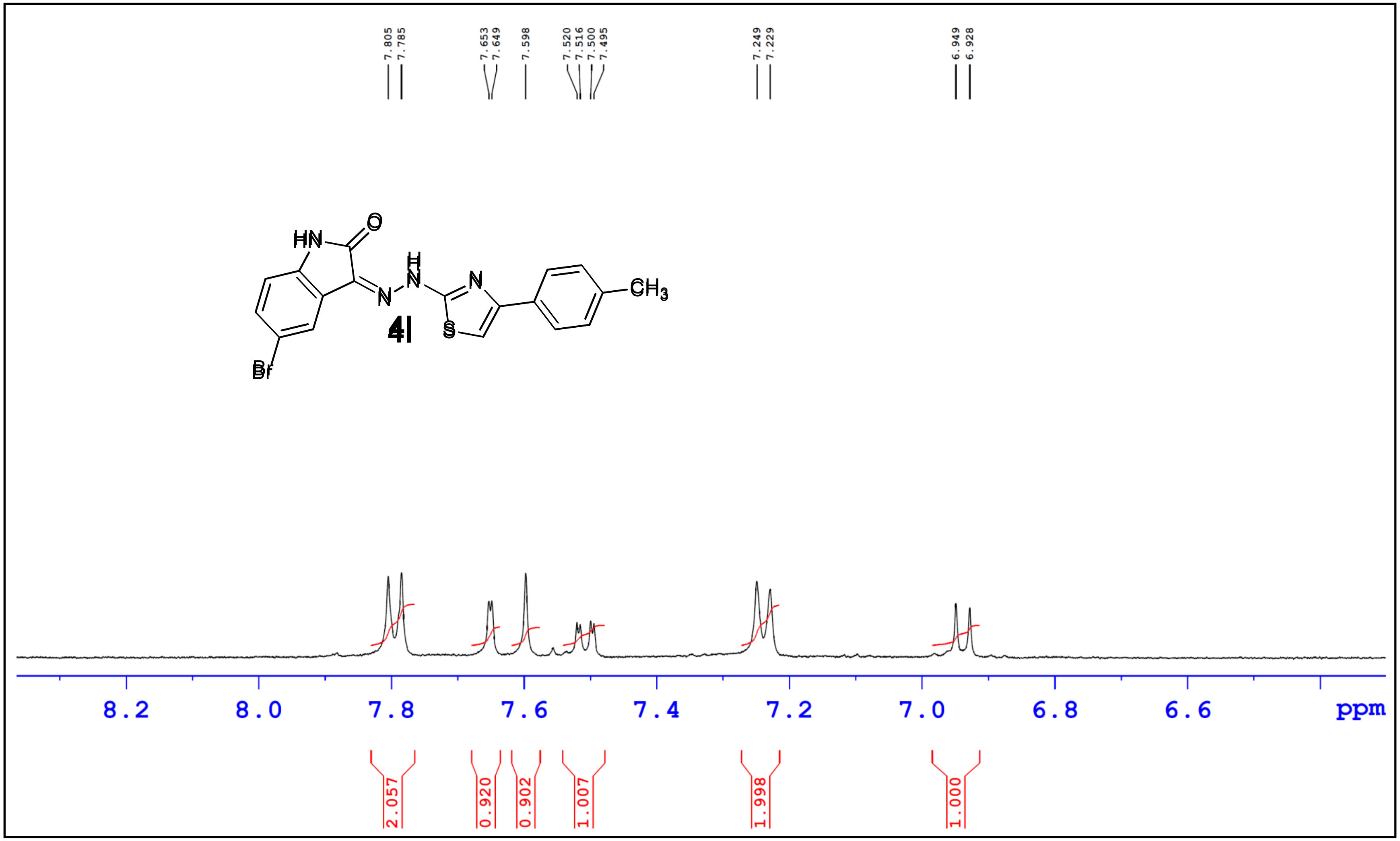

Figure S34 Expanded ^{1}H NMR spectrum of 4l.

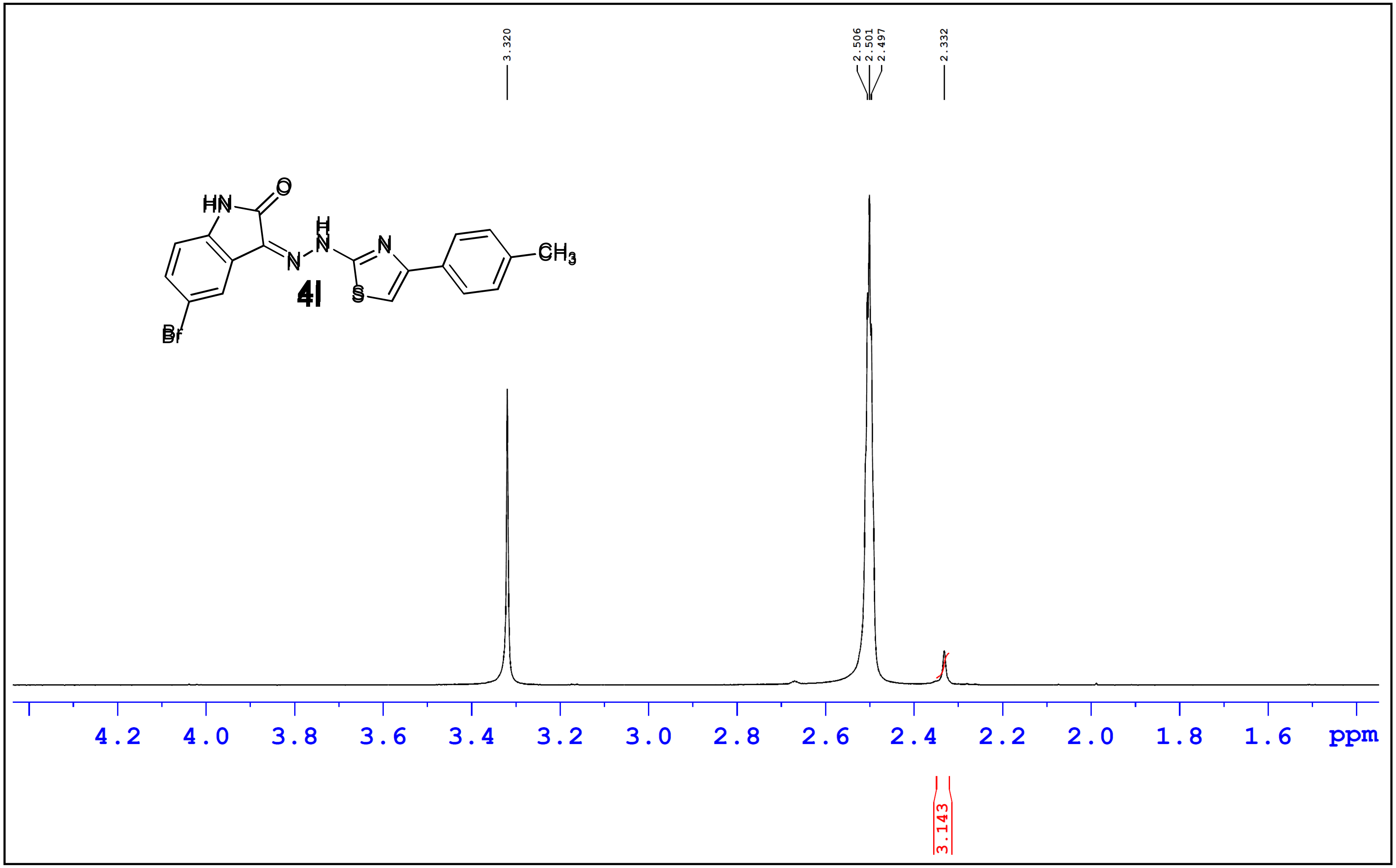

Figure S35 Expanded ^{1}H NMR spectrum of 4l.

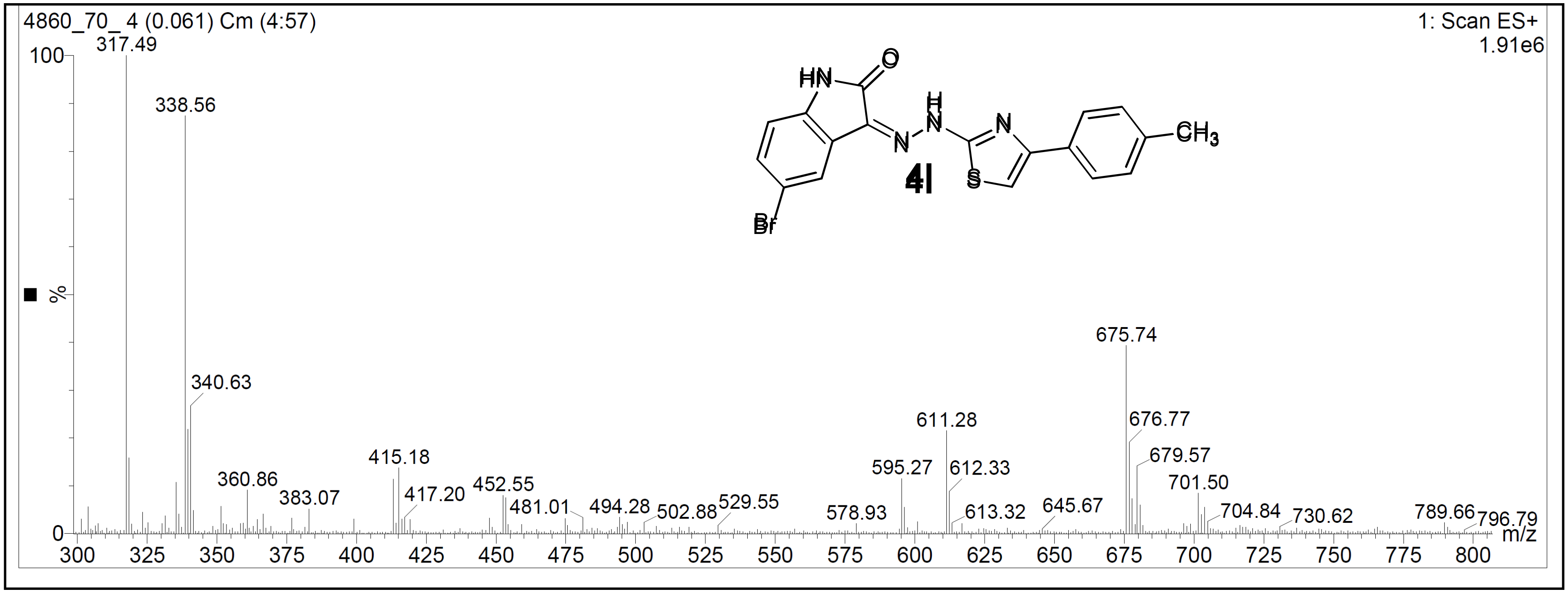

Figure S36 Mass spectrum of 4l.

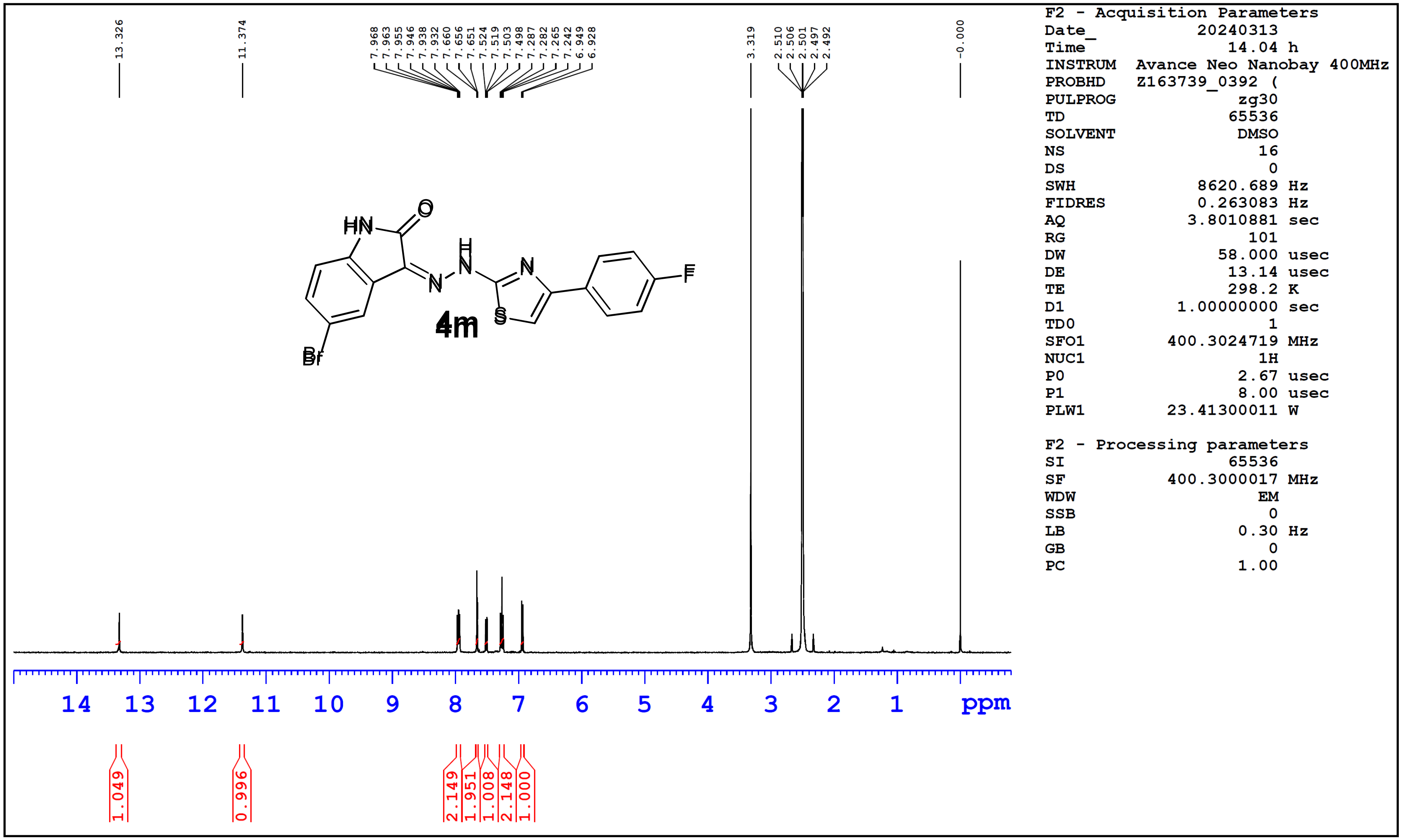

Figure S37^{1}H NMR spectrum of 4m.

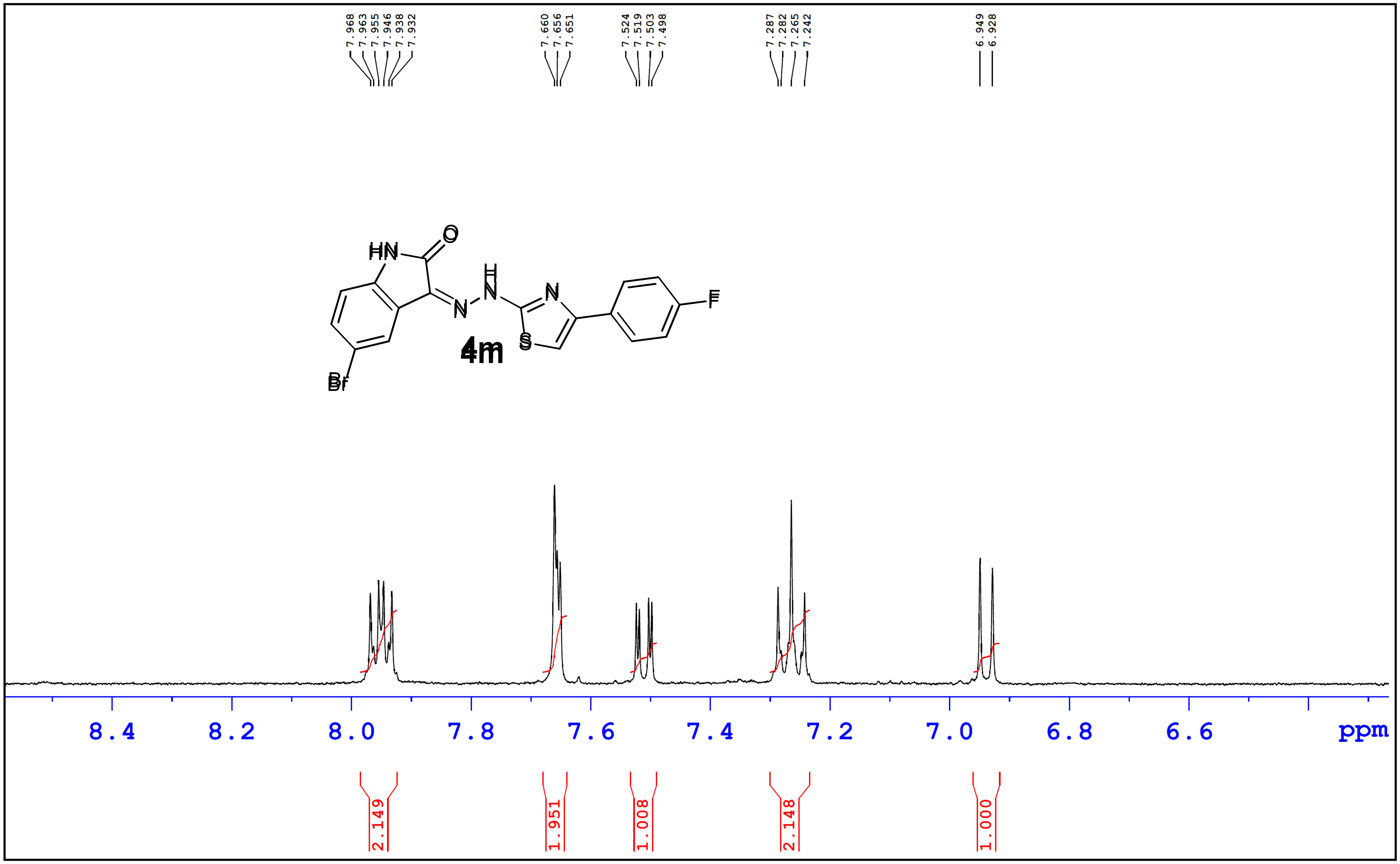

Figure S38 Expanded ^{1}H NMR spectrum of 4m.

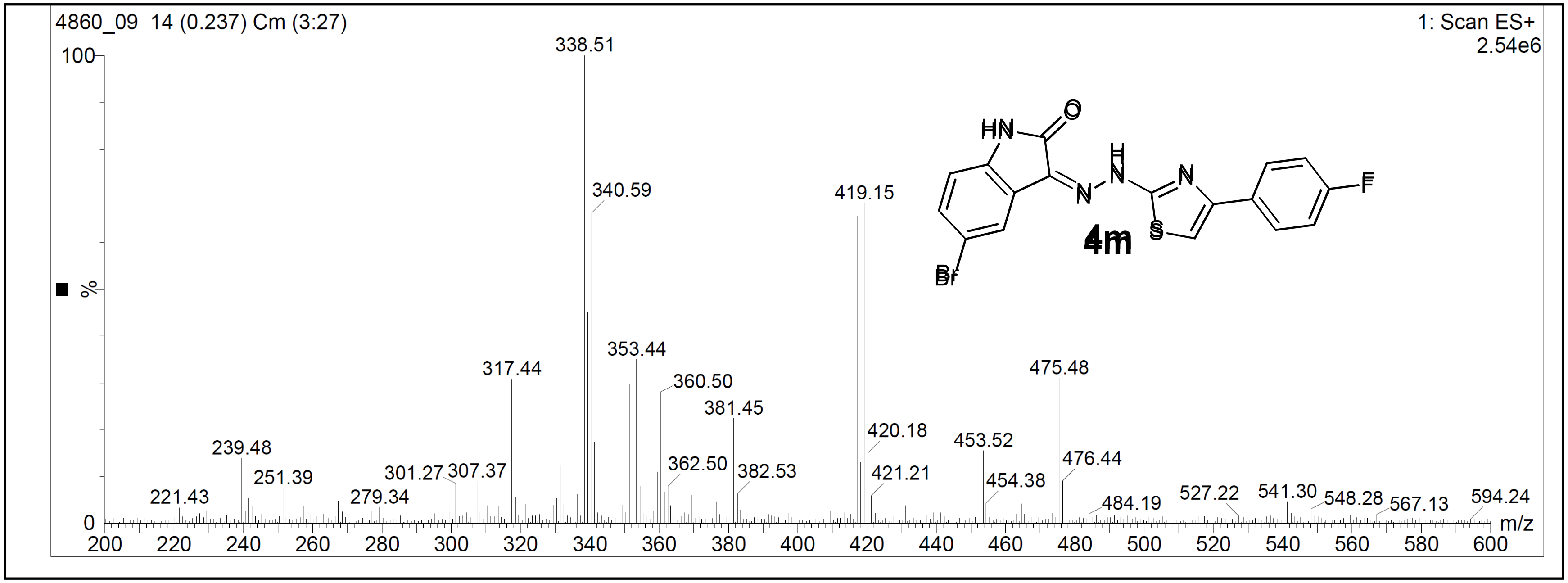

Figure S39 Mass spectrum of 4m.

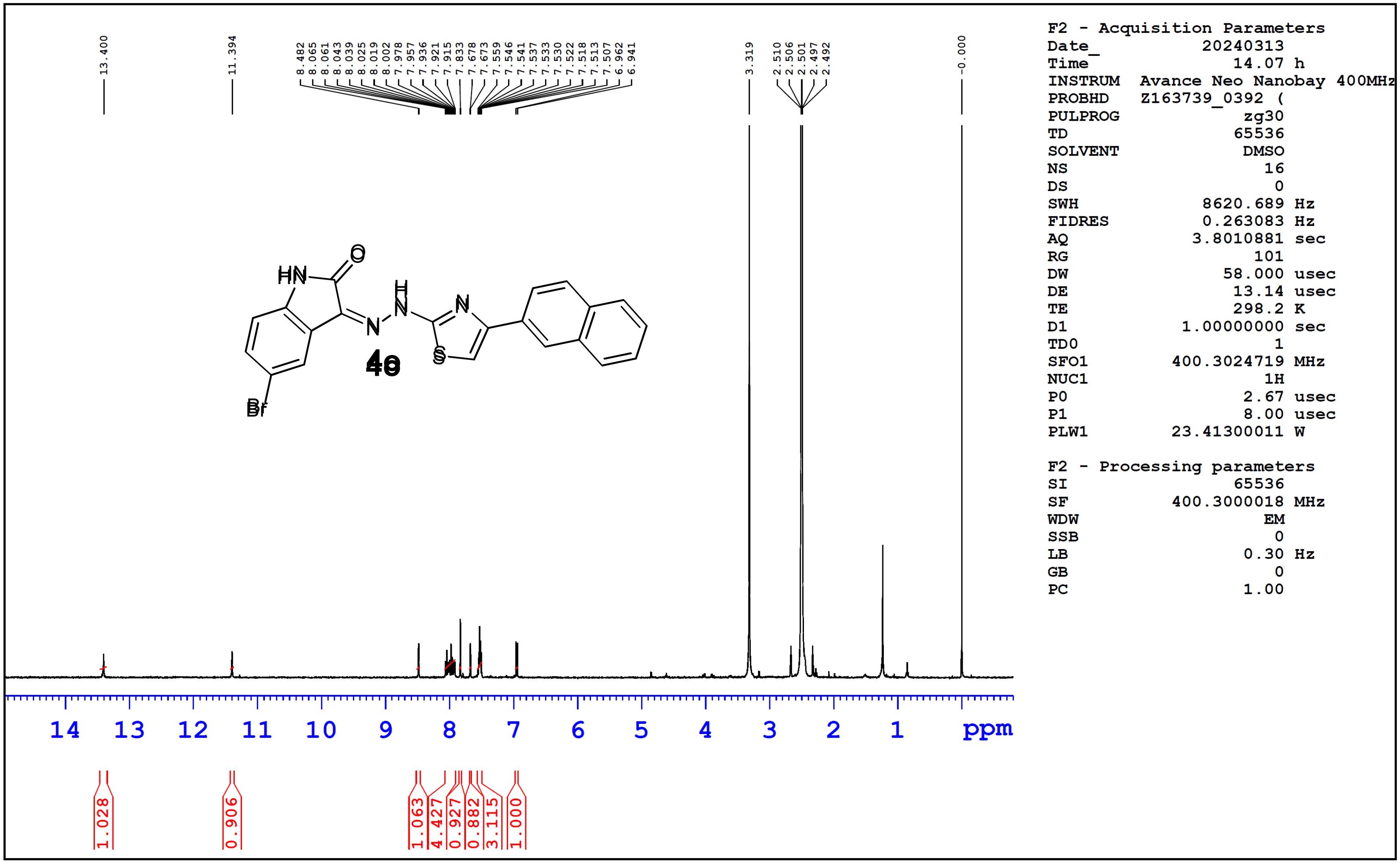

Figure S40 ¹H NMR spectrum of 4o.

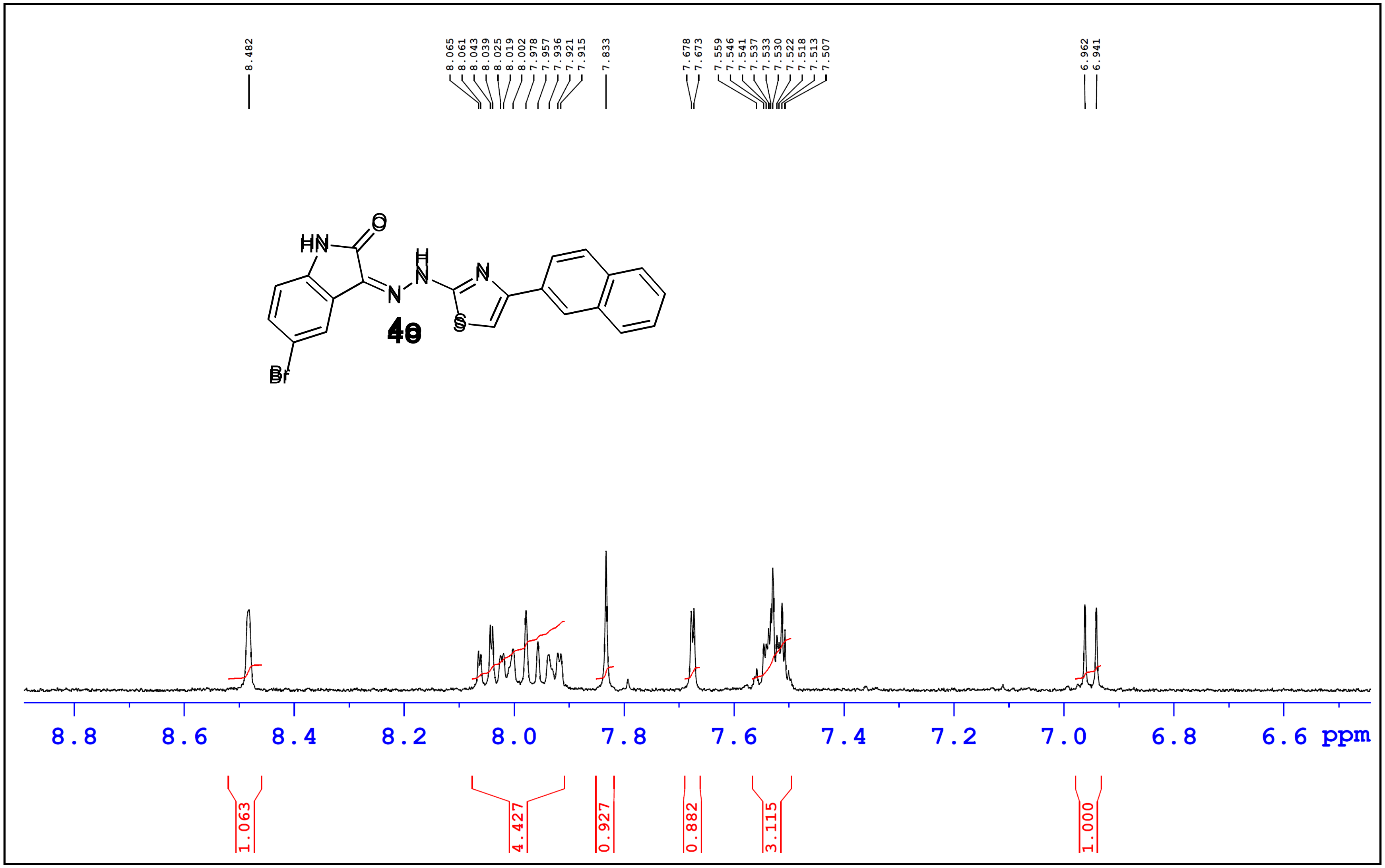

Figure S41 Expanded ^{1}H NMR spectrum of 4o.

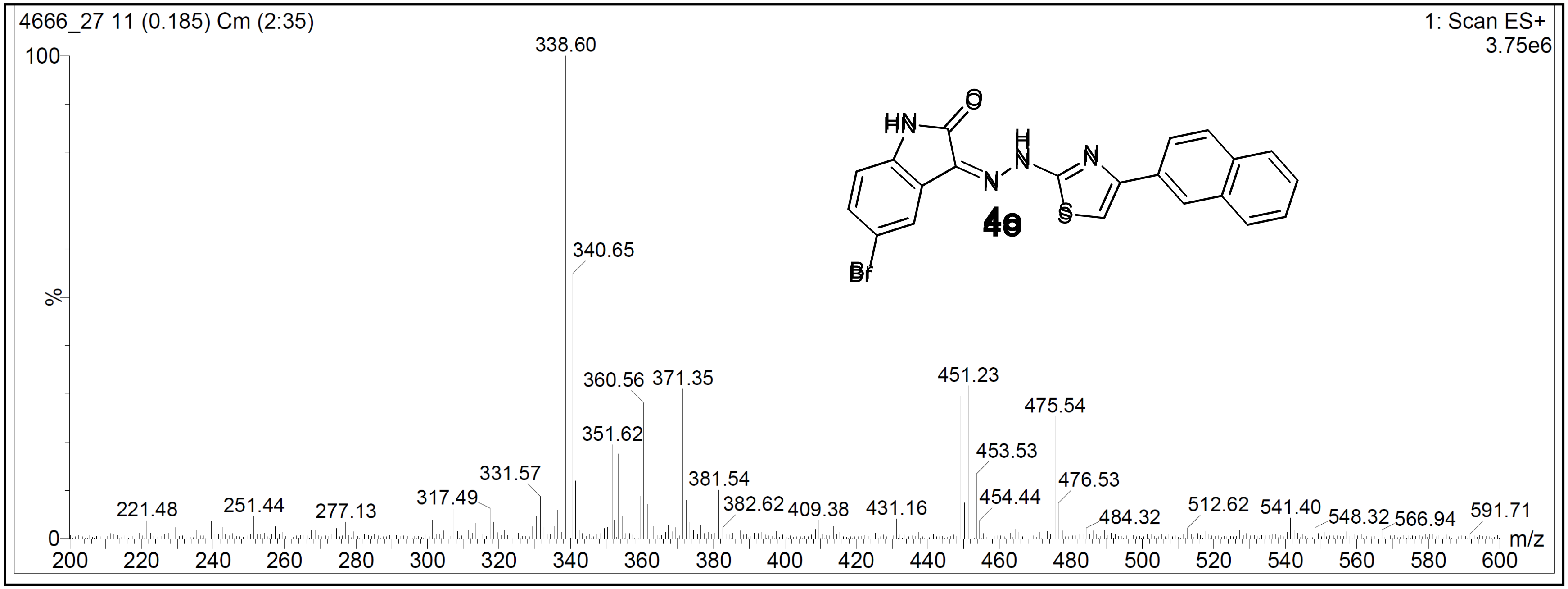

Figure S42 Mass spectrum of 4o.

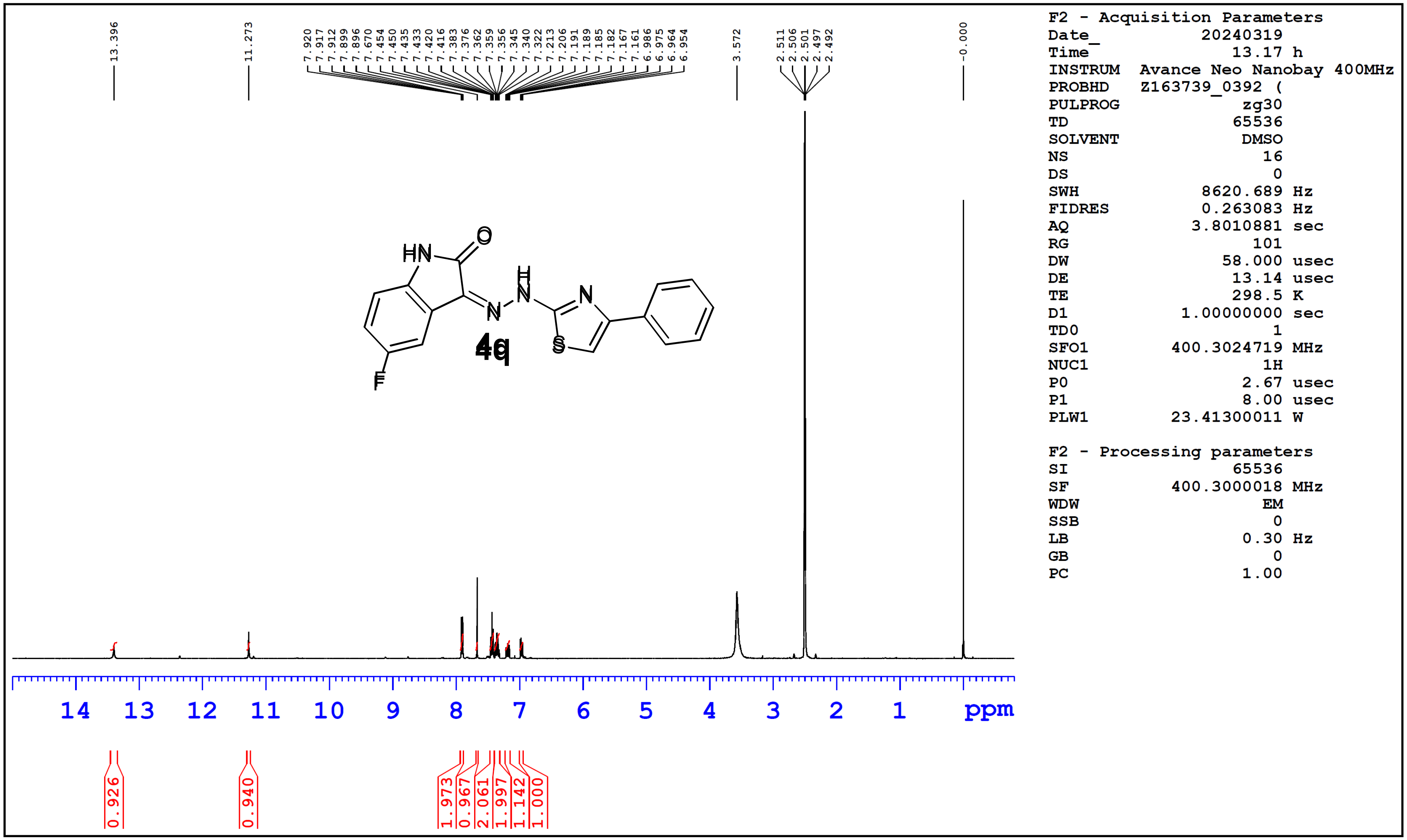

Figure S43^{1}H NMR spectrum of 4q.

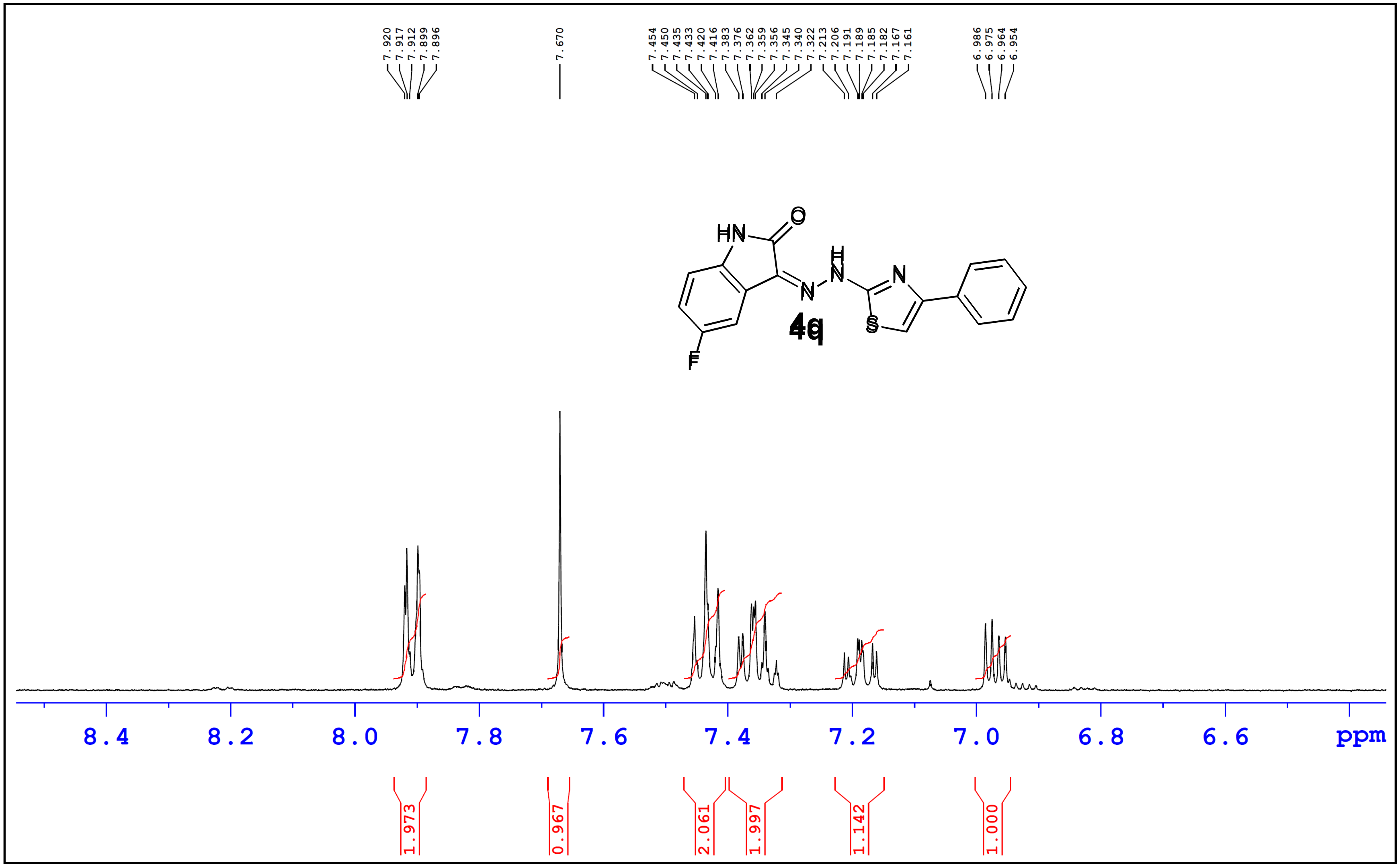

Figure S44 Expanded ^{1}H NMR spectrum of 4q.

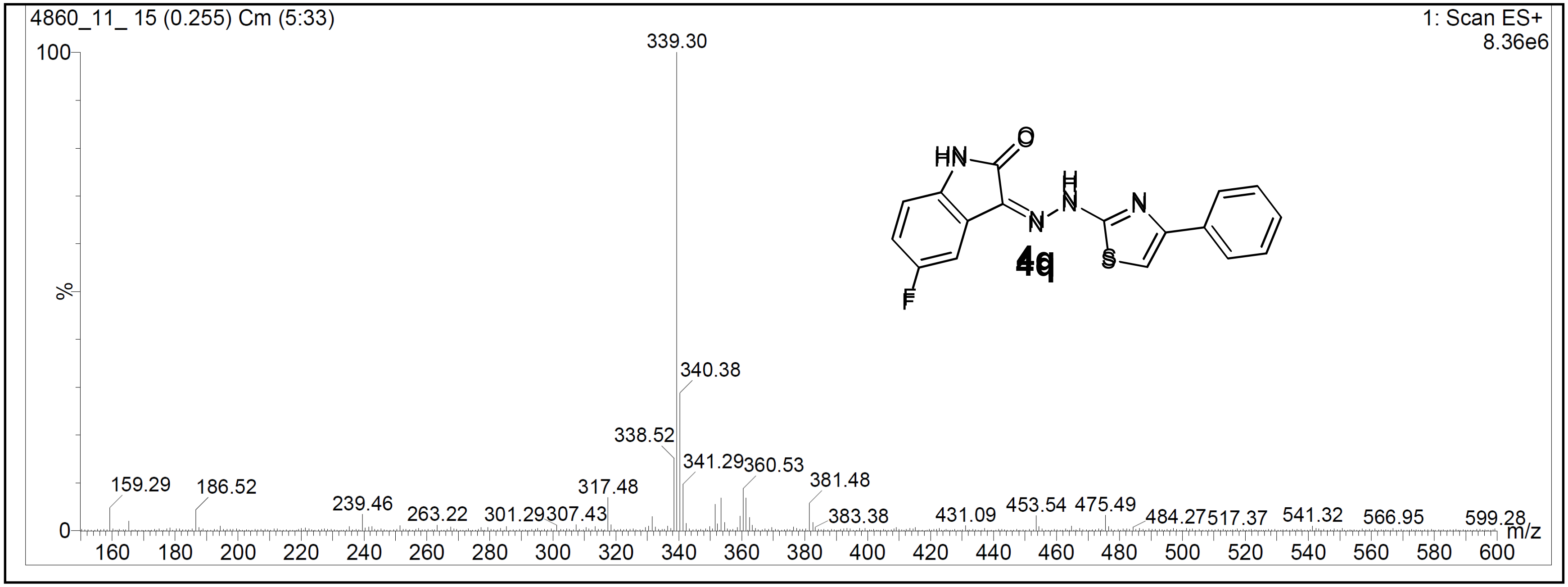

Figure S45 Mass spectrum of 4q.

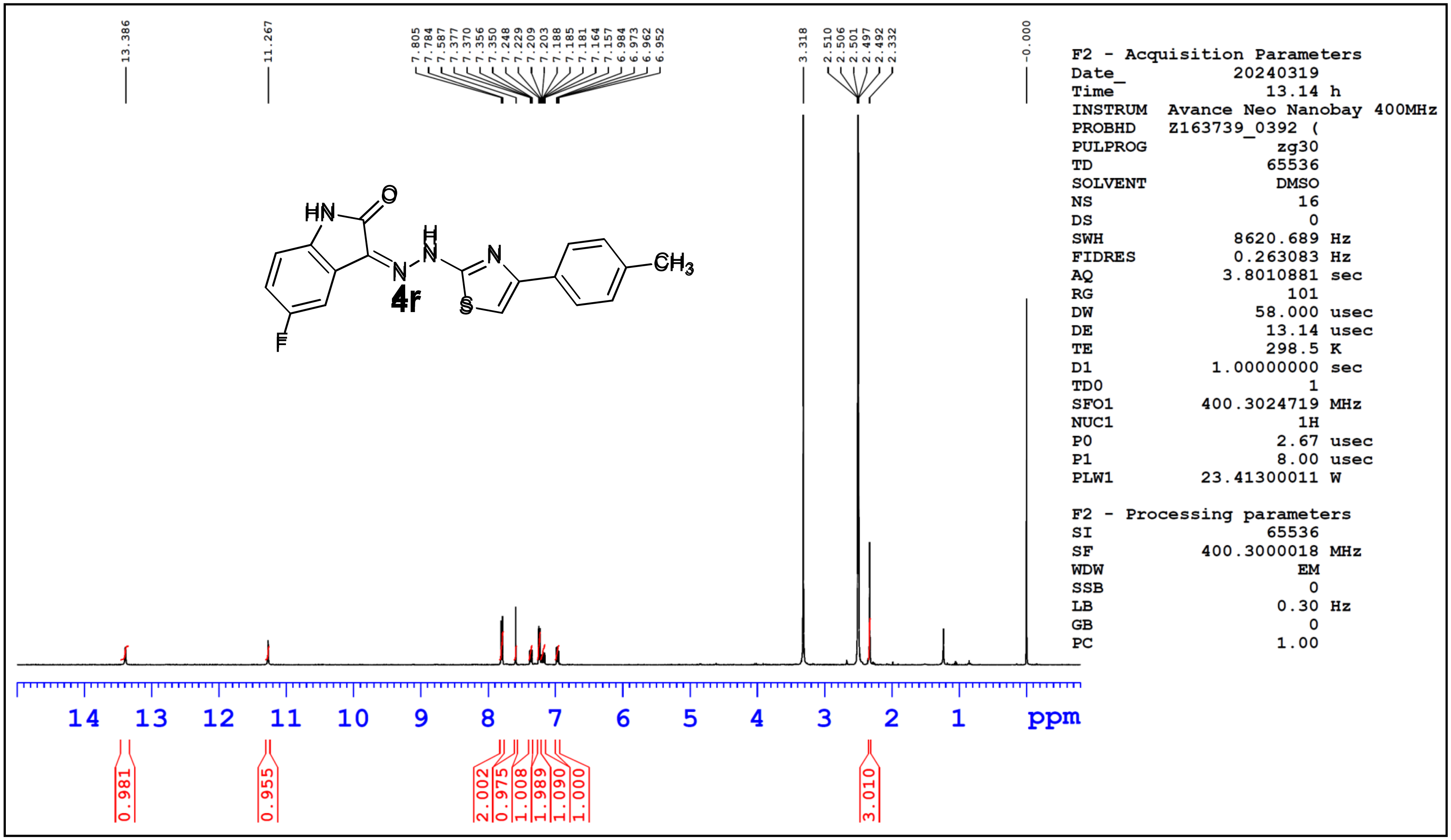

Figure S46 ^{1}H NMR spectrum of 4r.

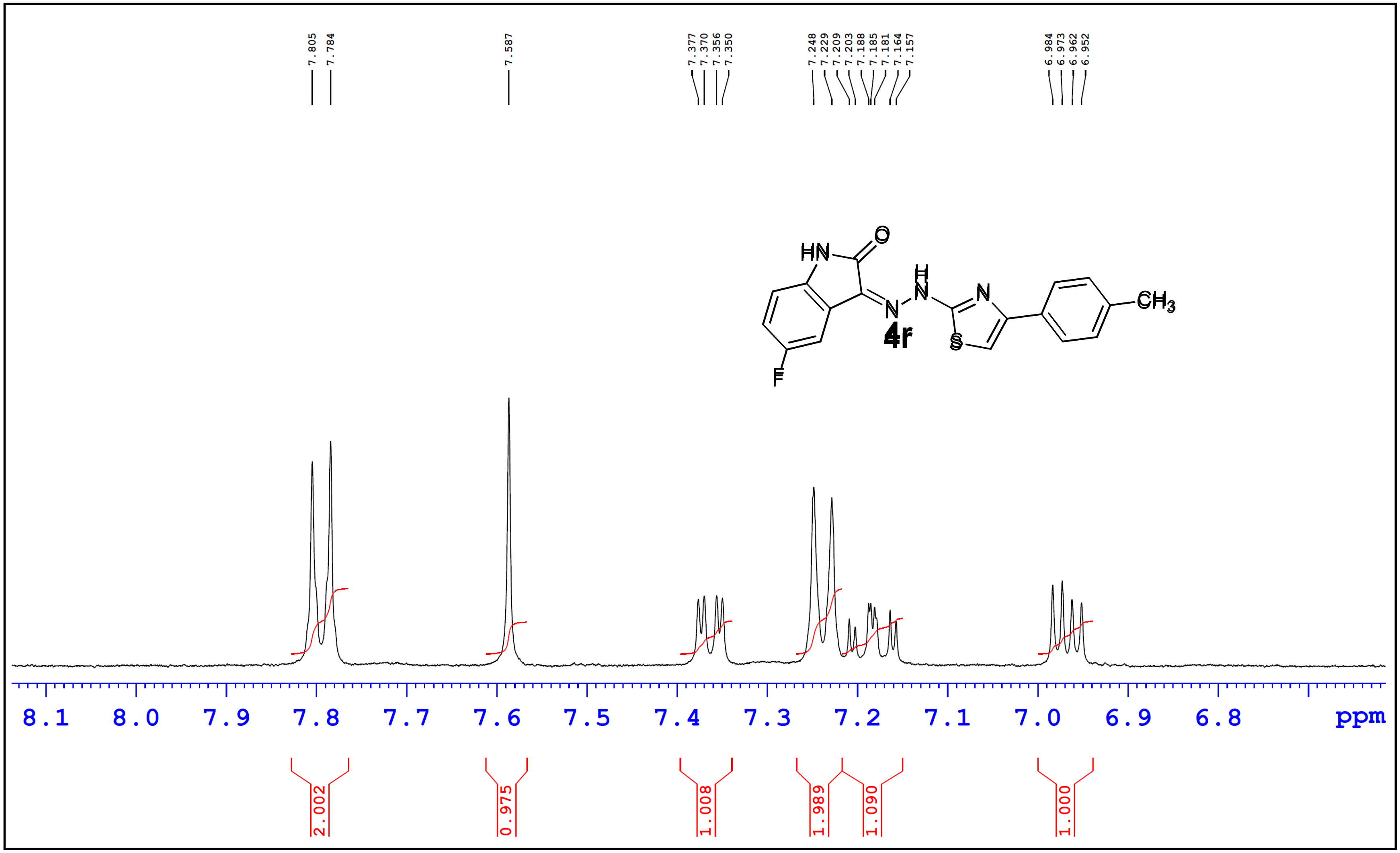

Figure S47 Expanded ^{1}H NMR spectrum of 4r.

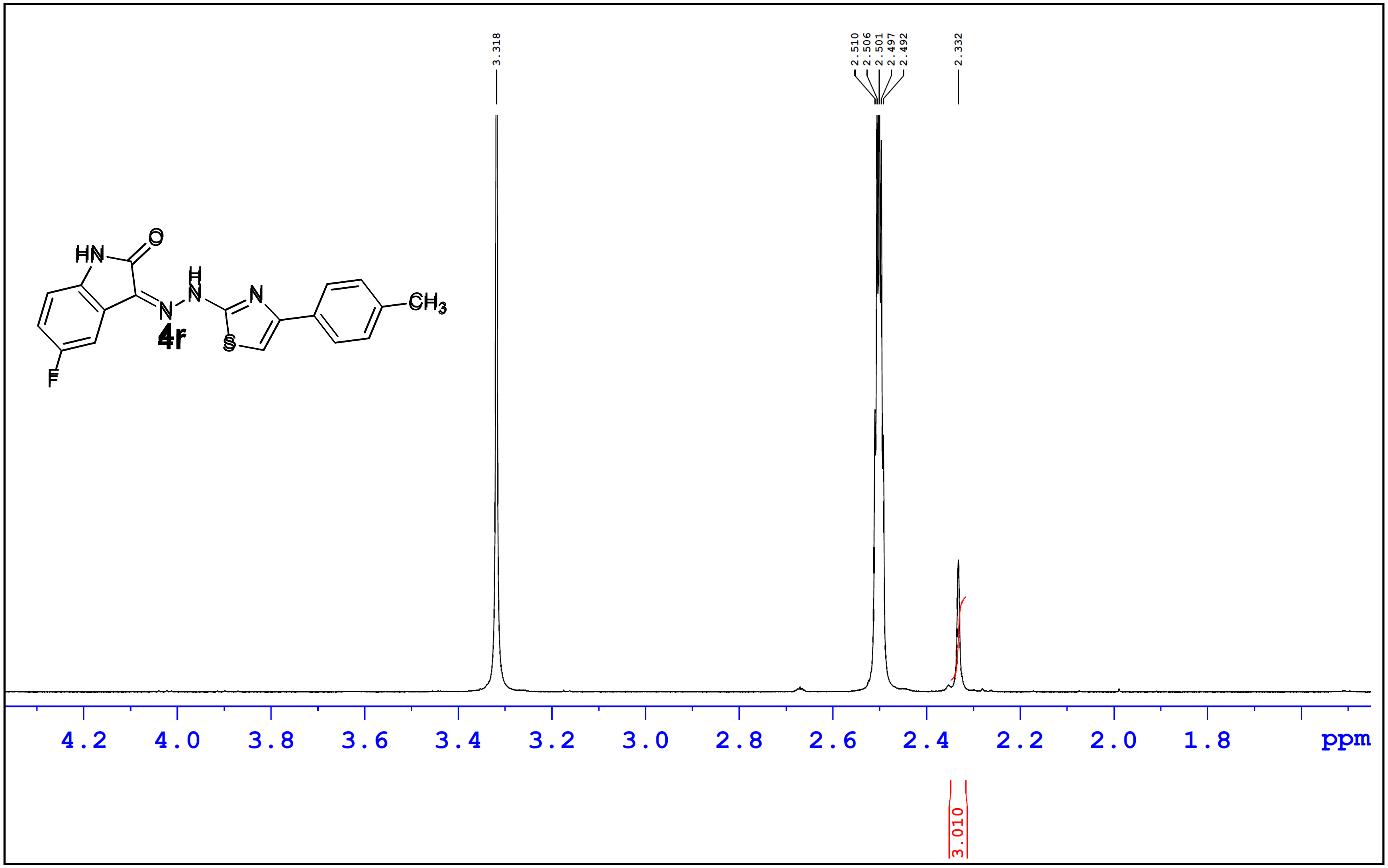

Figure S48 Expanded ^{1}H NMR spectrum of 4r.

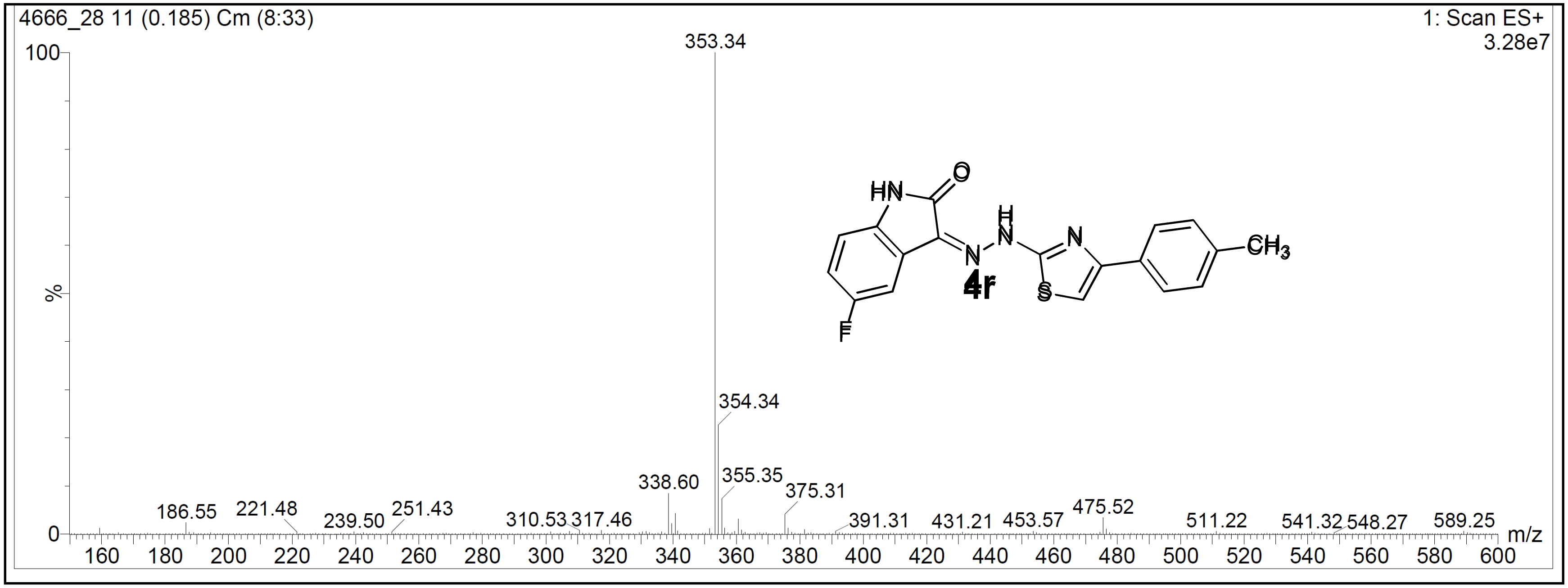

Figure S49 Mass spectrum of 4r.

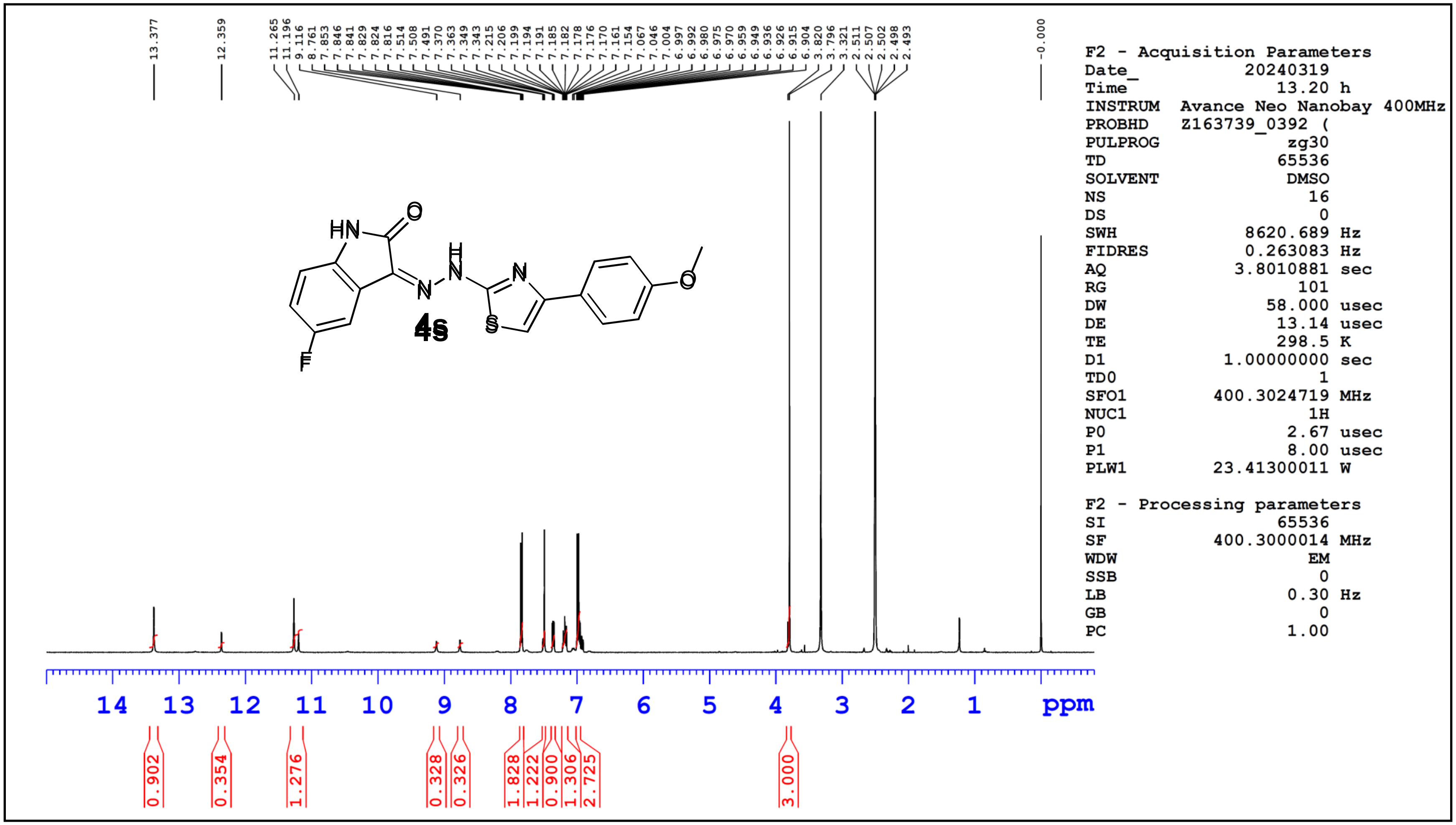

Figure S50^{1}H NMR spectrum of 4s.

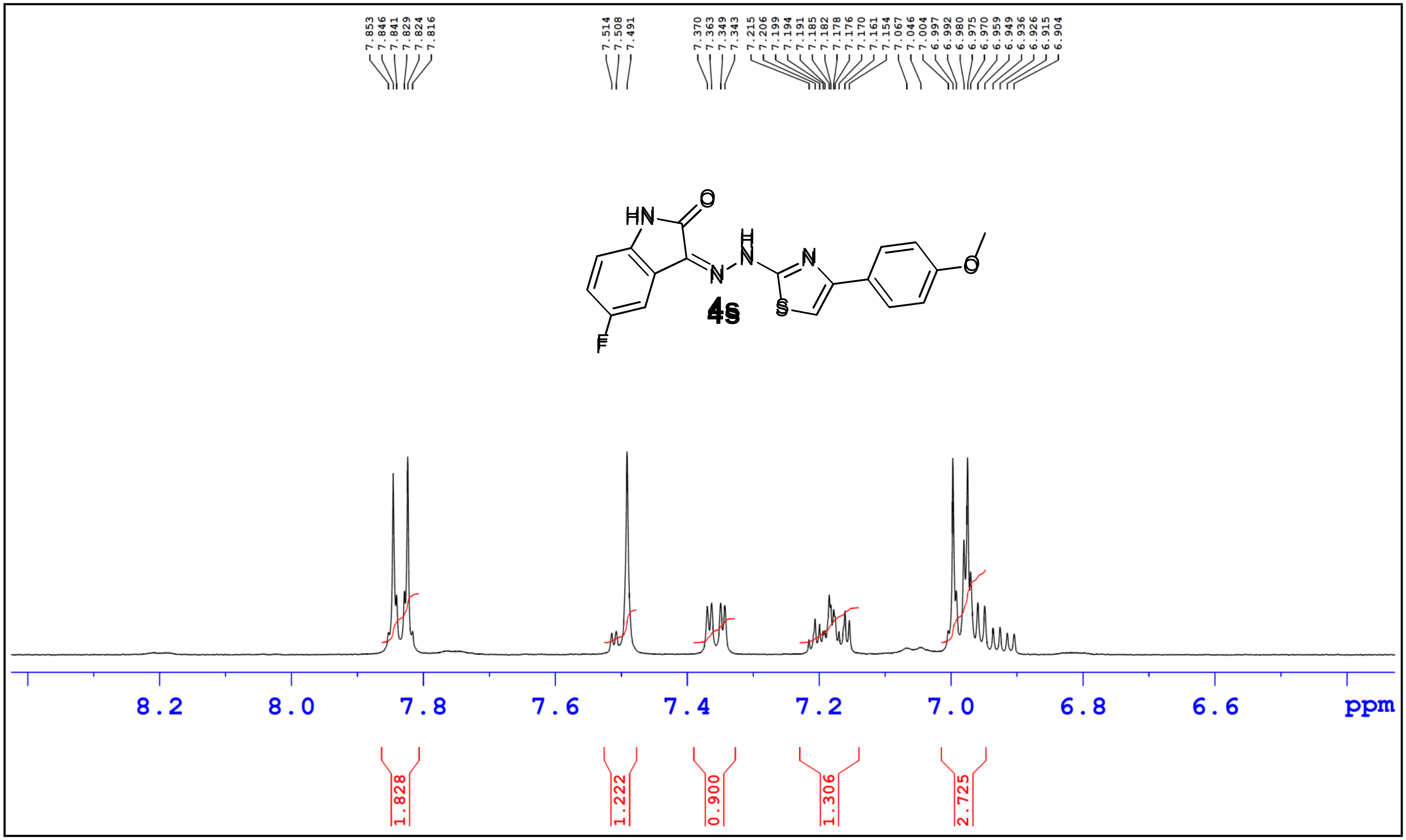

Figure S51 Expanded ^{1}H NMR spectrum of 4s.

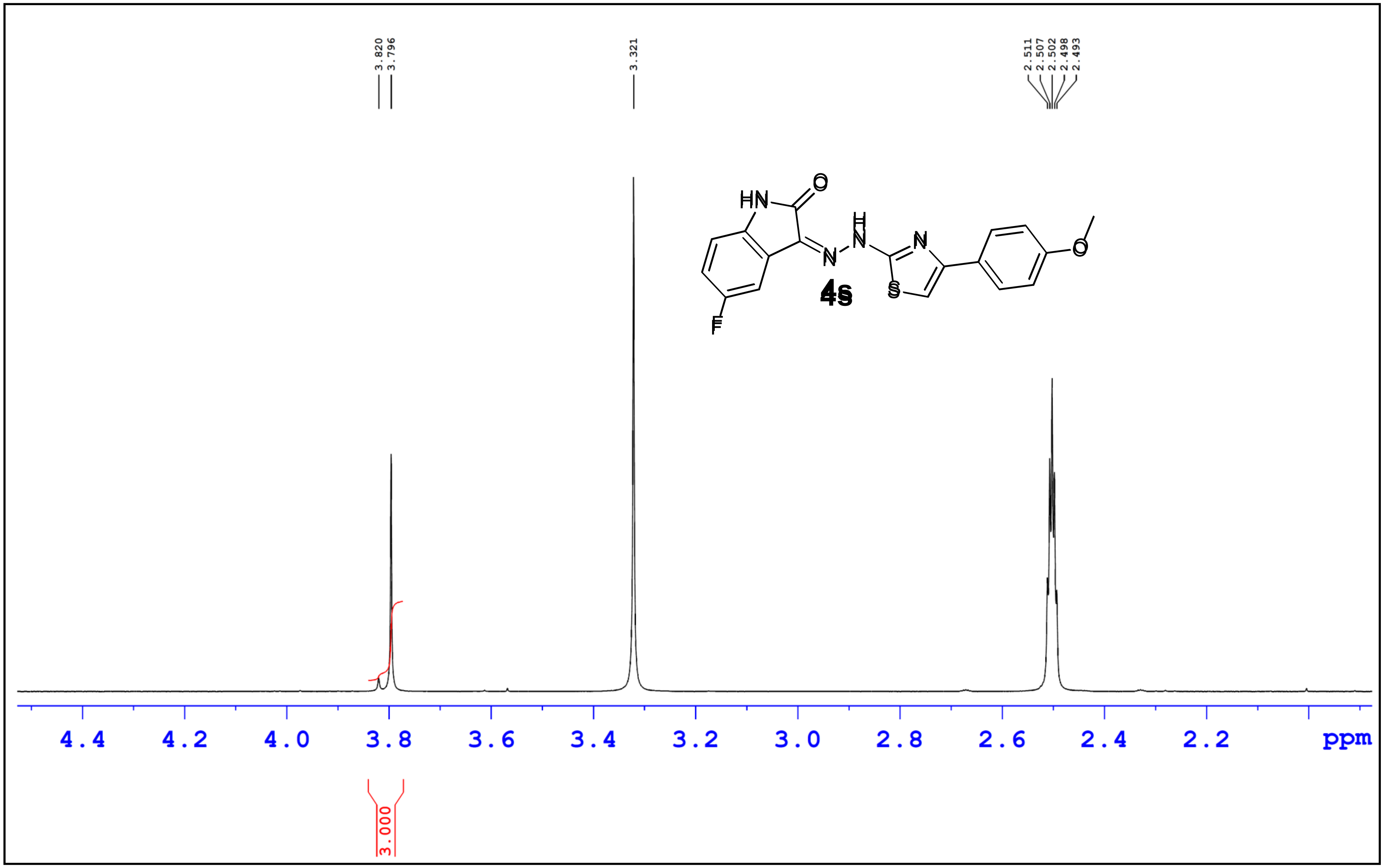

Figure S52 Expanded ^{1}H NMR spectrum of 4s.

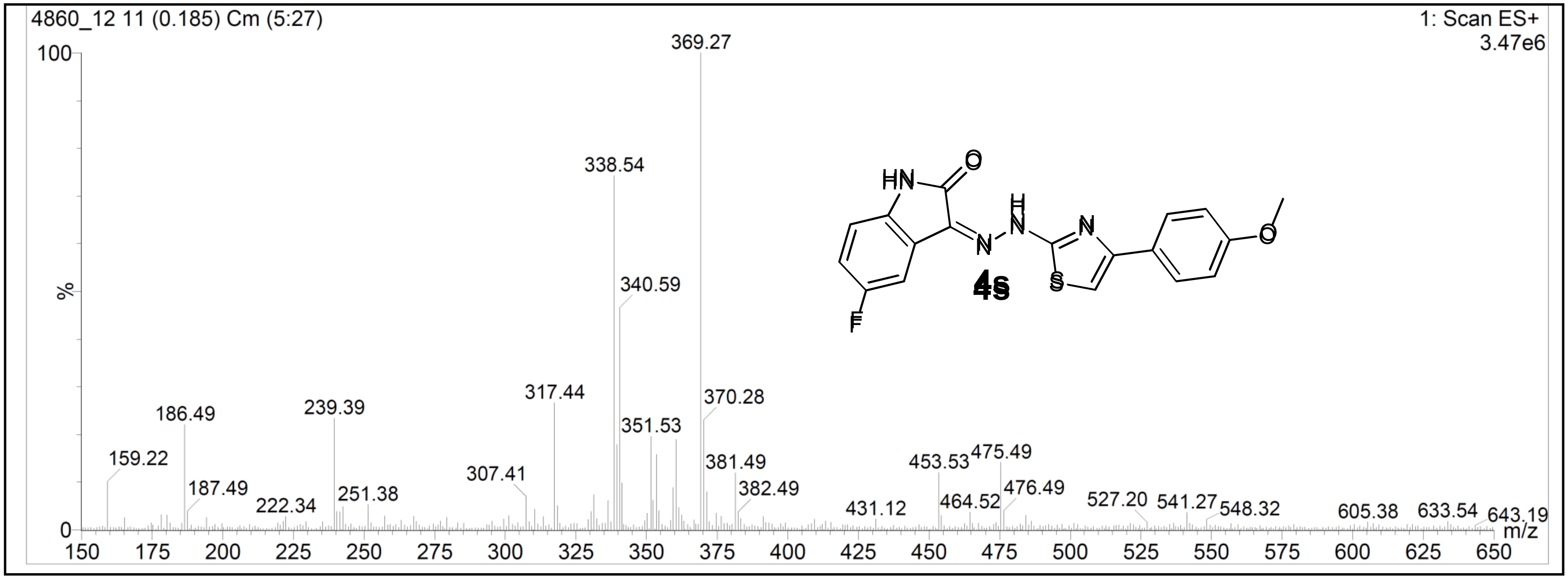

Figure S53 Mass spectrum of 4s.

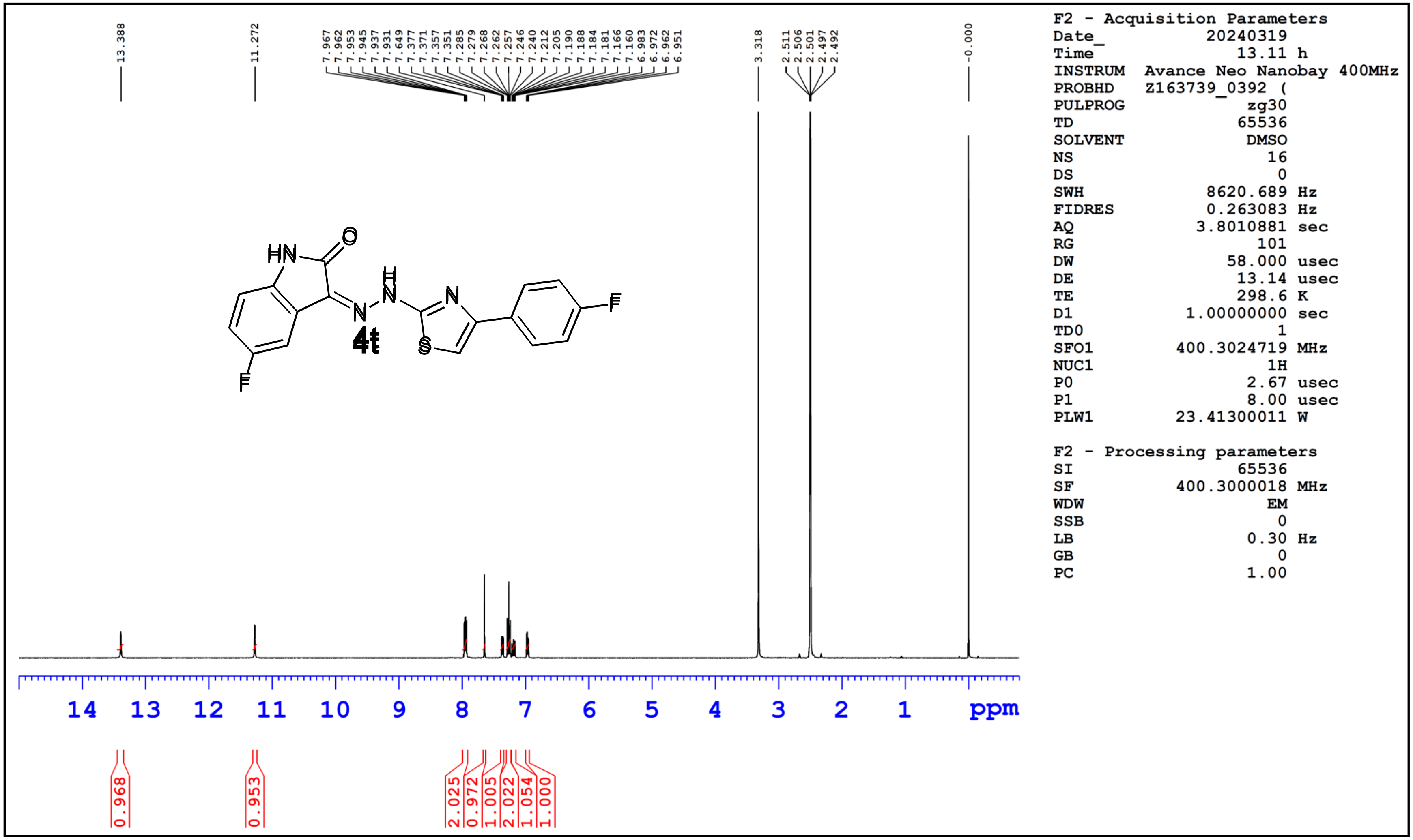

Figure S54 ^{1}H NMR spectrum of 4t.

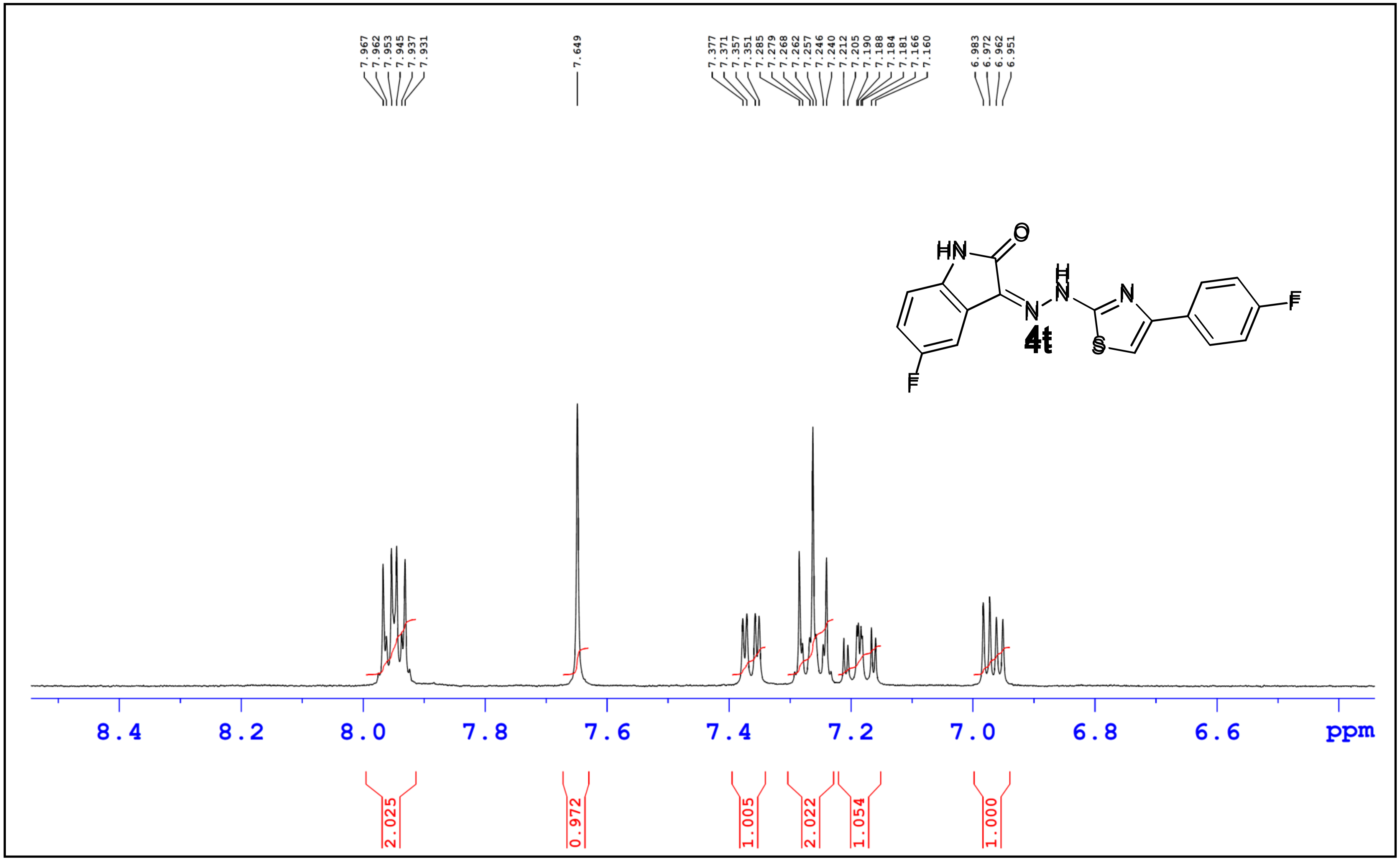

Figure S55 Expanded ^{1}H NMR spectrum of 4t.

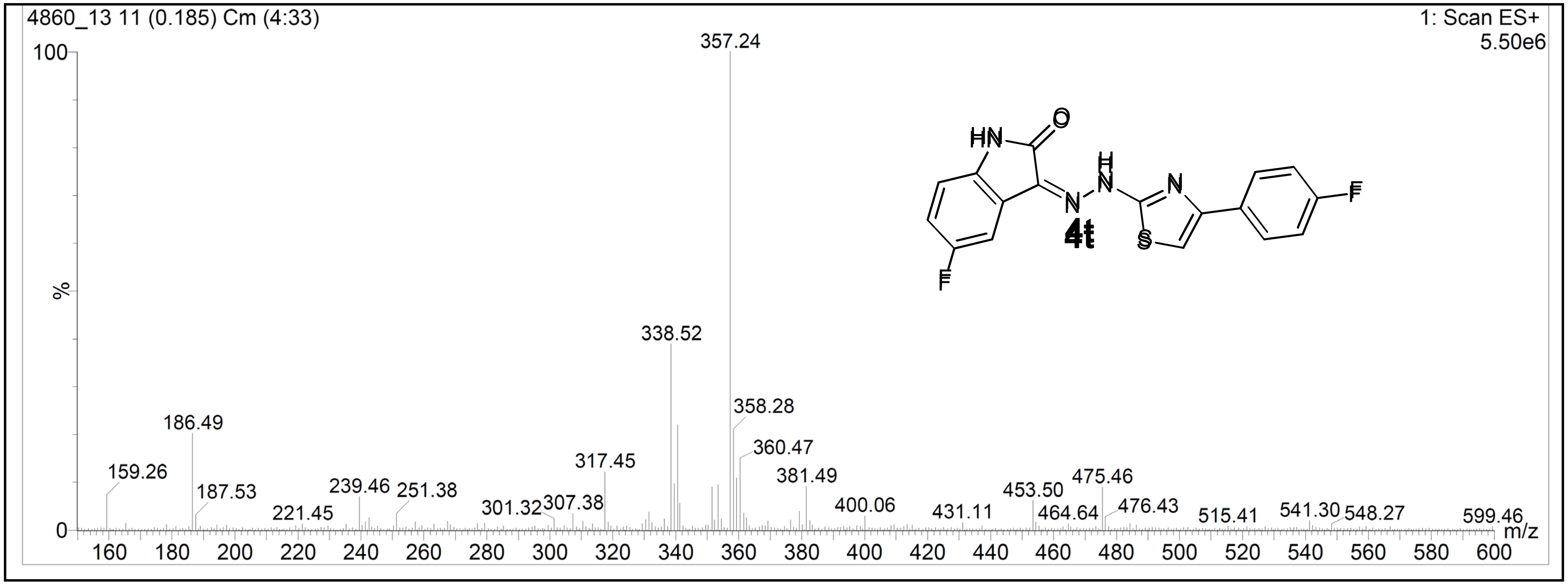

Figure S56 Mass spectrum of 4t.

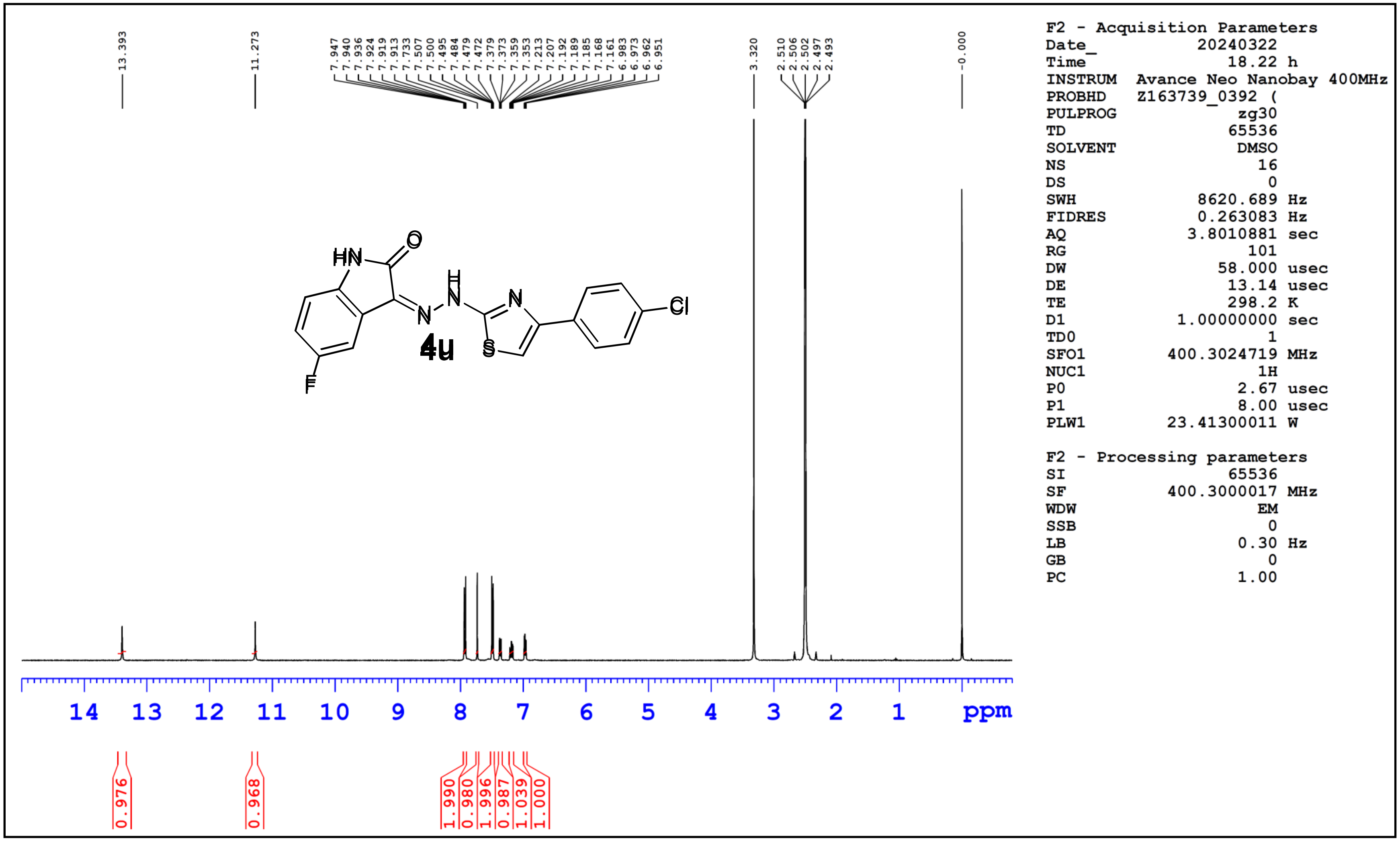

Figure S57^{1}H NMR spectrum of 4u.

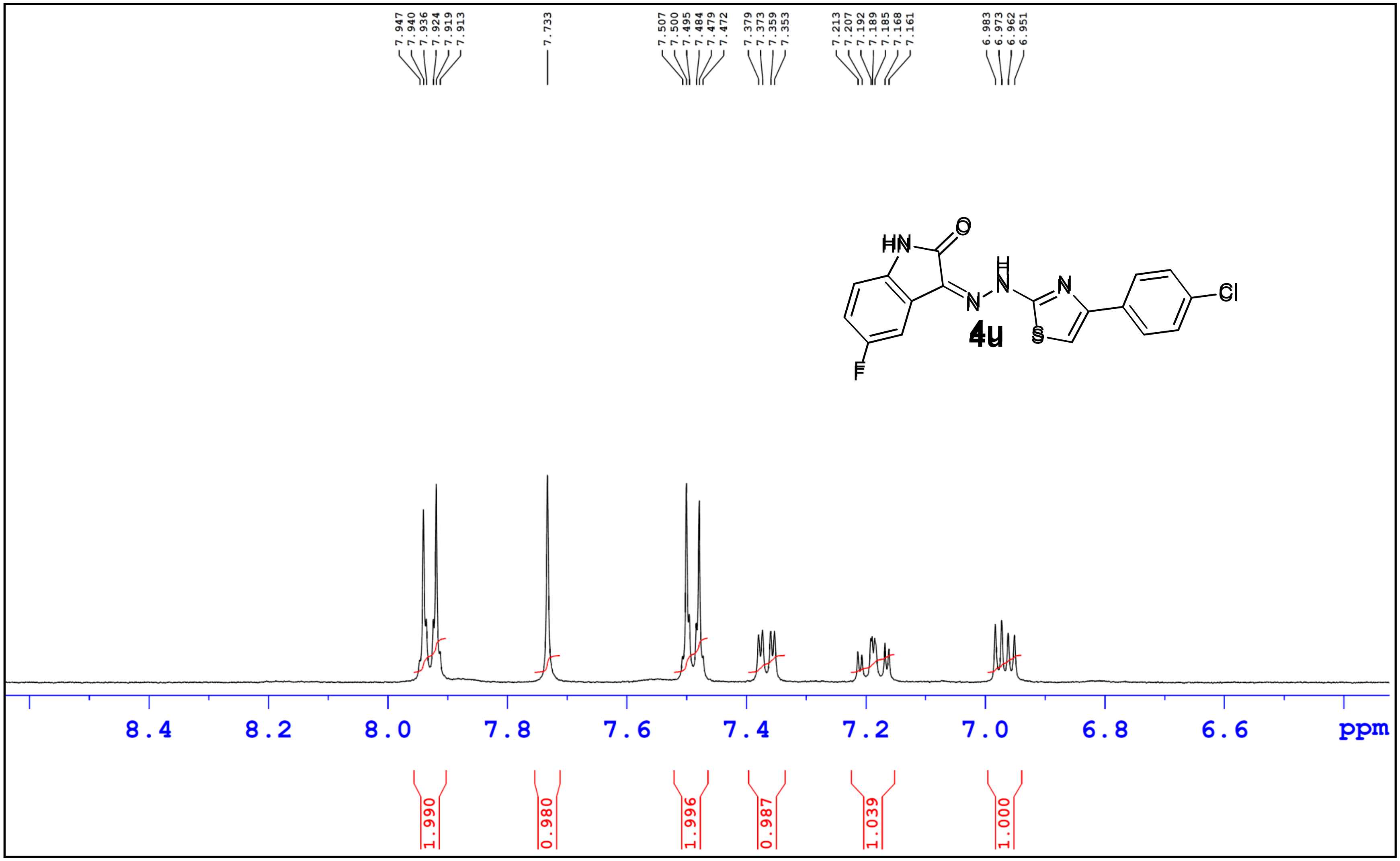

Figure S58 Expanded ^{1}H NMR spectrum of 4u.

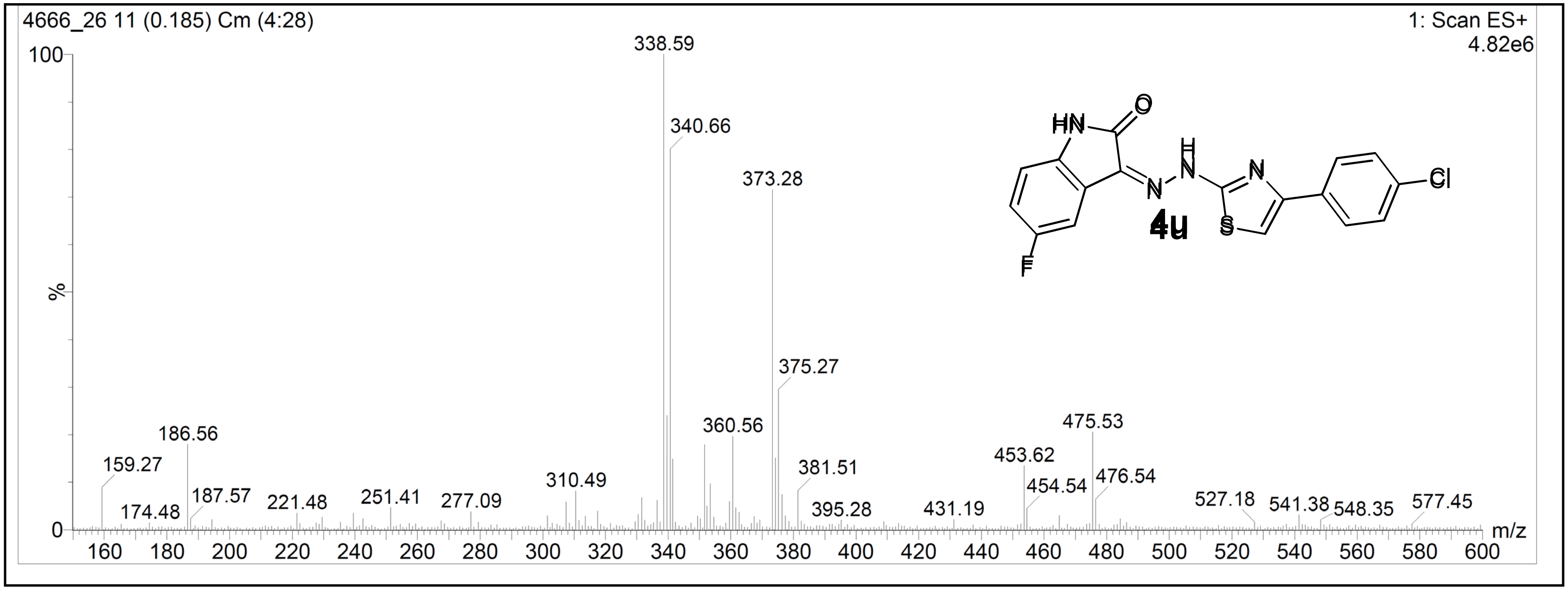

Figure S59 Mass spectrum of 4u.

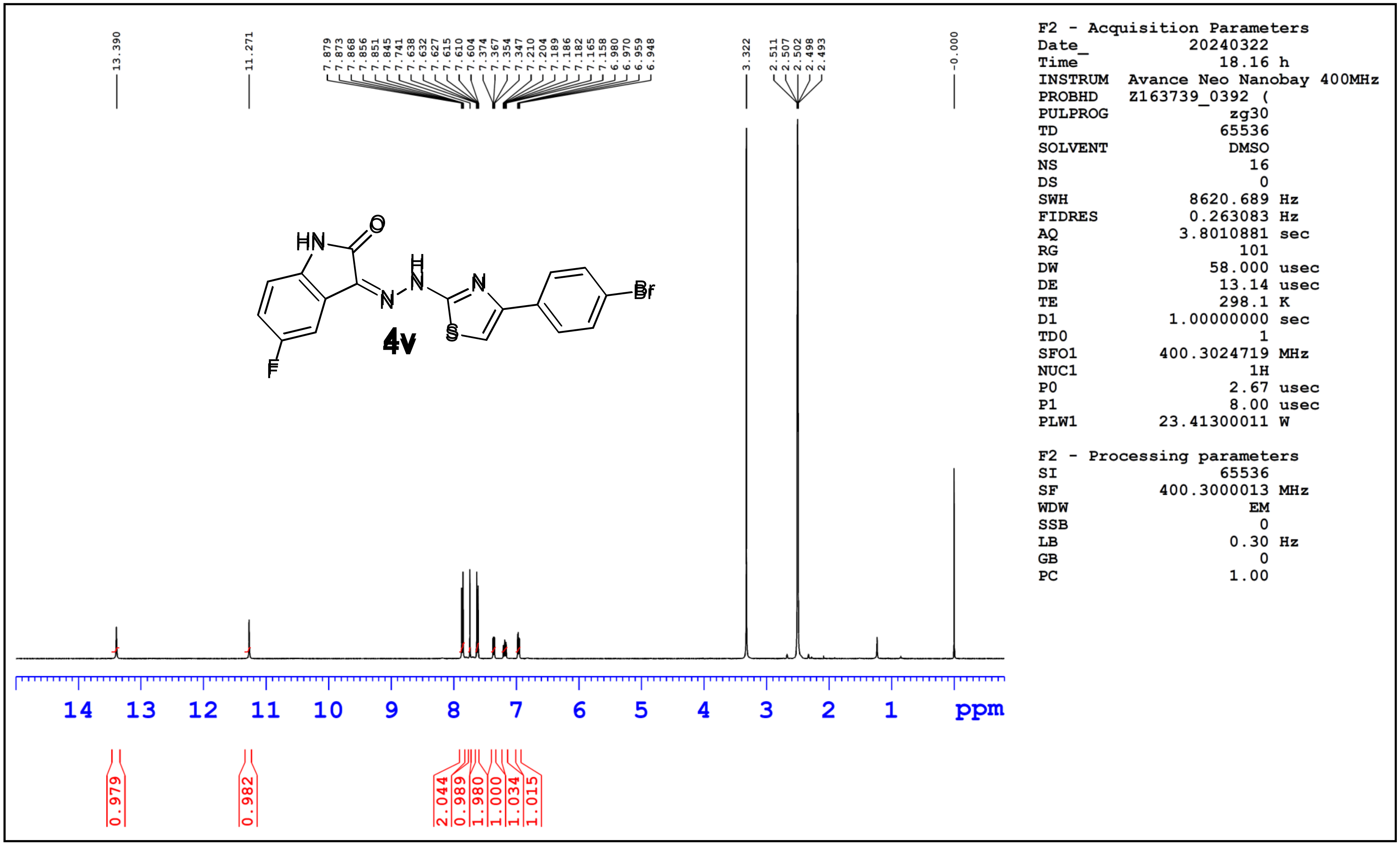

Figure S60^{1}H NMR spectrum of 4v.

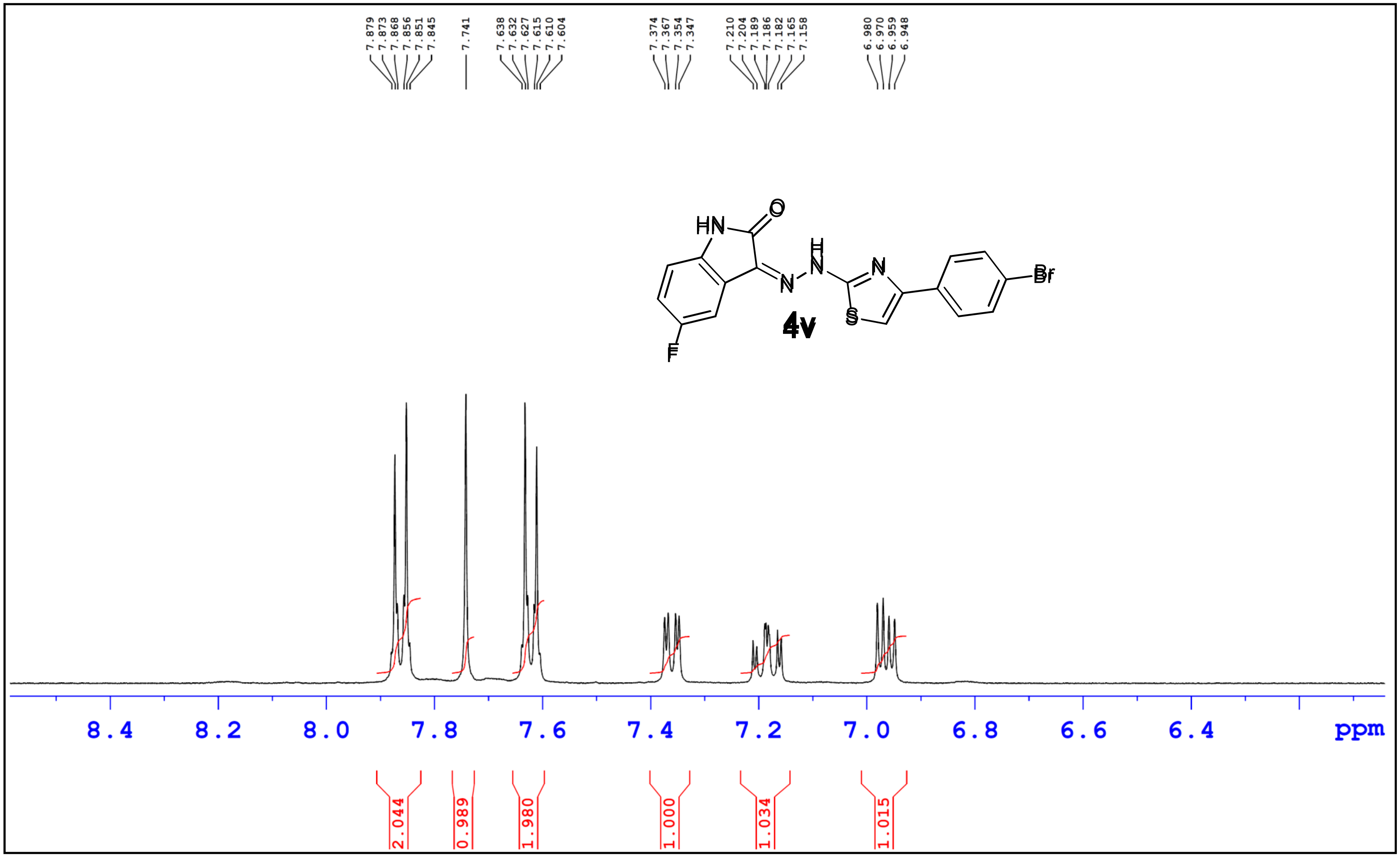

Figure S61 Expanded ^{1}H NMR spectrum of 4v.

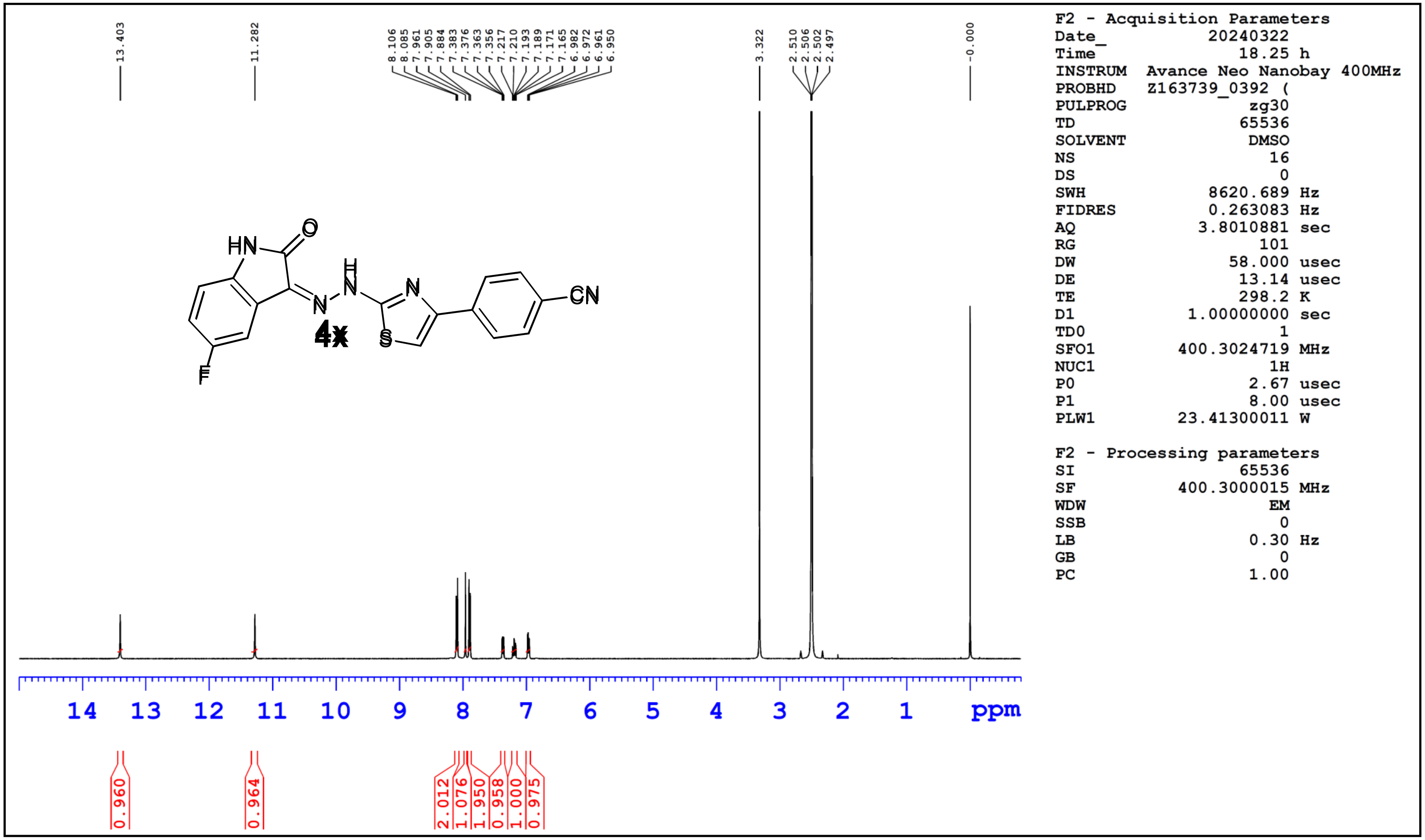

Figure S62^{1}H NMR spectrum of 4x.

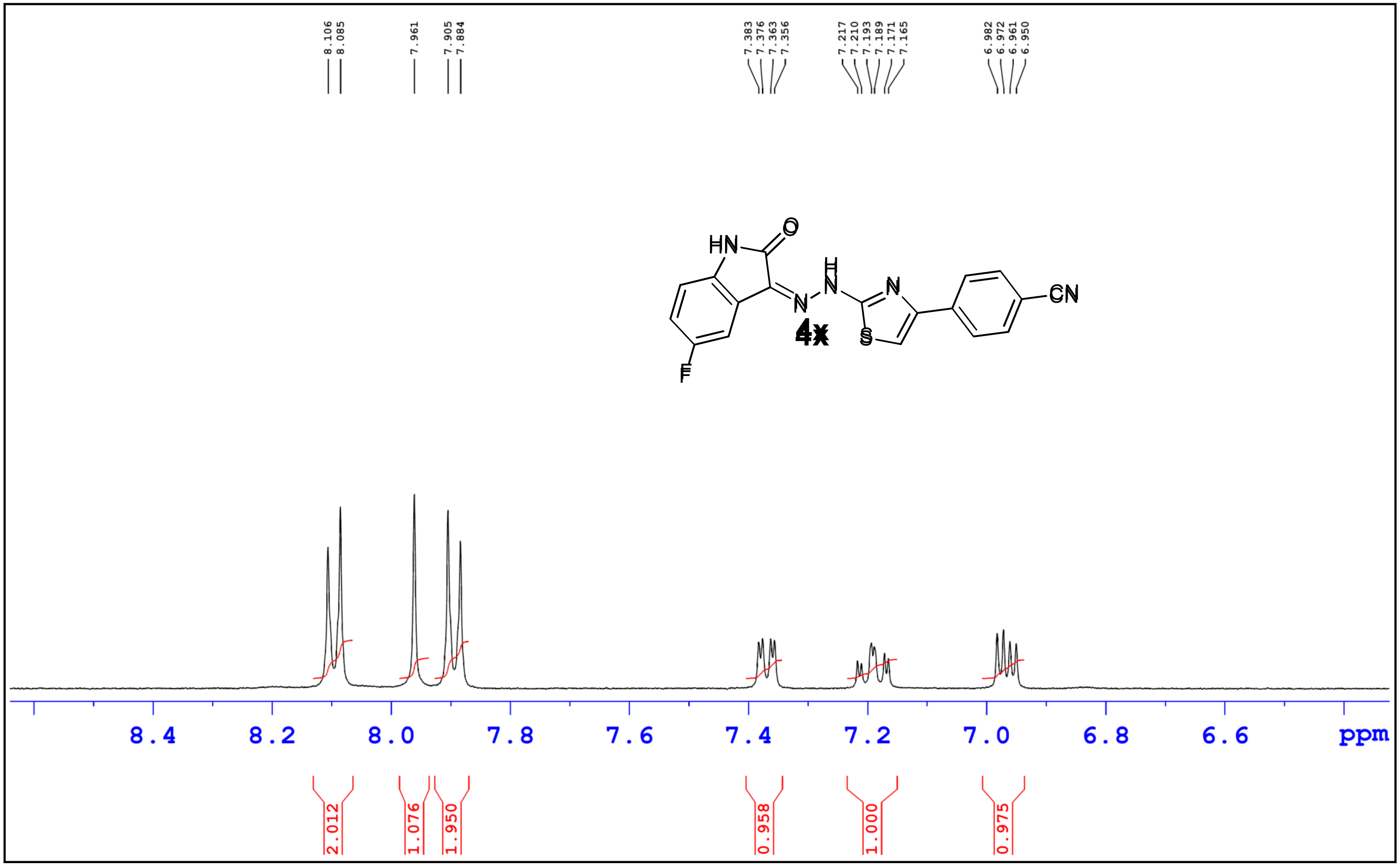

Figure S63Expanded ^{1}H NMR spectrum of 4x.

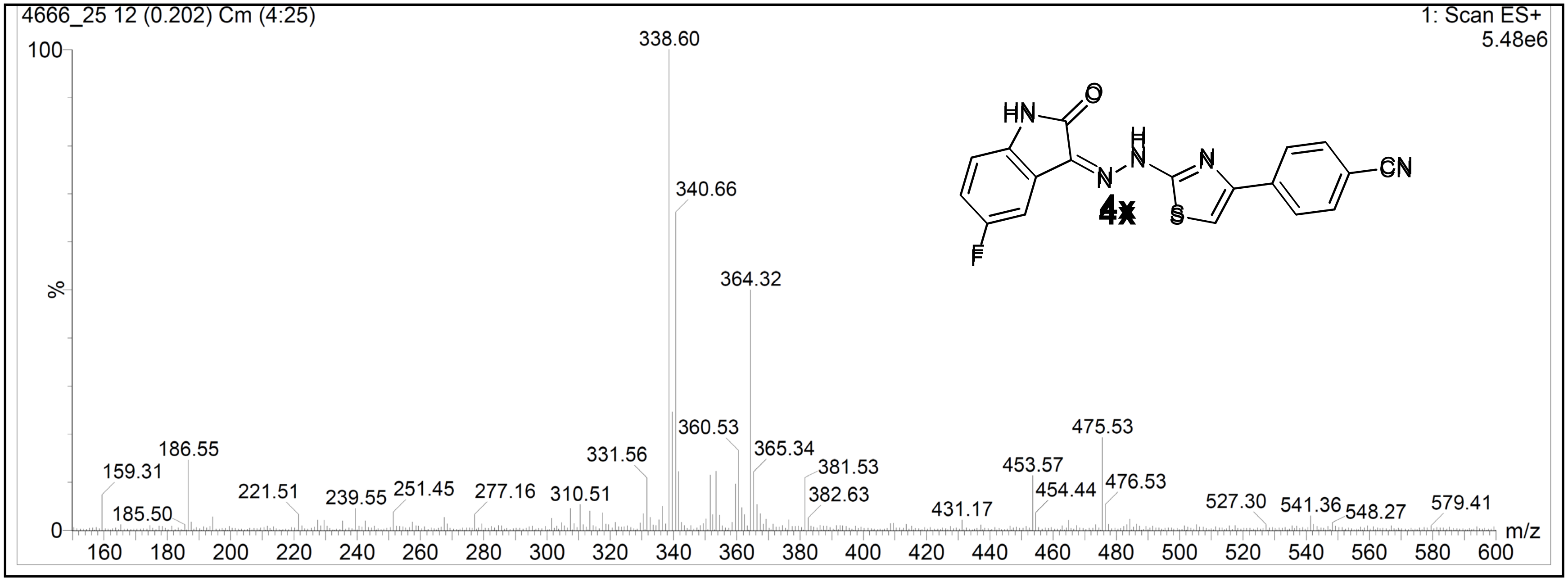

Figure S64 Mass spectrum of 4x.

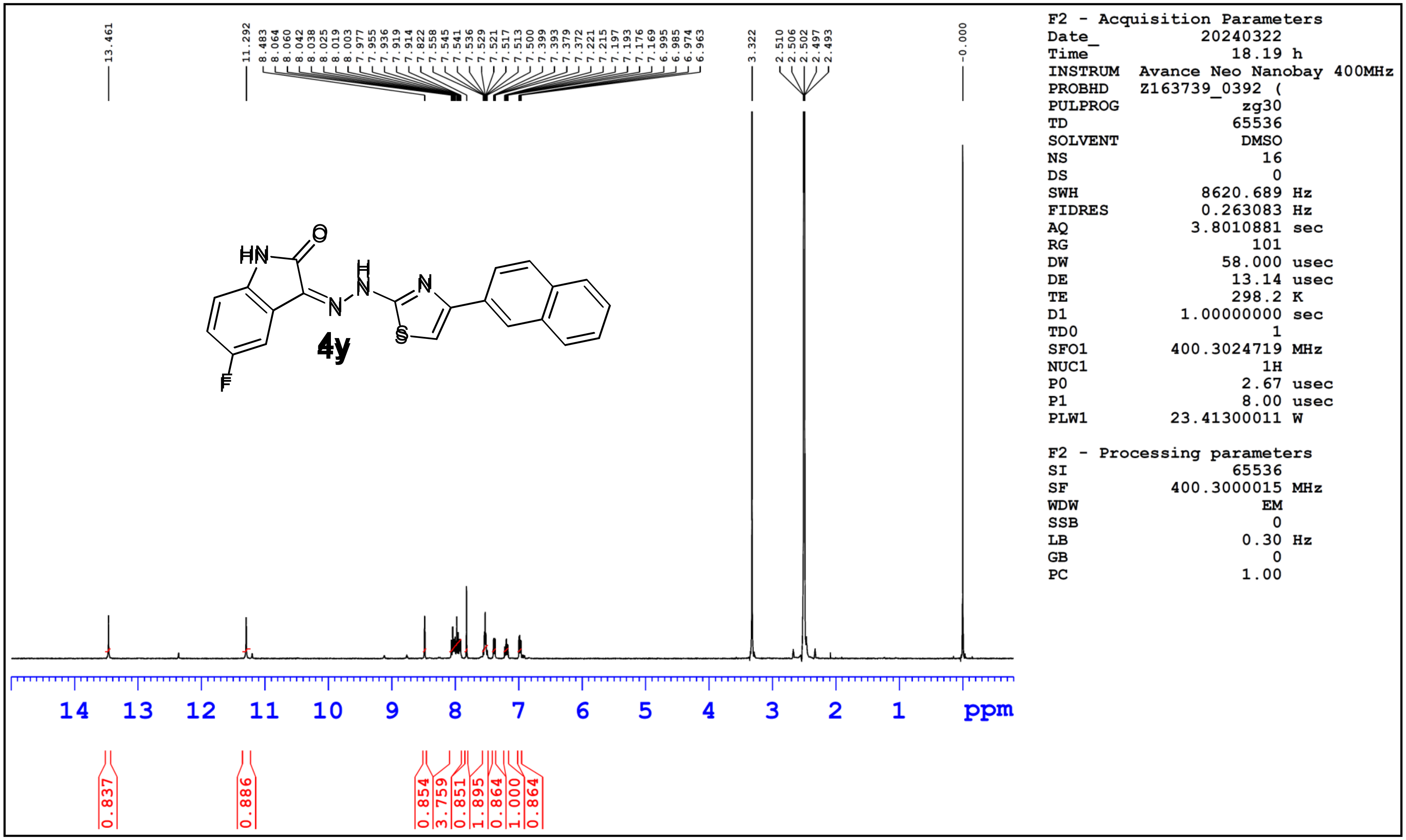

Figure S65^{1}H NMR spectrum of 4y.

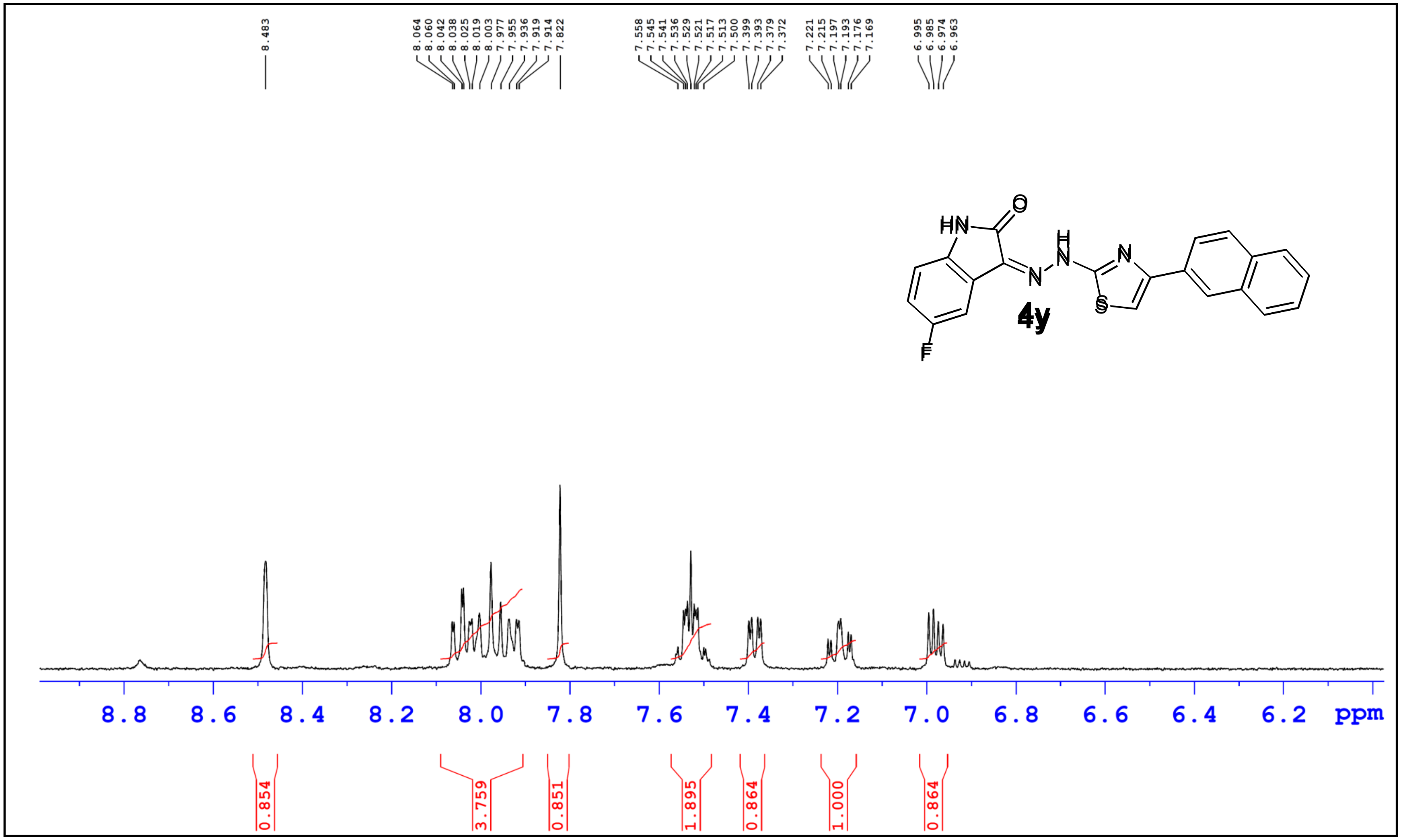

Figure S66Expanded ^{1}H NMR spectrum of 4y.

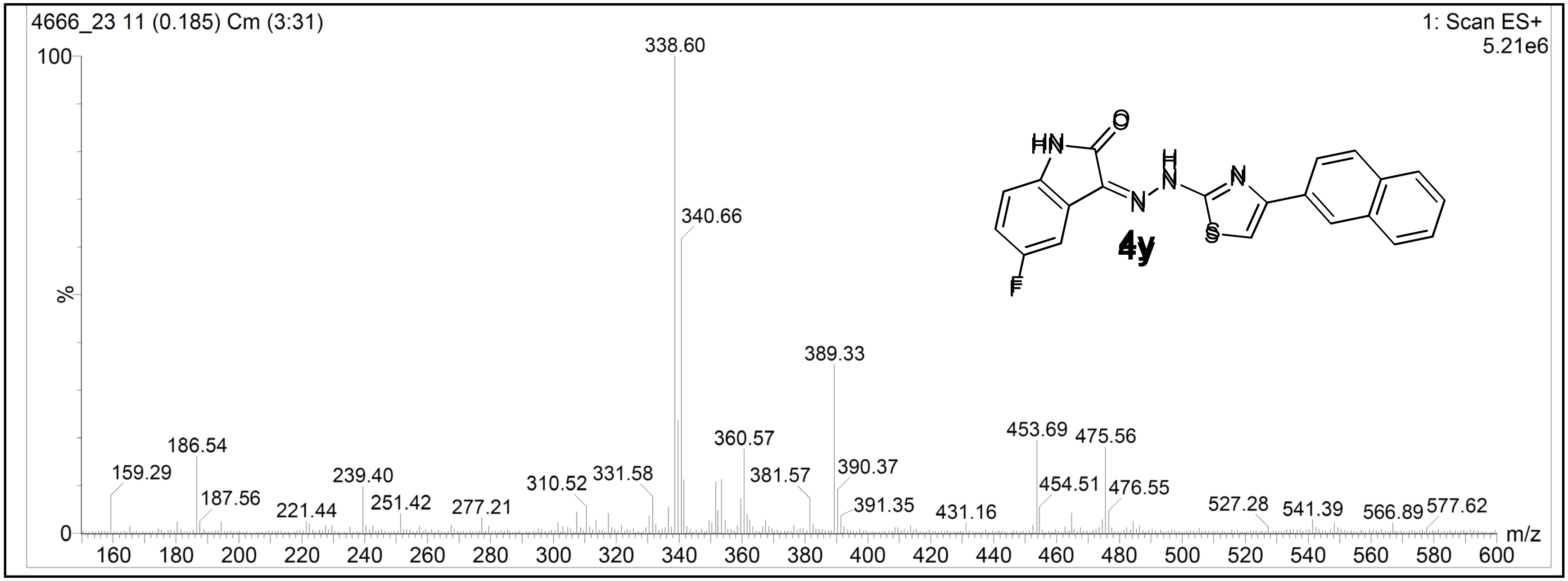

Figure S67 Mass spectrum of 4y.

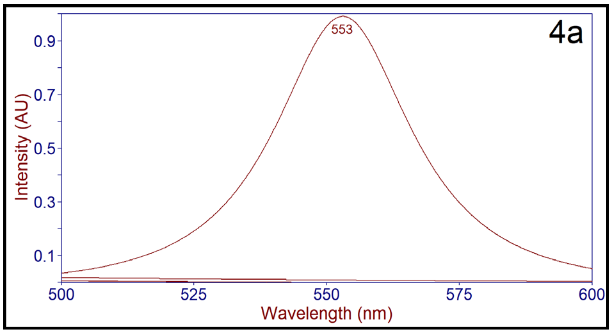
4a
553
Intensity (AU)
0.9
0.7
0.5
0.3
0.1
500
525
550
575
600
Wavelength (nm)
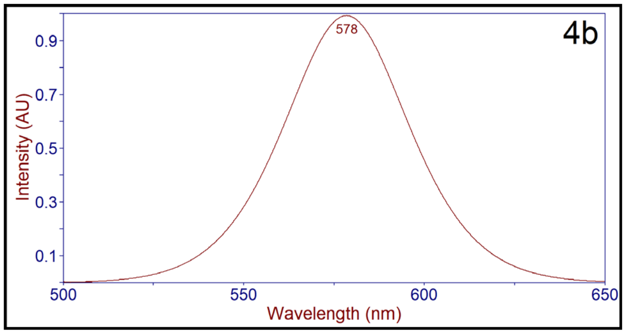
4b
578
Intensity (AU)
0.9
0.7
0.5
0.3
0.1
500
550
600
650
Wavelength (nm)
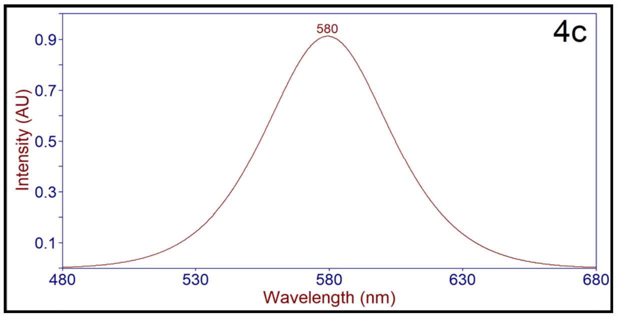
4c
580
Intensity (AU)
0.9
0.7
0.5
0.3
0.1
480
530
580
630
680
Wavelength (nm)
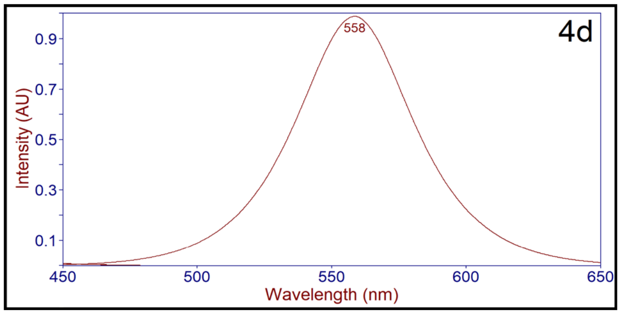
4d
558
Intensity (AU)
0.9
0.7
0.5
0.3
0.1
450
500
550
600
650
Wavelength (nm)
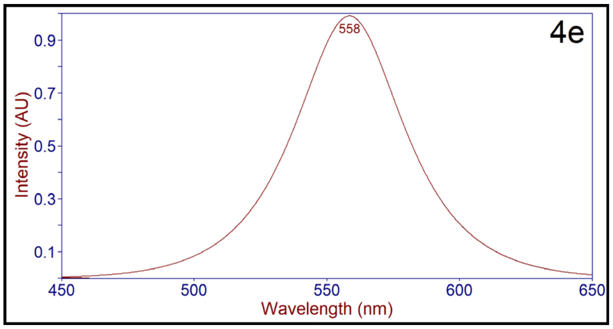
4e
558
Intensity (AU)
0.9
0.7
0.5
0.3
0.1
450
500
550
600
650
Wavelength (nm)
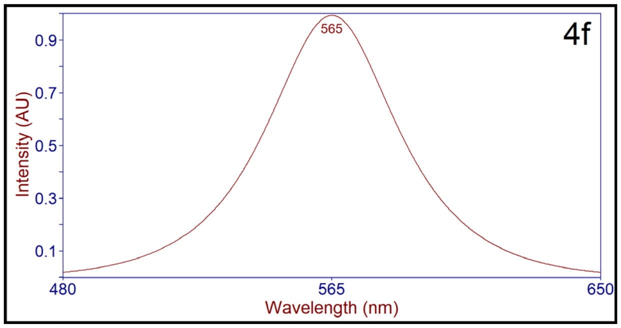
4f
565
Intensity (AU)
0.9
0.7
0.5
0.3
0.1
480
565
650
Wavelength (nm)
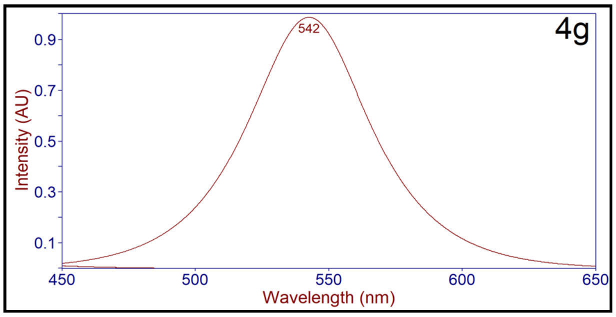
4g
542
Intensity (AU)
0.9
0.7
0.5
0.3
0.1
450
500
550
600
650
Wavelength (nm)
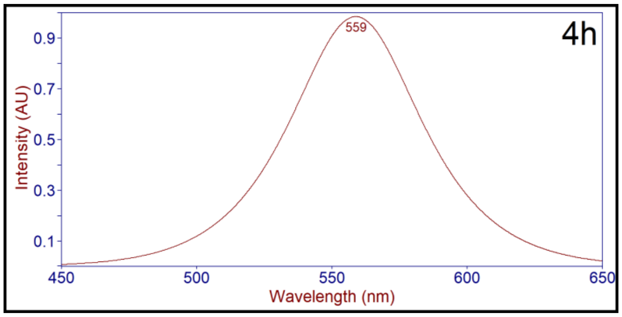
4h
559
Intensity (AU)
0.9
0.7
0.5
0.3
0.1
450
500
550
600
650
Wavelength (nm)
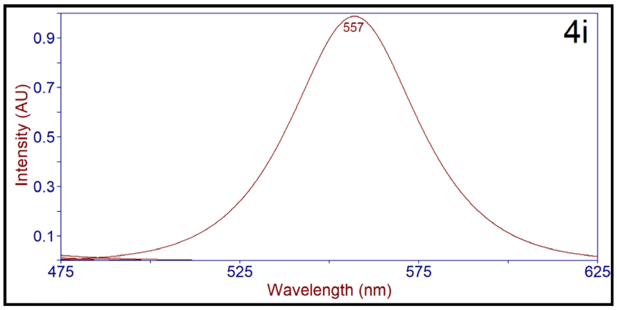
4i
557
Intensity (AU)
0.9
0.7
0.5
0.3
0.1
475
525
575
625
Wavelength (nm)
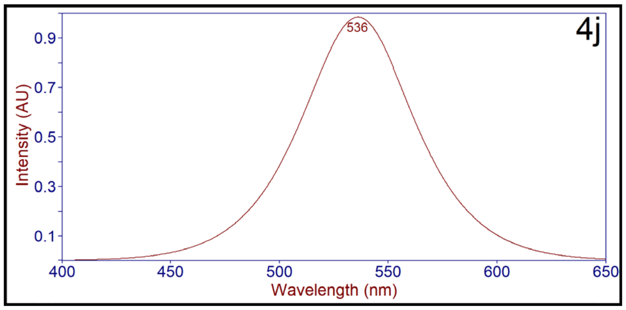
4j
536
Intensity (AU)
0.9
0.7
0.5
0.3
0.1
400
450
500
550
600
650
Wavelength (nm)

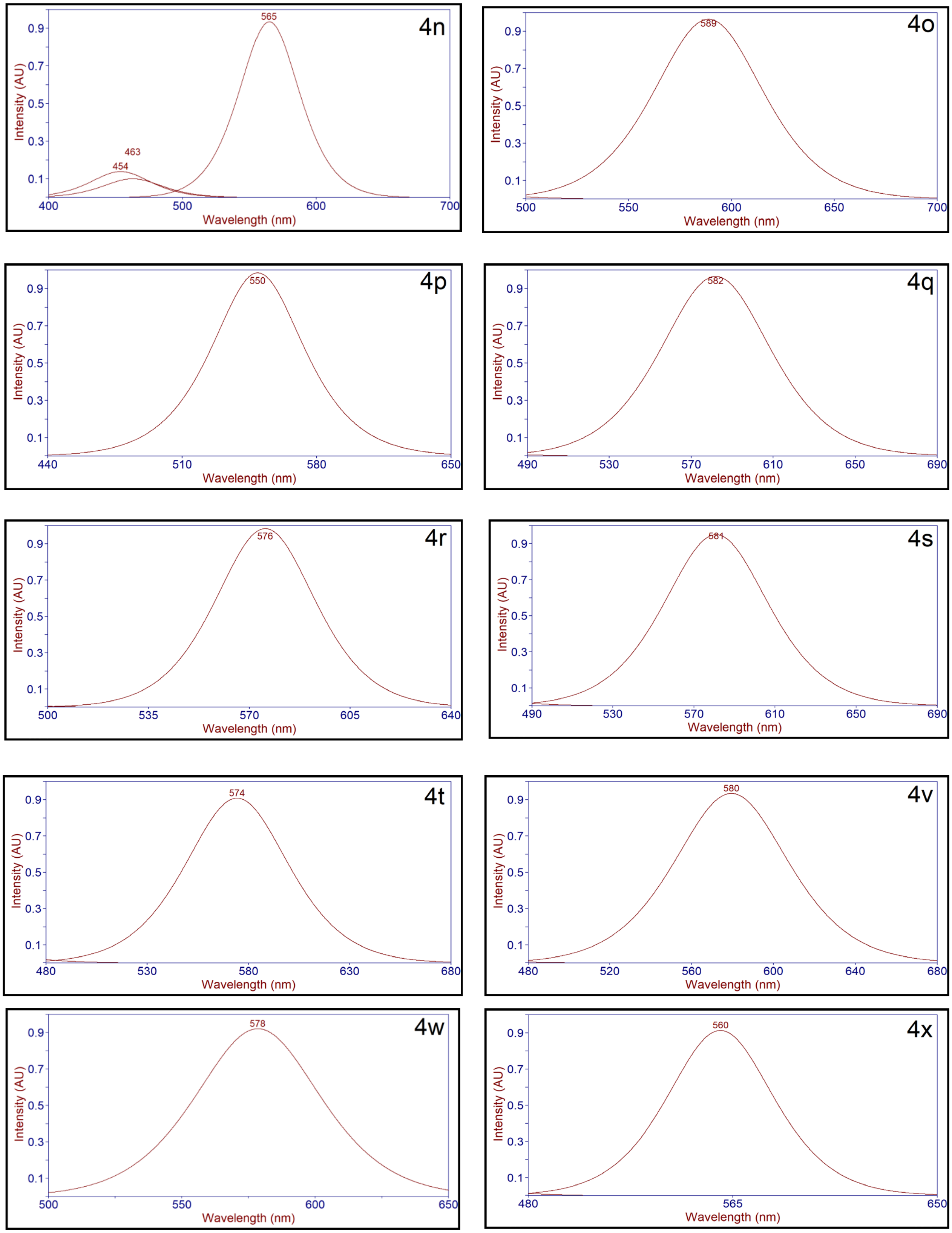

Figure S68. Deconvoluted solid state emission spectra of **4a-4j, 4n-4t & 4v-4x.**

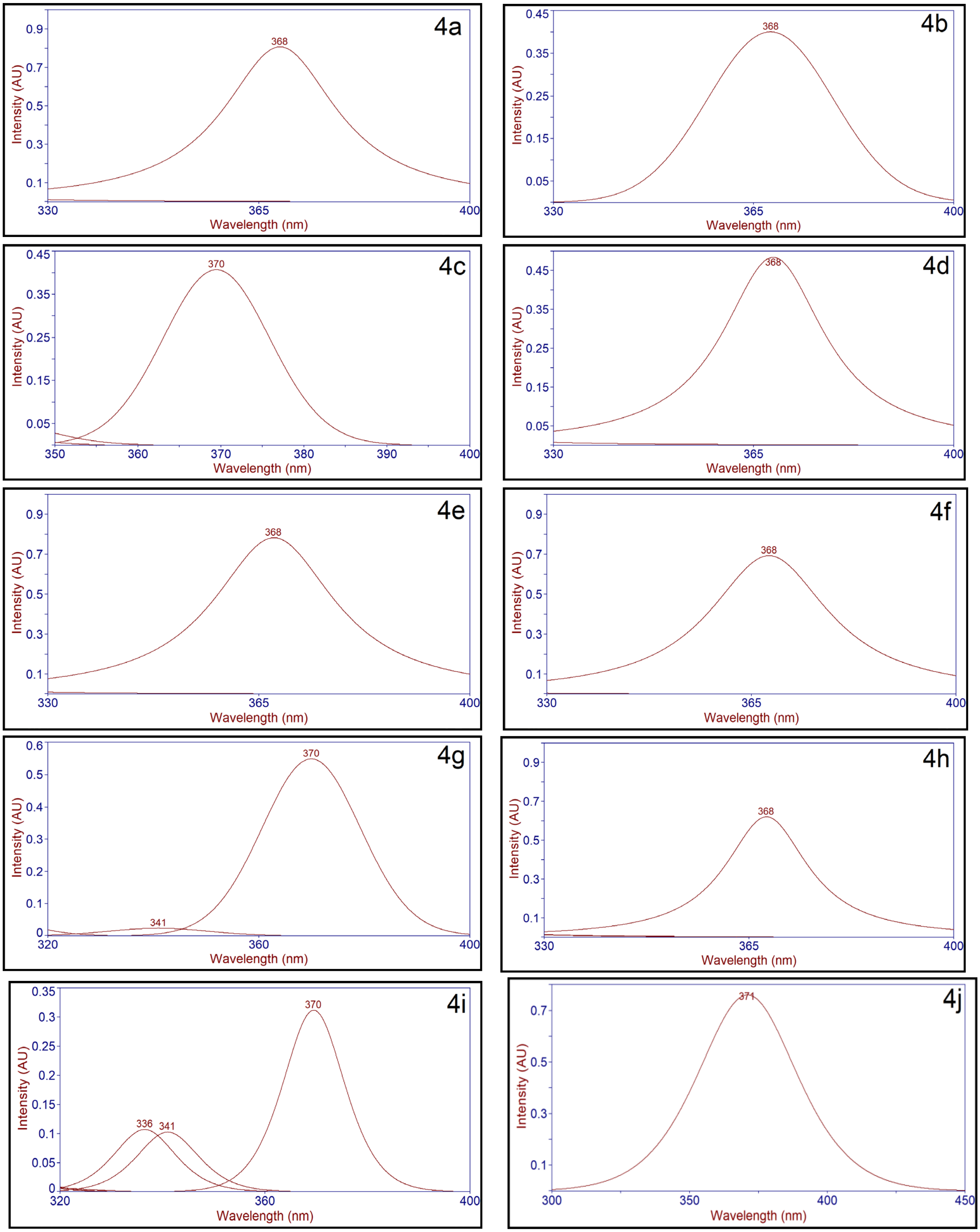
4a
368
Intensity (AU)
0.9
0.7
0.5
0.3
0.1
330
365
400
Wavelength (nm)
4b
368
Intensity (AU)
0.45
0.35
0.25
0.15
0.05
330
365
400
Wavelength (nm)
4c
370
Intensity (AU)
0.45
0.35
0.25
0.15
0.05
350
360
370
380
390
400
Wavelength (nm)
4d
368
Intensity (AU)
0.45
0.35
0.25
0.15
0.05
330
365
400
Wavelength (nm)
4e
368
Intensity (AU)
0.9
0.7
0.5
0.3
0.1
330
365
400
Wavelength (nm)
4f
368
Intensity (AU)
0.9
0.7
0.5
0.3
0.1
330
365
400
Wavelength (nm)
4g
370
341
Intensity (AU)
0.6
0.5
0.4
0.3
0.2
0.1
0
320
360
400
Wavelength (nm)
4h
368
Intensity (AU)
0.9
0.7
0.5
0.3
0.1
330
365
400
Wavelength (nm)
4i
370
336
341
Intensity (AU)
0.35
0.3
0.25
0.2
0.15
0.1
0.05
0
320
360
400
Wavelength (nm)
4j
371
Intensity (AU)
0.7
0.5
0.3
0.1
300
350
400
450
Wavelength (nm)

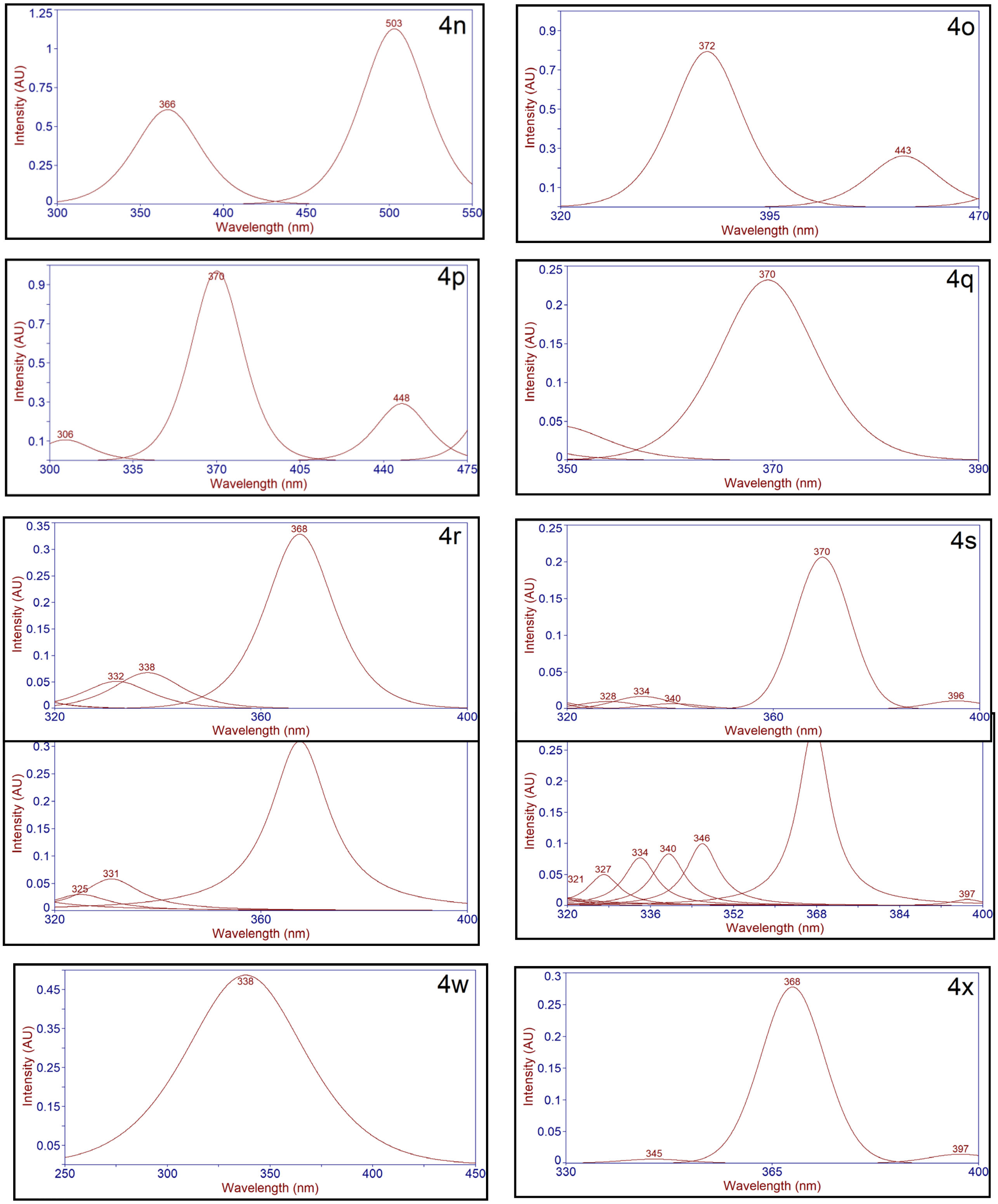

Figure S69. Deconvoluted solid state excitation spectra of **4a-4j, 4n-4t** & **4v-4x.**

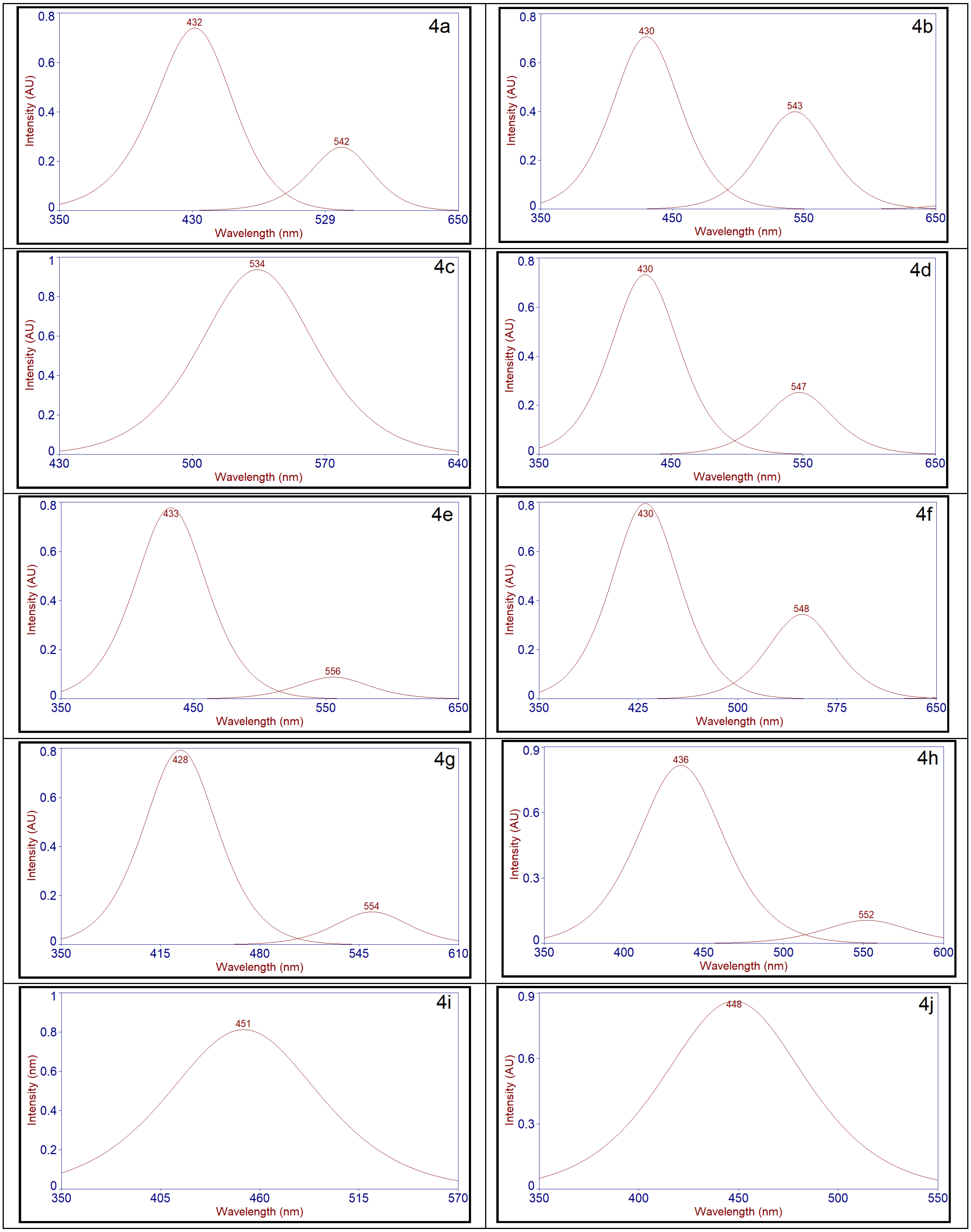
4a
432
542
Intensity (AU)
Wavelength (nm)
350 430 529 650
0 0.2 0.4 0.6 0.8
4b
430
543
Intensity (AU)
Wavelength (nm)
350 450 550 650
0 0.2 0.4 0.6 0.8
4c
534
Intensity (AU)
Wavelength (nm)
430 500 570 640
0 0.2 0.4 0.6 0.8 1
4d
430
547
Intensity (AU)
Wavelength (nm)
350 450 550 650
0 0.2 0.4 0.6 0.8
4e
433
556
Intensity (AU)
Wavelength (nm)
350 450 550 650
0 0.2 0.4 0.6 0.8
4f
430
548
Intensity (AU)
Wavelength (nm)
350 425 500 575 650
0 0.2 0.4 0.6 0.8
4g
428
554
Intensity (AU)
Wavelength (nm)
350 415 480 545 610
0 0.2 0.4 0.6 0.8
4h
436
552
Intensity (AU)
Wavelength (nm)
350 400 450 500 550 600
0 0.3 0.6 0.9
4i
451
Intensity (nm)
Wavelength (nm)
350 405 460 515 570
0 0.2 0.4 0.6 0.8 1
4j
448
Intensity (AU)
Wavelength (nm)
350 400 450 500 550
0 0.3 0.6 0.9

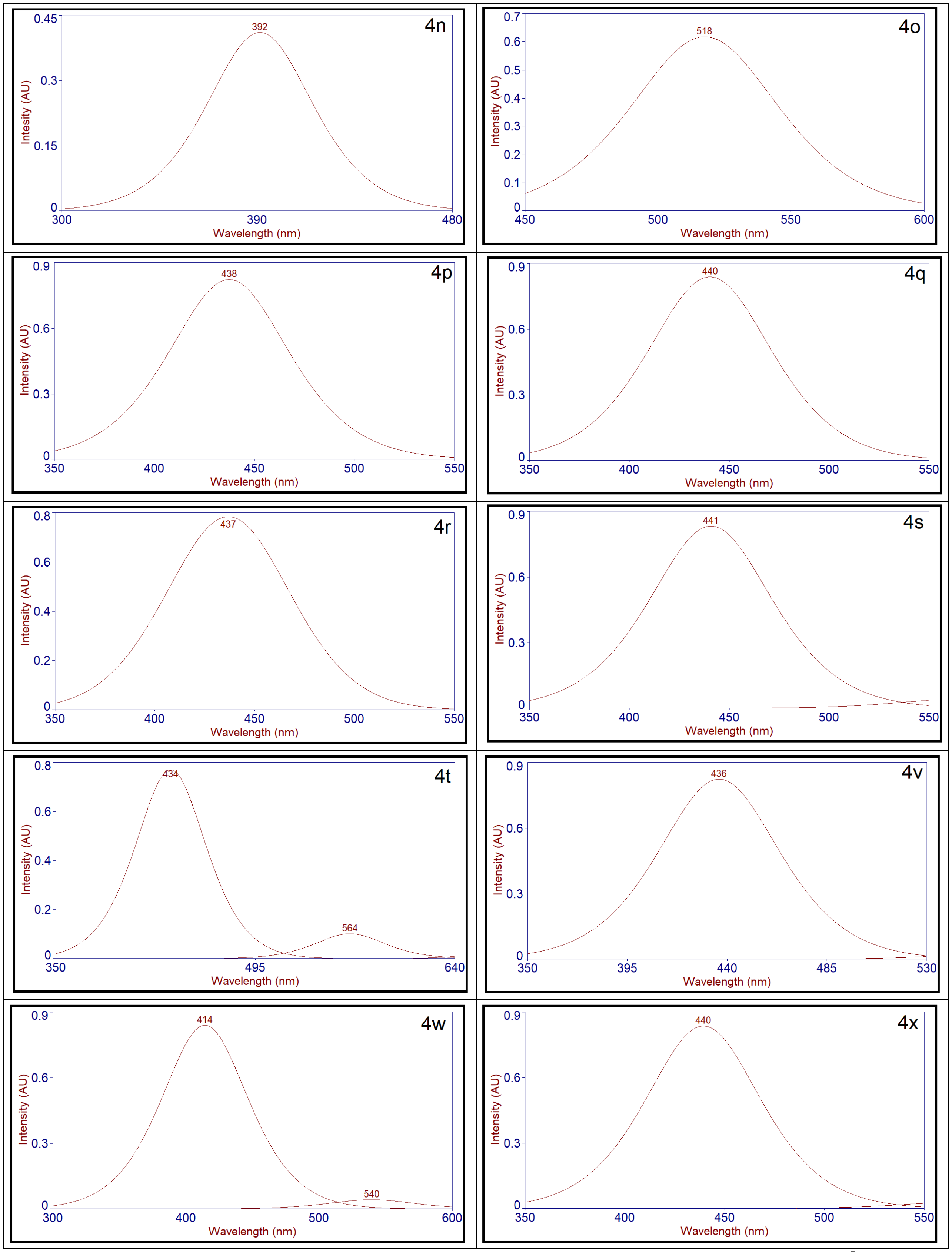

Figure S70. Deconvoluted emission spectra of **4a-4j, 4n-4t** & **4v-4x** in DMSO (1.0x10^{-5} M)

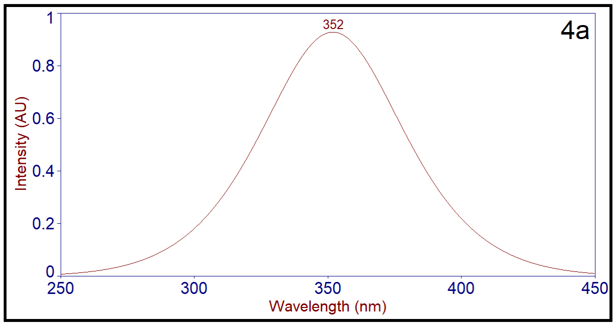
4a
352
Intensity (AU)
1
0.8
0.6
0.4
0.2
0
250
300
350
400
450
Wavelength (nm)

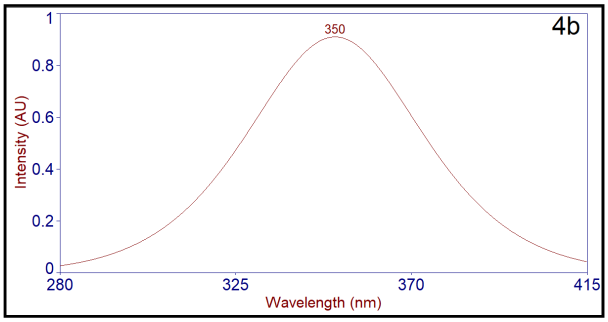
4b
350
Intensity (AU)
1
0.8
0.6
0.4
0.2
0
280
325
370
415
Wavelength (nm)

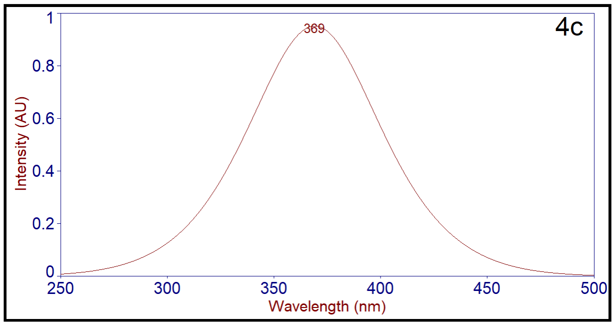
4c
369
Intensity (AU)
1
0.8
0.6
0.4
0.2
0
250
300
350
400
450
500
Wavelength (nm)

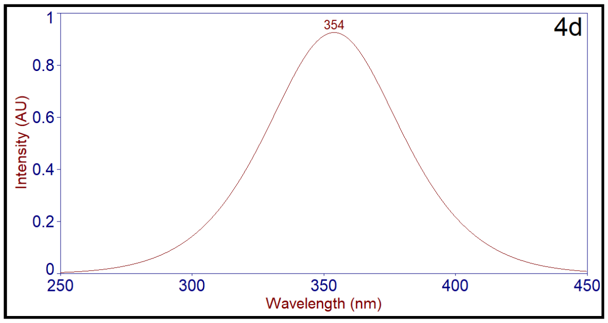
4d
354
Intensity (AU)
1
0.8
0.6
0.4
0.2
0
250
300
350
400
450
Wavelength (nm)

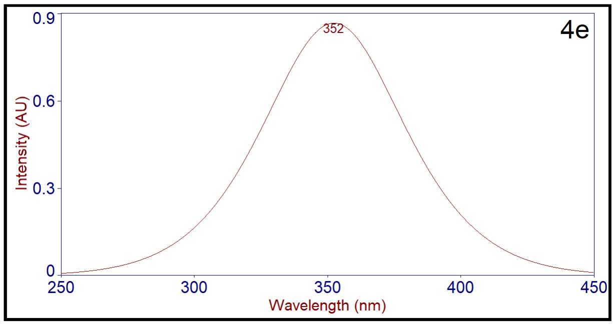
4e
352
Intensity (AU)
0.9
0.6
0.3
0
250
300
350
400
450
Wavelength (nm)

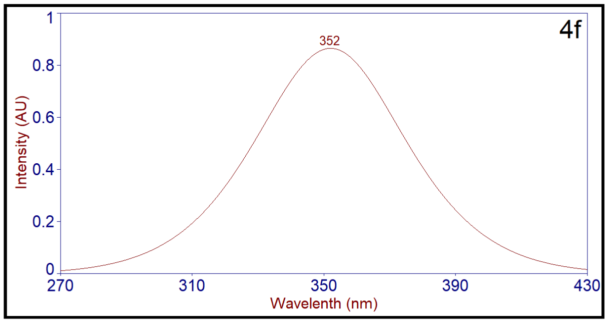
4f
352
Intensity (AU)
1
0.8
0.6
0.4
0.2
0
270
310
350
390
430
Wavelenth (nm)

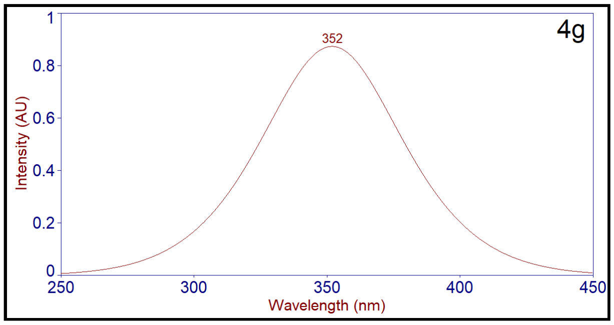
4g
352
Intensity (AU)
1
0.8
0.6
0.4
0.2
0
250
300
350
400
450
Wavelength (nm)

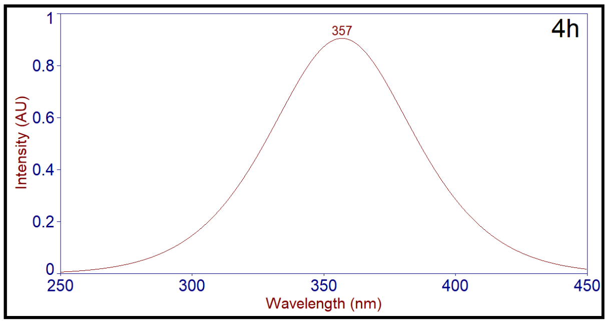
4h
357
Intensity (AU)
1
0.8
0.6
0.4
0.2
0
250
300
350
400
450
Wavelength (nm)

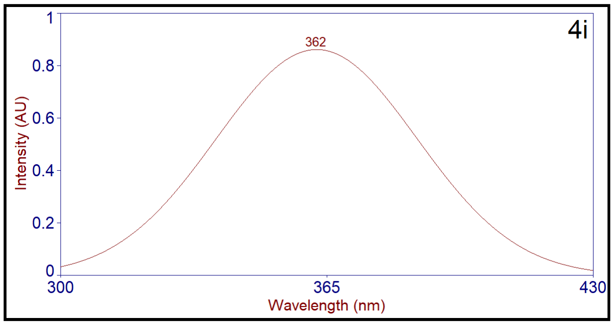
4i
362
Intensity (AU)
1
0.8
0.6
0.4
0.2
0
300
365
430
Wavelength (nm)

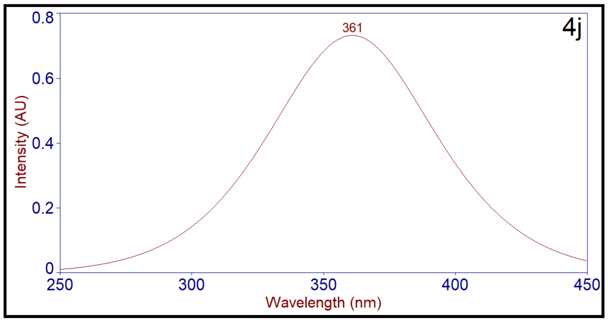
4j
361
Intensity (AU)
0.8
0.6
0.4
0.2
0
250
300
350
400
450
Wavelength (nm)

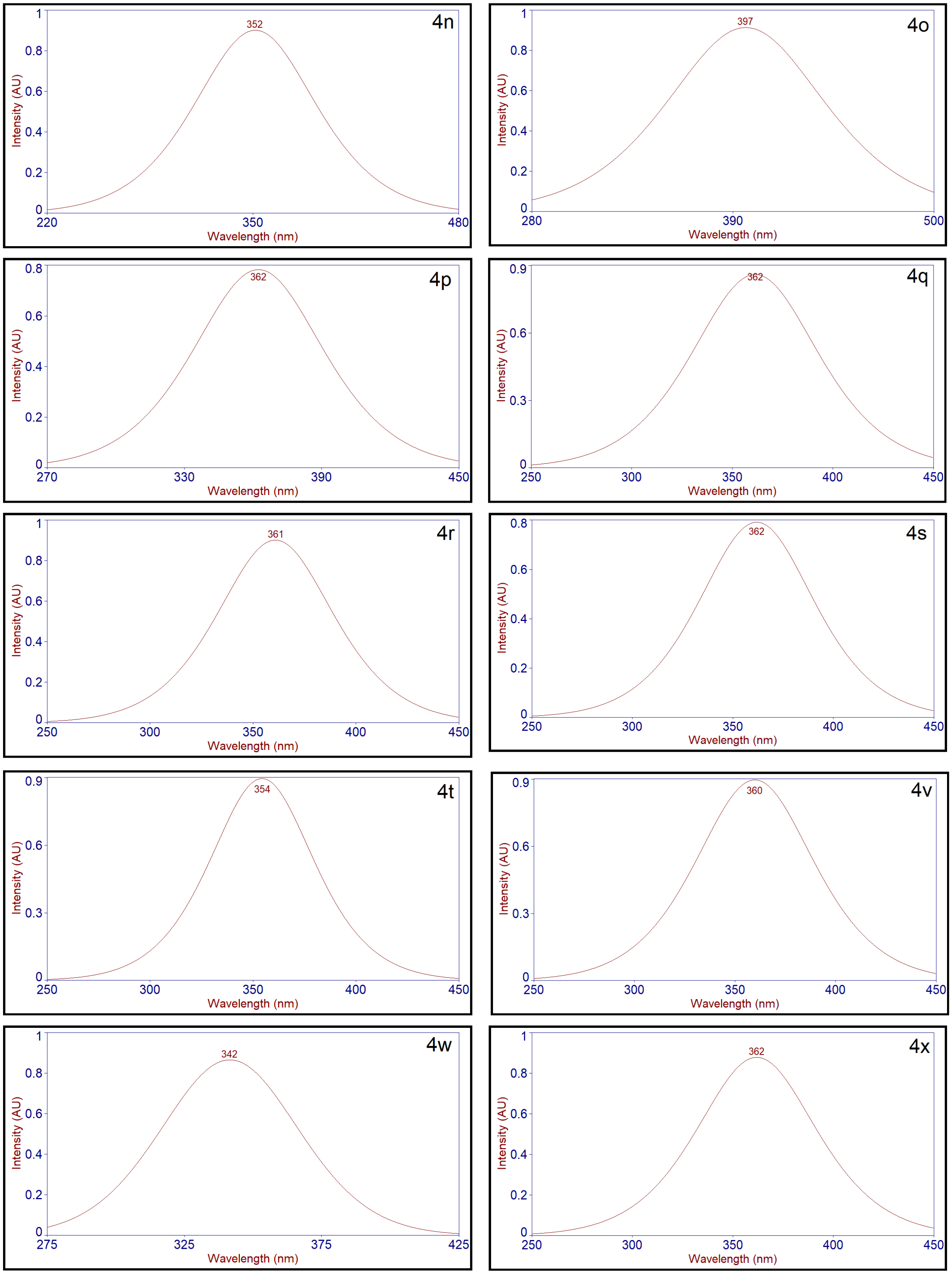

Figure S71. Deconvoluted absorption spectra of **4a-4j, 4n-4t** & **4v-4x** in DMSO ($1.0x10^{-5}$ M)

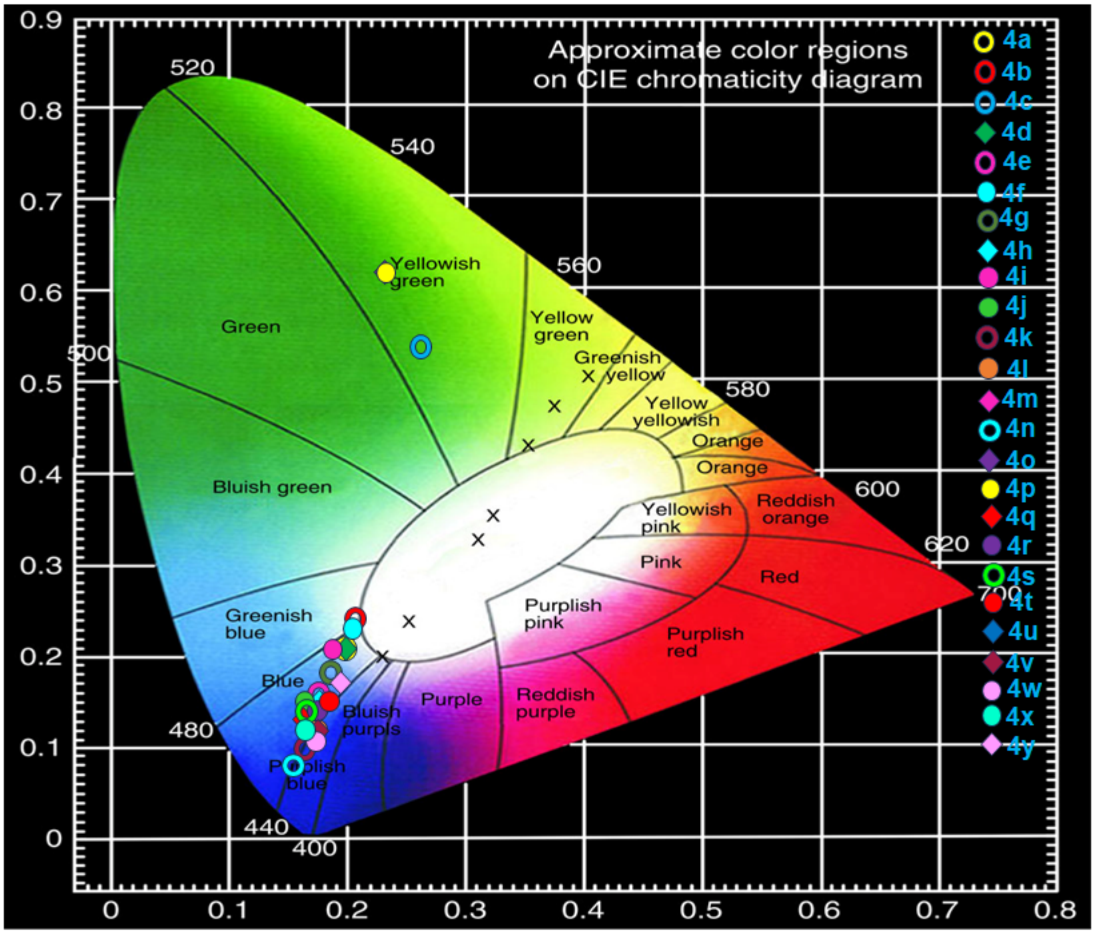

Figure S72. CIE chromaticity diagram of thiazolylhydrazonoindolin-2-ones (**4**) in the solution state.
